# Dynamic behaviour of monohaptoallylpalladium species: internal coordination as a driving force in allylic alkylation chemistry†
†Electronic supplementary information (ESI) available: Experimental procedures, X-ray crystallographic data, characterization data, chiral chromatographic analyses, and computational details. CCDC 991087. For ESI and crystallographic data in CIF or other electronic format see DOI: 10.1039/c5sc01867f


**DOI:** 10.1039/c5sc01867f

**Published:** 2015-07-06

**Authors:** Lan-Gui Xie, Viktor Bagutski, Davide Audisio, Larry M. Wolf, Volker Schmidts, Kathrin Hofmann, Cornelia Wirtz, Walter Thiel, Christina M. Thiele, Nuno Maulide

**Affiliations:** a University of Vienna , Institute of Organic Chemistry , Währinger Strasse 38 , 1090 Vienna , Austria . Email: nuno.maulide@univie.ac.at; b Technische Universität Darmstadt , Clemens Schöpf Institut für Organische Chemie und Biochemie , Alarich-Weiss-Str. 4 , 64287 Darmstadt , Germany . Email: cthiele@thielelab.de; c Max-Planck-Institut für Kohlenforschung , Kaiser-Wilhelm-Platz 1 , 45470 Mülheim an der Ruhr , Germany; d Technische Universität Darmstadt , Eduard-Zintl-Institute , Alarich-Weiss-Str. 12 , 64287 Darmstadt , Germany

## Abstract

Contemporary catalytic procedures involving alkylpalladium(ii) have enriched the arsenal of synthetic organic chemistry. Those transformations usually rely on internal coordination through “directing groups”, carefully designed to maximize catalytic efficiency and regioselectivity. Herein, we report structural and reactivity studies of a series of internally coordinated monohaptoallylpalladium complexes. These species enable the direct spectroscopic observation and theoretical study of π–σ–π interconversion processes. They further display unusual dynamic behavior which should be of direct relevance to chemistries beyond catalytic allylic alkylation.

## Abstract 2

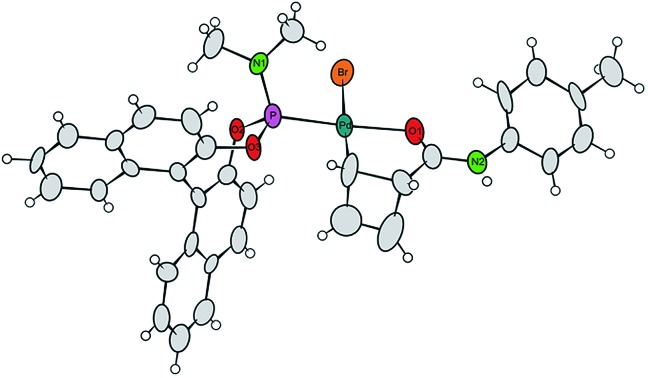
Structural and reactivity studies of internally coordinated monohaptoallylpalladium(ii) complexes.

## Introduction

Recent developments in catalysis involving alkylpalladium(ii) intermediates have enabled a myriad of selective transformations in organic synthesis.^1^ Particularly widespread is the importance of such intermediates in C–H functionalizations of sp^3^ C–H bonds.^2^ Common in those chemistries is the requirement for pre-installed “directing groups”, which stabilize the intermediate metal centre through chelation and thus help to prevent decomposition.^2^

Understanding how such coordinating moieties can affect not only the structure of intermediate metal complexes but also the reaction pathways available to them is a valuable endeavour that might lead to the discovery of new reactivity.

**Scheme 1:**
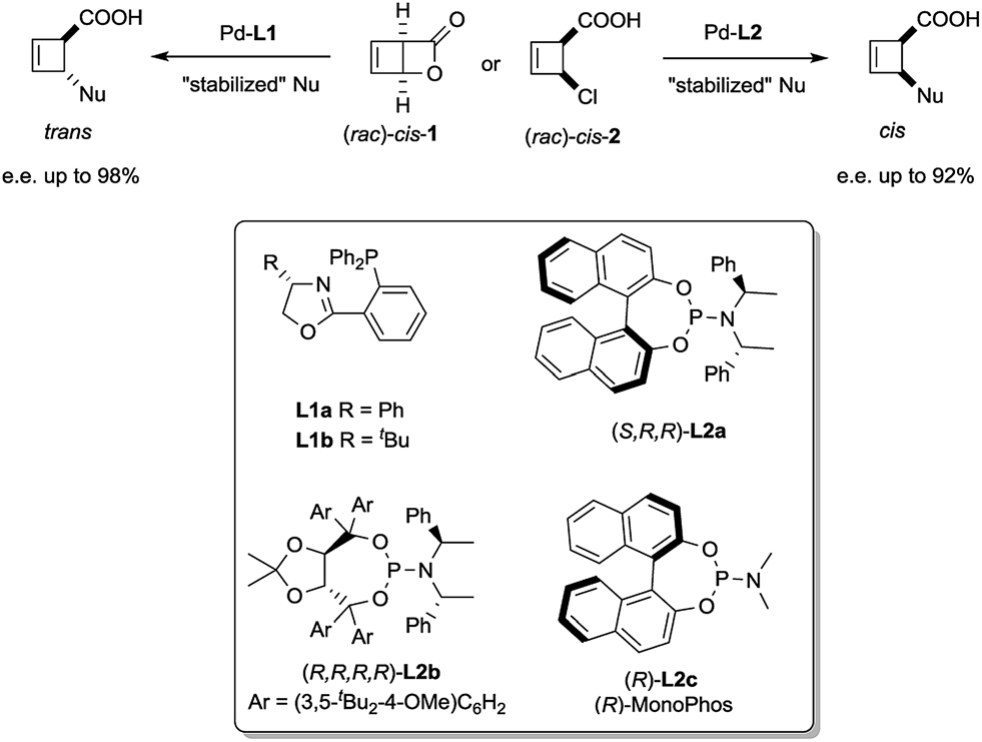
Stereoselective synthesis of *trans* and *cis*-disubstituted cyclobutenes from *cis*-**1** and *cis*-**2**.

We have recently reported a palladium-catalysed diastereodivergent asymmetric allylic alkylation on cyclobutene substrates.^3^ In that transformation, unusually strong ligand effects were observed that led, in the presence of stabilized carbanions and depending on the ligand employed, either to the products of overall retention or overall inversion of configuration (Scheme 1).

These unusual phenomena led us to investigate the reactivity of the putative allylpalladium intermediates in more detail. Employing the bidentate ligand **L1a**, we eventually identified a series of η^1^-allyl palladium complexes, prone to very facile electrocyclic ring opening at temperatures close to r.t., as key species in the deracemization process.^4^ We subsequently became interested in the case of monodentate ligands such as **L2a–c**. Herein we present our findings on the structure of internally chelated η^1^-allyl palladium complexes containing those ligands, their kinetic study enabling a direct insight into metallotropic equilibria, as well as a rare showcase of the value of chelation in allylic substitution chemistry.

## Results and discussion

**Scheme 2:**
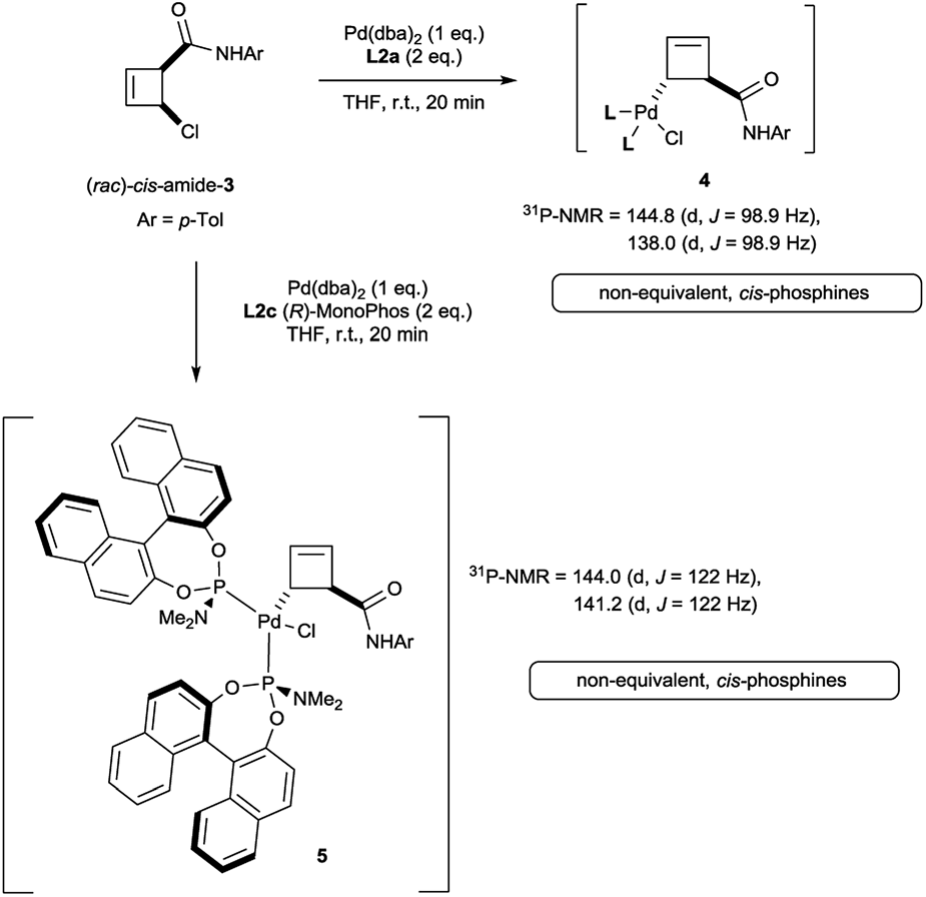
Initial results on the synthesis of allylpalladium(ii) complexes of chloroamide **3**.

Oxidative addition of Pd(dba)_2_ to the chloroamide (*rac*)-*cis*-**3** in the presence of 2 equivalents of ligand **L2a** proceeded to full conversion at room temperature (Scheme 2). The resulting organometallic species **4** was assigned as a single η^1^-coordinated allylpalladium complex by multinuclear NMR spectroscopy. ^31^P-NMR spectroscopy was diagnostic for two non-equivalent proximal phosphines attached to the presumed square-planar palladium(ii) centre. Replacement of **L2a** with the less bulky (and more amenable to detailed NMR analysis) MonoPhos ligand **L2c** similarly led to an intermediate of structure **5**. The stability of these complexes at room temperature stands in contrast to the temperature-sensitive nature of the analogous complexes of bidentate ligand **L1a**.^4^

**Scheme 3:**
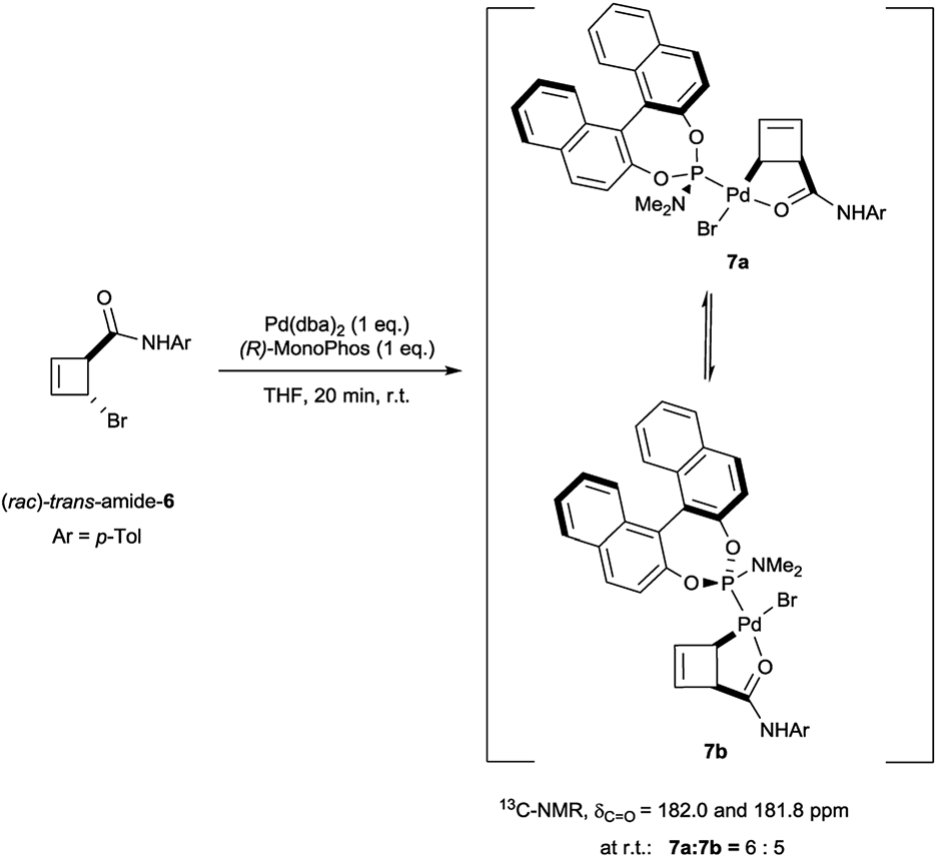
Formation of cyclobut-2-enyl η^1^-allyl complexes from *trans*-amide-**6**.

The *trans-rac*-bromoamide **6** (Scheme 3)^5^ smoothly reacts at room temperature with stoichiometric amounts of monodentate **L2c** and Pd(dba)_2_ to form *two* fairly stable, isomeric species **7a** and **7b** in 6 : 5 ratio. These compounds could be assigned as 1 : 1 Pd : **L2c** complexes with a *syn* relationship between the metal center and the cyclobutene substituent,^6^ confirming that oxidative addition occurs with inversion of configuration.^7^ As we will show, these are the two diastereomeric products of suprafacial allylic migration (metallotropic shift) of the Pd(**L2c**)Br-fragment across the cyclobutene ring. Remarkably (Scheme 3), the ^13^C-NMR resonance of the carbonyl carbon was shifted from *δ* = 167.6 ppm in starting material **6** to *δ* = 182.0 and 181.8 ppm in the complexes **7a** and **7b**. This strongly suggests internal coordination of the amide carbonyl as the fourth ligand of the tetra-coordinate sphere around palladium. Worthy of note, this mixture of isomers survived purification by silica gel column chromatography, though required an inert atmosphere.

**Fig. 1:**
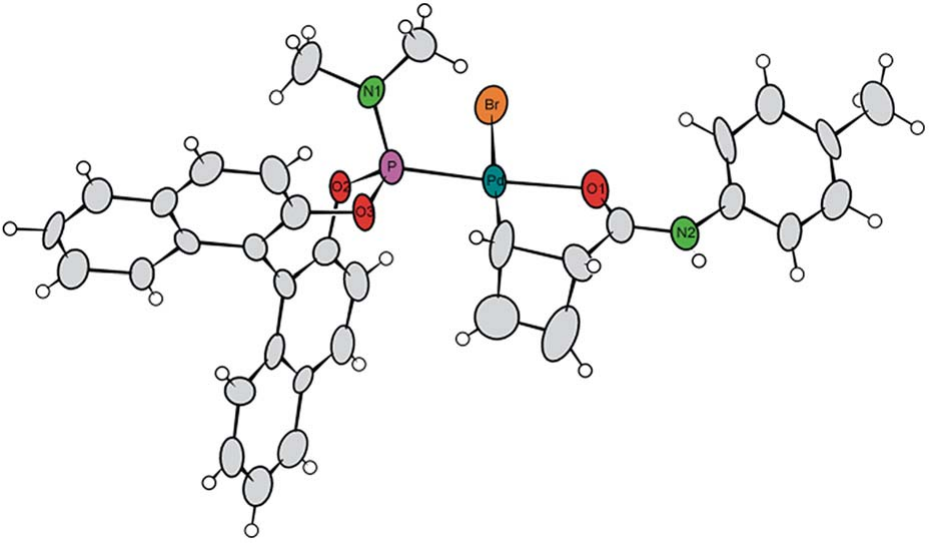
Projection of the molecular structure of the isomer **7a** (two crystal water molecules are omitted for clarity). Ellipsoids of the displacement parameters are drawn at 40% probability level.^8^

Single crystals of **7a** were obtained by crystallization of the crude mixture of **7a/7b** from CH_2_Cl_2_, and investigated by X-ray diffraction.^8^ The resulting molecular structure (Fig. 1) confirms the *syn*-orientation of substituents across the cyclobutene ring. The internal distances of the four-membered ring have typical lengths for localized C–C single (1.621 to 1.660 Å) and C=C double (1.365 Å) bonds. In contrast to our prior findings,^4^ the internal disubstituted C–C single bond is *shorter* than the other two, due to the chelation from the amide carbonyl. The short distance between the Pd-centre and the amide carbonyl oxygen (bond distance 2.08 Å) further validates this assumption. These two points are likely responsible for the remarkably enhanced stability of this compound at room temperature. To the best of our knowledge, crystal structures of internally coordinated monohaptoallyl-Pd complexes are not known in spite of the obvious relevance of such compounds even beyond the realm of catalytic allylic alkylation.^9^ Indeed, internally coordinated species such as **7** are postulated in virtually all established catalytic, directed C–H activation procedures; interestingly, amide coordination at Pd(ii) centre is most often observed or postulated to involve bonding through nitrogen9f,i rather than oxygen, as we observe in this case.

**Fig. 2:**
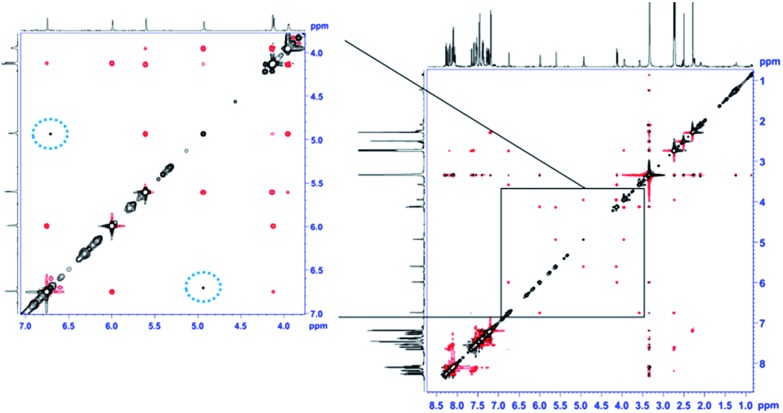
EASY-ROESY (*τ*_mix_ = 300 ms, 5 kHz spinlock field, 45° flip angle)^17^ spectrum of the isomeric mixture of **7a** and **7b** in DMSO-*d*_*6*_ at 300 K after covariance processing.^18^ The expansion shows the spectral region of the signals of the cyclobutene protons at a lower intensity level. NOE contacts between protons within the cyclobutene moiety of the same isomer show the opposite phase (red) as the diagonal signals (black). Cross peaks resulting from exchange of cyclobutene protons of different isomers (**7a** → **7b**, and *vice versa*) show the same phase as the diagonal (circled with a dotted blue line).

The unexpectedly selective crystallization of only one out of the two isomers **7a/7b** offers the possibility for direct observation of the metallotropic equilibrium of isomers **7a** and **7b** in solution.^10^ Additionally, solution conformations may differ from those determined in the solid state. Initial studies based on intramolecular NOEs provided useful guidance for the assignment of complexes **7a/7b** as allylic rearrangement isomers,^6^ but reliable quantification of interatomic distances relating the ligand and the cyclobutene fragments proved difficult, as only a few, weak NOE contacts were observed in the spectra (see Fig. 2). We thus sought to introduce the mixture of complexes **7a/b** into an anisotropic medium to measure residual dipolar couplings (RDC).^11^,^12^ After some experimentation we chose chemically cross-linked PDMS (polydimethylsiloxane)^13^ as an orienting medium. This choice yielded ω_1_-coupled HSQC^14^ spectra of excellent quality and enabled the identification of 8 C–H RDCs for each isomer.^6^

Structural models for the two isomers and the transitional η^3^-coordinated species were generated computationally by molecular modelling and subsequent geometry optimization by density functional theory (DFT) using ORCA.^15^ Fitting the experimental RDC data to the computed structure models of the isomers was performed with the RDC module of the hot-FCHT software package.^16^ Comparison of the experimental and back-calculated RDCs yielded excellent quality factors for the data assigned to the respective isomers, while all other combinations of experimental data and structure model showed a significantly worse fit.^6^ This validates the proposed structure models in solution.

Notably, the sparse solubility of **7a** in THF-*d*_*8*_ enabled the enrichment of an isomeric mixture of **7a**/**7b** up to 95% in **7a**, thus furnishing its clean NMR spectrum. Upon standing at room temperature, enriched **7a** gradually equilibrates back to the 6 : 5 thermodynamic ratio of isomers. This process was too slow in THF-*d*_*8*_ to be quantified by EXSY. Nevertheless, in DMSO-*d*_*6*_ we were able to qualitatively follow this exchange process by 2D EASY-ROESY spectra (see Fig. 2).^16^ Using less measurement-time-consuming, selective 1D PFGSE NOE spectra, we quantified activation parameters of that equilibrium at 320 K of Δ*G*^≠^ = 21.1 kcal mol^–1^ for **7a** → **7b** and 20.7 kcal mol^–1^ for the reverse process.^6^

**Fig. 3:**
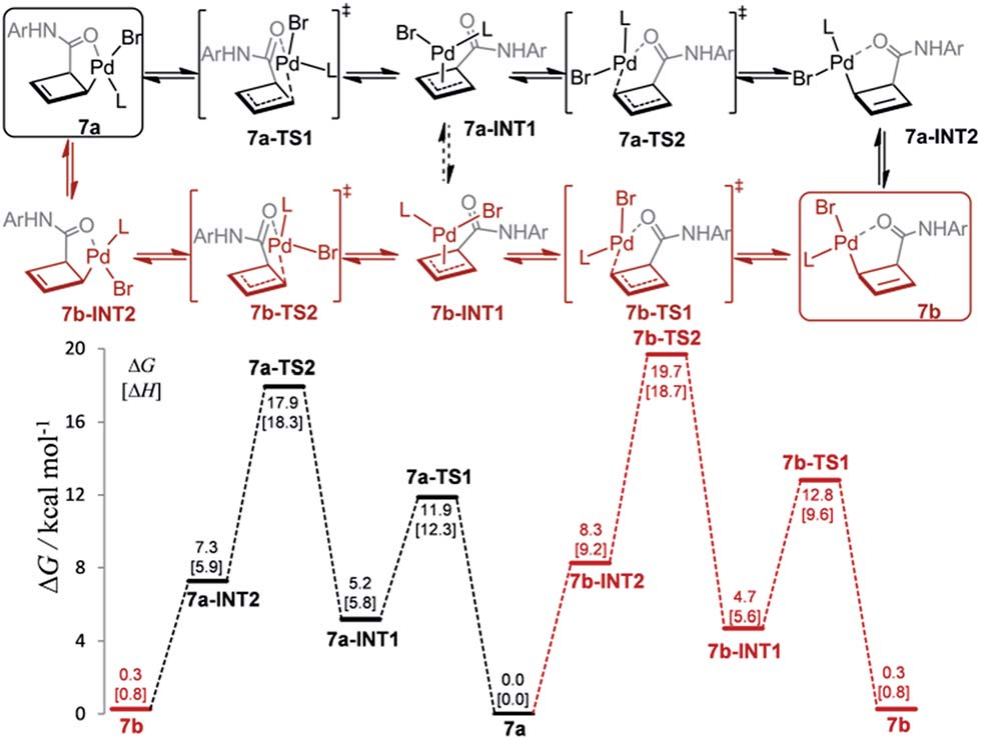
Computed Gibbs free energy profile (298.15 K) for the equilibration of **7a** and **7b** at the SMD(THF)-B3LYP-D3/def2-TZVP//B3LYP-D3/def2-SVP(def2-TZVP for Pd) level *via* two possible pathways. **L** = **L2c**.

The mechanism for this apparent metallotropic shift was investigated computationally at the B3LYP-D3 level (Fig. 3). The predicted free energy difference between **7a** and **7b** (–0.3 kcal mol^–1^) is very small, which is consistent with the observed 6 : 5 equilibrium ratio of **7a**/**7b** (corresponding to a free energy difference of –0.1 kcal mol^–1^). Two potential pathways were investigated for their isomerization, the first of which proceeds initially *via* an η^1^ → η^3^ conversion to **7a-INT1** (black path). This η^3^ intermediate **7a-INT1** then can undergo an η^3^ → η^1^ conversion to form **7a-INT2** followed by positional isomerization to generate **7b**. Alternatively, **7a-INT1** could isomerize to **7b-INT1**, accessing the second pathway, *via* an apparent rotation which could be facilitated by coordinating solvent in the absence of excess ligand.^19^ A separate pathway accessible to **7a** proceeds first *via* positional isomerization followed by an analogous η^1^ → η^3^ → η^1^ shift (red path). The maximum heights of the two pathways differ by 1.7 kcal mol^–1^ in favor of the first pathway, which is caused by the contrasting steric effects imparted by the chiral ligand. Either pathway is expected to translate to a relatively facile equilibration of **7a** and **7b** at room temperature, consistent with the experimentally determined activation parameters.^20^

**Scheme 4:**
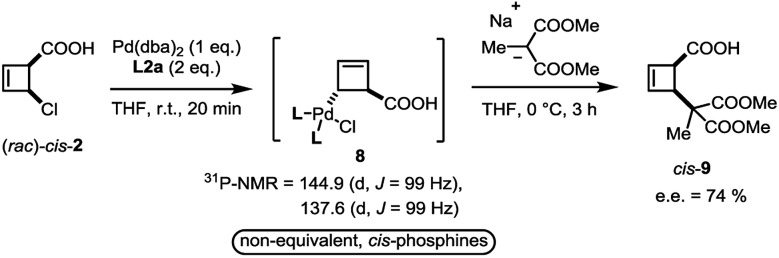
Formation of four membered ring η^1^-allyl complex from *cis*-**2**.

In close analogy with amide **3**, the (*rac*)-*cis*-chloroacid **2** also leads to the formation of a single *anti*-η^1^-allyl palladium complex **8** upon stoichiometric combination with Pd(dba)_2_ and **L2a** (Scheme 4). As the acid **2** had proved to be an ideal electrophile for catalytic deracemization in our previous work,^4^ we investigated the reaction of the monohaptoallylpalladium complex **8** with a suitable nucleophile. As shown, treatment of complex **8** with sodium (2-methyl)dimethylmalonate at 0 °C yielded the *cis*-disubstituted cyclobutene **9** in 74% ee. This result is consistent with the observed enantioselectivity in the catalytic process employing ligand **L2a**,^3^ thus demonstrating that **8** is a catalytically active intermediate.

**Fig. 4:**
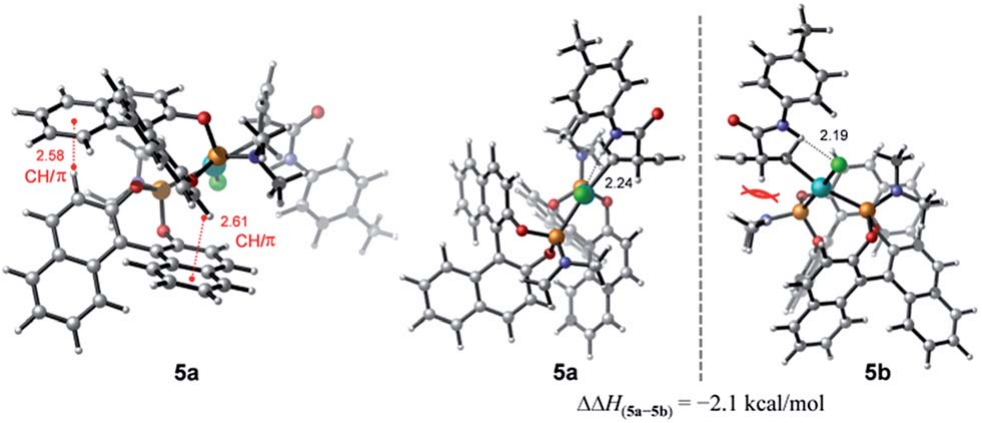
Computed diastereomers **5a** and **5b** and their corresponding enthalpy difference (ΔΔ*H*) obtained at the SMD(THF)-M06-D3/def2-TZVP//TPSS-D3/def2-SVP(def2-TZVP for Cl and Pd) level. Distances are in units of Å.

To account for the observation of a single diastereomer from the *cis*-configured substrates amide **3** and acid **2**, DFT modelling was performed on the amide complex **5** (Fig. 4). Complexes **5a** and **5b** were identified as the lowest-energy conformers for **5** (Scheme 2) and the diastereomer of **5** respectively.^21^ A key interaction common to both structures is a hydrogen bond between the chlorine and the hydrogen atoms of the amide moiety.^4^,^22^ Additionally, two CH/π interactions between the naphthyl groups of both ligands are apparent in both structures, with H-arene distances within the purview of what has been observed experimentally and computationally for this type of interaction.^23^ A structural feature distinguishing between the two diastereomers is the positioning of the dimethyl amino group that is located in **5b** under and in close proximity to the cyclobutene ring. This repulsive contact is imposed by the combined hydrogen bond and CH/π interactions. The contact is not as repulsive in **5a**, as judged from a greater separation, and thus appears to be responsible for the 2.1 kcal mol^–1^ preference for **5a**.

**Scheme 5:**
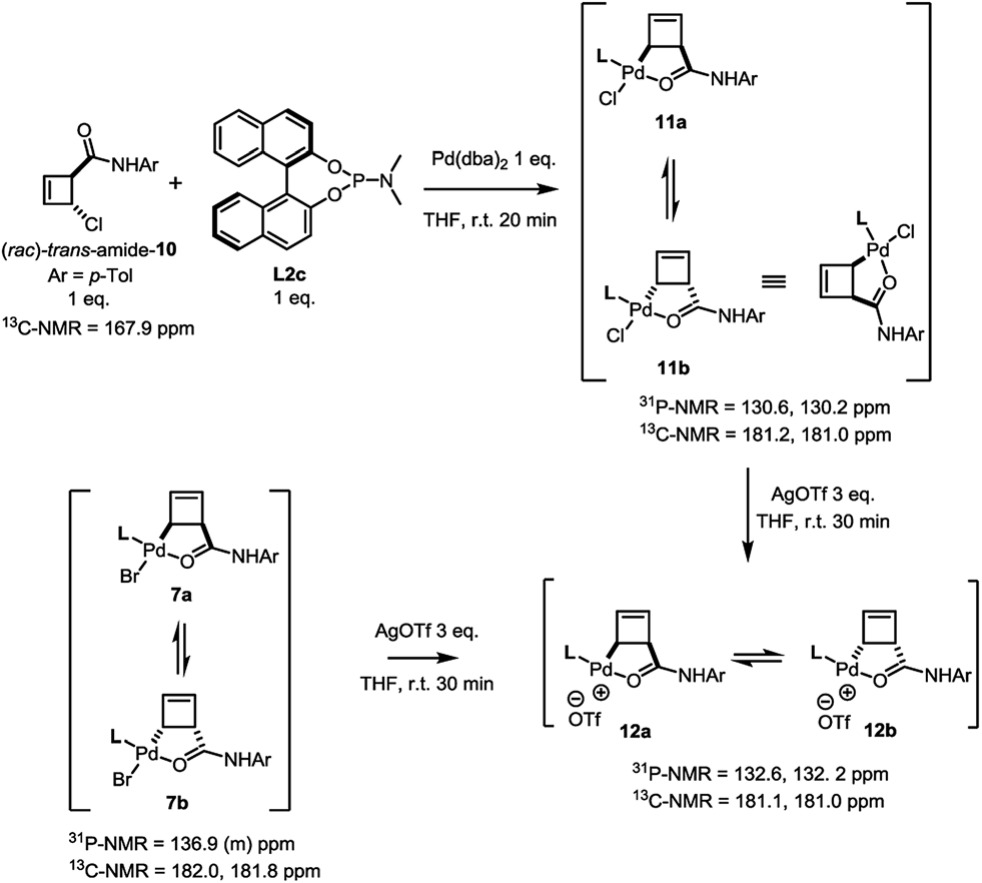
Demonstration of homology between halide complexes **7a/b** and **11a/b** through reaction with AgOTf.

In contrast to this and as in the case of the bromoamide **6**, subjection of the (*rac*)-*trans*-chloroamide **10** to the action of stoichiometric amounts of Pd(dba)_2_ and ligand **L2c** led to the formation of two η^1^-allylpalladium complexes in 6 : 5 ratio (Scheme 5). The spectral signature of this mixture is very similar to that of **7a/b**, supporting its analogous assignment as an internally coordinated, *syn*-species bearing a single phosphorus ligand. Simple demonstration of this analogy was achieved by exposing both the bromo-**7a/b** and the chloro-**11a/b** complexes to the action of silver triflate. Following filtration of the corresponding silver halide, an identical mixture of diastereoisomeric cationic palladium(ii) complexes **12** (7 : 5) was observed in solution.

**Scheme 6:**
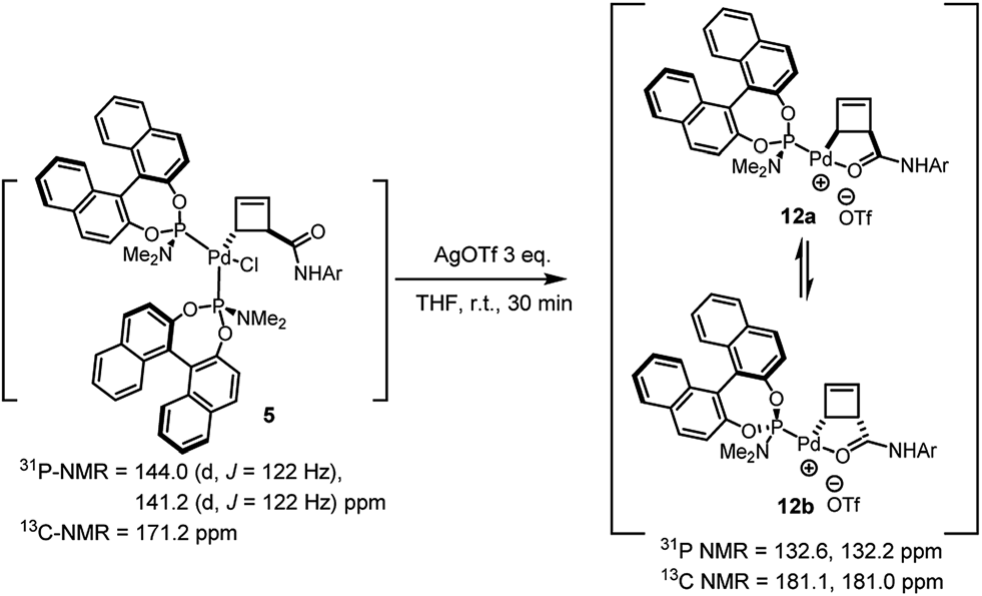
Reaction of *anti*-complex **5** with AgOTf.

Treatment of the diastereomerically pure anti-palladium complex **5** with silver triflate also led to precipitation of silver chloride and formation of a new organometallic species (Scheme 6). Much to our surprise, this was exactly the same mixture of *syn*-, internally chelated diastereoisomeric palladium complexes **12** that had been obtained by halide abstraction from the *syn*-complexes **7** and **11**! This unexpected result suggests the existence of a facile pathway for facial exchange of palladium within the cyclobutene framework. That this type of facial exchange could be triggered by ligand removal from the coordination sphere is, to the best of our knowledge, unprecedented.

Mechanisms for such a process have been proposed in the literature and typically involve bimolecular metal displacement.^24^ Further studies were conducted to shed light on this reaction (**5** → **12a/b**, Table 1), at first focusing on the concentration of reactants. A qualitatively striking change in the time required to reach full conversion to **12a/b** (from 45 min to 6 h) was observed, when the concentration of *anti*-complex **5** was lowered from 7.1 × 10^–2^ M to 1.4 × 10^–2^ M in THF-*d*_8_.^6^ Conversely, the addition of Pd(0) in the form of Pd(dba)_2_ accelerated the entire process, leading to full conversion in less than 5 min. Furthermore, the inhibition of conversion by addition of free ligand **L2c** suggests that a bimolecular process, which critically relies on the metal coordination sphere and is not promoted by nucleophilic displacement by a phosphorus centre, could be operative.^20^ It moreover becomes apparent that the thermodynamic value of internal coordination is remarkably high in these systems.

**Table 1:** The conversion time and concentration of *anti*-complex **5** and additives

Concentration (10^–2^ M)	Additive	Conversion time (min)
7.1	AgOTf, 3.0 eq.	45
1.4	AgOTf, 3.0 eq.	360
1.4	Pd(dba)_2_, 4.0 eq. and AgOTf, 3.0 eq.	<5
1.4	**L2c**, 8.0 eq. and AgOTf, 3.0 eq.	—^*a*^
1.4	AgOTf, 11 eq.	20
1.4	AgBF_4_, 8.0 eq. and AgOTf, 3.0 eq.	10

^*a*^No facial exchange observed. Complete decomposition in 250 min.

We thus returned to X-ray and NMR measurements of complex **7** in search of indications for aggregation to support the proposed bimolecular process. Standing at 2.575 Å, the distance between the bromine atom of one molecule in the unit cell and the N–H moiety of the next molecule is shorter than expected and suggestive of an *inter*molecular H-bond.^6^ However this might also be explained by packing effects in the solid state, which is why we investigated self-diffusion coefficients and the concentration dependence of chemical shifts in solution state NMR spectroscopy. Unfortunately, no conclusive results concerning aggregation could be obtained from the diffusion ordered spectroscopy (DOSY) spectrum of complex **7a/b** in THF solution (data not shown). However, both ^1^H and ^31^P resonances show pronounced differences in 0.1 M and 0.01 M solution, clearly pointing towards aggregation playing a role in solution.^6^,^25^

## Conclusion

In summary, we have identified structural features of novel internally coordinated, monohaptoallylpalladium(ii) species and directly investigated their dynamic behaviour in solution. The rare possibility to observe the two limiting η^1^-allylpalladium intermediates of an asymmetric allylic alkylation process allowed us to propose a mechanism for their interconversion (based on an η^1^ → η^3^ → η^1^ isomerisation) and to obtain support by combined DFT and NMR studies (in solution) and X-ray analysis. The interplay between structure and reactivity of these species as well as the direct observation of their unusually facile isomerisation behaviour should be of direct relevance to chemistries beyond catalytic allylic alkylation, given the current prominence of chelation-directed catalytic C–H activation methodologies.

## Supplementary Material

Supplementary information

Crystal structure data

## References

[cit1] (a) Negishi E., Handbook of Organopalladium Chemistry for Organic Synthesis, Wiley, New York, 2002.

[cit2] Giri R., Shi B.-F., Engle K. M., Maugel N., Yu J.-Q. (2009). Chem. Soc. Rev..

[cit3] Luparia M., Oliveira M. T., Audisio D., Frébault F., Goddard R., Maulide N. (2011). Angew. Chem., Int. Ed..

[cit4] Audisio D., Gopakumar G., Xie L.-G., Alves L. G., Wirtz C., Martins A. M., Thiel W., Farès C., Maulide N. (2013). Angew. Chem., Int. Ed..

[cit5] Souris C., Frébault F., Patel A., Audisio D., Houk K. N., Maulide N. (2013). Org. Lett..

[cit6] See ESI for further details.

[cit7] Trost B. M., van Vranken D. L. (1996). Chem. Rev..

[cit8] CCDC 991087 contains the supplementary crystallographic information for this paper.

[cit9] Tran L. D., Daugulis O. (2012). Angew. Chem., Int. Ed..

[cit10] Virtually all the crystalline samples picked up from different crops were consistent with isomer **7a** only. This implies that, in the crystalline phase, the complex **7** is predominantly comprised of isomer **7a**

[cit11] Böttcher B., Schmidts V., Raskatov J. A., Thiele C. M. (2010). Angew. Chem., Int. Ed..

[cit12] (a) Böttcher B. and Thiele C. M., in eMagRes, John Wiley & Sons, Ltd, 2012.

[cit13] The details for the preparation of the PDMS sticks used will be the subject of a further report. For seminal work on the use of cross-linked PDMS for RDC measurements, see: Freudenberger J. C. Spiteller P. Bauer R. Kessler H. Luy B., J. Am. Chem. Soc., 2004, 126 , 14690 –14691 . 15535672 10.1021/ja046155e

[cit14] Thiele C. M., Bermel W. (2012). J. Magn. Reson..

[cit15] Neese F. (2012). Wiley Interdiscip. Rev.: Comput. Mol. Sci..

[cit16] (b) Schmidts V., PhD thesis, Technische Universität Darmstadt, Darmstadt, Germany, 2013.

[cit17] Thiele C. M., Petzold K., Schleucher J. (2009). Chem.–Eur. J..

[cit18] Brüschweiler R., Zhang F. (2004). J. Chem. Phys..

[cit19] Johansson C., Lloyd-Jones G. C., Norrby P.-O. (2010). Tetrahedron: Asymmetry.

[cit20] A dissociative pathway involving phosphine dissociation prior to isomerization was also considered on account of the potential assistance by the amide carbonyl. This pathway is computed to be considerably higher in energy (Δ*G*≠diss = 32.4 kcal mol^–1^) and is thus included in the ESI.

[cit21] See the ESI for a discussion of results for additional conformers with and without dispersion corrections

[cit22] Aullón A., Bellamy D., Brammer L., Bruton E. A., Orpen A. G. (1998). Chem. Commun..

[cit23] Hartmann E., Gschwind R. M. (2013). Angew. Chem., Int. Ed..

[cit24] Granberg K. L., Bäckvall J.-E. (1992). J. Am. Chem. Soc..

[cit25] Mitra A., Seaton P. J., Assarpour R. A., Williamson T. (1998). Tetrahedron.

