# Supplementary material for: Dynamic behaviour of monohaptoallylpalladium species: internal coordination as a driving force in allylic alkylation chemistry
Source: Chem Sci. 2015 Jul 6;6(10):5734–9. doi: 10.1039/c5sc01867f (PMC5975841; doi:10.1039/c5sc01867f)
Supplement: Supplementary file 1 [file SC-006-C5SC01867F-s001.pdf]

Electronic Supplementary Material (ESI) for Chemical Science.
This journal is © The Royal Society of Chemistry 2015

Supporting Information

Supporting Information
Experimental Part

Dynamic behavior of monohaptoallylpalladium species: internal coordination as a driving force in allylic alkylation chemistry

Lan-Gui Xie,[‡,a] Viktor Bagutski,[‡,b] Davide Audisio,[c] Larry Wolf,[c] Volker Schmidts,[b] Kathrin Hofmann,[d] Cornelia Wirtz,[c] Walter Thiel,[c] Christina M. Thiele*[,b] and Nuno Maulide*[,a]

[a] University of Vienna, Faculty of Chemistry, Institute of Organic Chemistry, Währinger Strasse 38, 1090 Vienna, Austria
[b] Technische Universität Darmstadt, Clemens Schöpf Institut für Organische Chemie und Biochemie, Alarich-Weiss-Str. 16, 64287 Darmstadt, Germany
[c] Max-Planck-Institut für Kohlenforschung, Kaiser-Wilhelm-Platz 1, 45470 Mülheim an der Ruhr, Germany
[d] Technische Universität Darmstadt, Eduard-Zintl-Institute, Alarich-Weiss-Str. 12, 64287 Darmstadt, Germany

nuno.maulide@univie.ac.at

cthiele@thielelab.de

Contents

1. General information 3
2. Preparation of the starting materials 3
3. Synthesis of intermediate 4 5
4. Synthesis of intermediate 5 6
5. Preparation of (*R, 1S, 4S*)-Pd-Complex 7a/b 7
6. Synthesis of intermediate 8 12
7. Synthesis of intermediate 11a/b 12
8. Synthesis of intermediate 12a/b 14
9. Reaction of 7a/b and 12a/b with sodium malonate 20
10. Enrichment of the diastereomeric mixture 7a/7b with isomer 7a 21
11. X-Ray Crystallography of 7a 22
12. Dynamics studies by selective 1D PFGSE NOE Spectra 23
13. Calculation of Structure Models for 7a/b by DFT 26
14. Validation of Structure Models for 7a/b using Residual Dipolar Couplings 30
15. Investigation of the Aggregation Behavior 34
16. Facial exchange of complex 5 in presence of AgOTf and additives 36
References 36
NMR and HPLC 37

1. General information

All reactions were carried out in flame-dried glassware under an atmosphere of argon. All solvents were distilled from appropriate drying agents prior to use. All reagents were used as received from commercial suppliers unless otherwise stated. Neat infra-red spectra were recorded using a Perkin-Elmer Spectrum 100 FT-IR spectrometer. Wavelengths (ν) are reported in cm^{-1}. Mass spectra were obtained using a Finnigan MAT 8200 (70 eV) or an Agilent 5973 (70 eV) spectrometer, using electrospray ionization (ESI). Accurate mass determinations were obtained on a Bruker APEX III FT-MS (7 T magnet). All ^{1}H-NMR and ^{13}C-NMR experiments were recorded using Bruker AV-400, AV-500 and AV-600 spectrometers. Chemical shifts (δ) are quoted in ppm and coupling constants (*J*) are quoted in Hz. Reaction progress was monitored by thin layer chromatography (TLC) performed on aluminum plates coated with kieselgel F254 with 0.2 mm thickness. Visualization was achieved by a combination of ultraviolet light (254 nm) and acidic potassium permanganate or anisaldehyde. Flash column chromatography was performed using silica gel 60 (230-400 mesh, Merck and co.). Bis(dibenzylideneacetone)palladium(0) $Pd(dba)_2$ was purchased from Sigma-Aldrich. **L2a** and (*R*)-MonoPhos **L2c** were purchased from Sigma-Aldrich or prepared according to the procedure described in the literature.[1] (*rac*)-**2**[2] was prepared according to the procedure described in the literature.

2. Preparation of the starting materials

(rac)-cis-amide 3 and *(rac)-trans*-amide 10

To a cold (0 °C) stirred solution of a crude mixture of *cis*-**2** and *trans*-**2** isomers (6:1 ratio *cis*-**2** / *trans*-**2**, 1.50 mmol, 1.0 equiv.) in dry DCM (15 mL), under Argon, EDCI (315 mg, 1.65 mmol, 1.1 equiv.), HOBt (222 mg, 1.65 mmol, 1.1 equiv.) and *p*-toluidine (178 mg, 1.65 mmol, 1.1 equiv.) were added in this order and the resulting mixture was stirred at room temperature. After 14h, an aqueous solution of $NaHCO_3$ (30 mL) was added and the mixture was extracted successively with DCM (10 mL x 3 times). The combined organic phases were dried (Na_2SO_4) and concentrated under reduced pressure. ^{1}H-NMR analysis of the crude mixture showed that the ratio between *trans*-amide **10** and *cis*-amide **3** was 1/4. This crude material was carefully

[1] M. R. Krout, J. T. Mohr, B. M. Stoltz *Org. Synth.* **2009**, *86*, 181-193.
[2] D. Audisio, M. Luparia, M. T. Oliveira, D. Klütt, N. Maulide, *Angew. Chem. Int. Ed.* **2012**, *51*, 7314-7317.

separated by flash column chromatography on silica gel (*n*-pentane/ EtOAc, 8/2 to 6/4) to afford *trans*-amide **10** (43 mg, 0.196 mmol, 13 %) as yellow solid, followed by the more polar *cis*-amide **3** (172 mg, 0.777 mmol, 52 %) as a yellow solid.

***(rac)-cis*-amide 3**

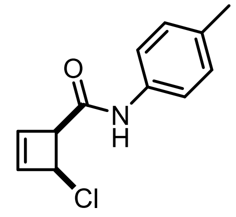

Yellow solid;
IR (neat) ν_{max}: 3296, 3262, 3133, 3082, 1661, 1608, 1543, 1512, 1405, 1356, 1304, 1282, 1250, 1188, 1132, 999, 902, 813, 774, 746.
^{1}H-NMR (500 MHz, $CDCl_3$): δ 7.51 (bs, 1H, CONH), 7.42 (d, J = 8.4, 2H, H_{arom}), 7.12 (d, J = 8.4 Hz, 2H, H_{arom}), 6.42 (d, J = 2.6 Hz, 1H, H_{cB}), 6.38 (m, 1H, H_{cB}), 5.12 (d, J = 4.3 Hz, 1H, H_{cB}), 4.10 (d, J = 4.3 Hz, 1H, H_{cB}), 2.30 (s, 3H, H_{tol}).
^{13}C-NMR (125 MHz, $CDCl_3$): δ 167.4, 142.8, 136.5, 134.8, 134.4, 129.5 (2C), 120.5 (2C), 56.8, 56.6, 20.9.
HRMS (ESI+): exact mass calculated for $[M+Na]^+$ ($C_{12}H_{12}ClNNaO$) requires *m*/*z* 244.0500, found *m*/*z* 244.0501**.**

***(rac)-trans*-amide 10**

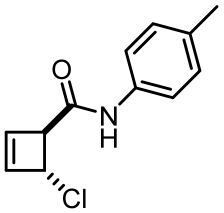

Yellow solid;
IR (neat) ν_{max}: 3256, 3033, 2921, 1644, 1602, 1533, 1511, 1406, 1357, 1282, 1250, 1220, 1190, 990, 780, 713.
^{1}H-NMR (500 MHz, $CDCl_3$): δ 7.76 (bs, 1H, CONH), 7.39 (d, J = 8.5 Hz, 2H, H_{arom}), 7.11 (d, J = 8.5 Hz, 2H, H_{arom}), 6.34 (m, 1H, H_{cB}), 6.30 (m, 1H, H_{cB}), 4.99 (s, 1H, H_{cB}), 3.73 (s, 1H, H_{cB}), 2.31 (s, 3H, H_{tol}).
^{13}C-NMR (125 MHz, $CDCl_3$): δ 167.9, 141.4, 136.4, 134.8, 134.5, 129.5 (2C), 120.3 (2C), 59.8, 56.6, 20.9.
HRMS (ESI+): exact mass calculated for $[M+Na]^+$ ($C_{12}H_{12}ClNNaO$) requires *m*/*z* 244.0500, found *m*/*z* 244.0501.

***(rac)-trans*-amide 6**

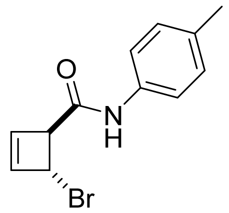

To a cold (0°C) stirred solution of a crude mixture of cis and trans isomers of 4-bromo-cyclobut-2-ene-1-carboxylic acid. (1:10 ratio *cis* / *trans*, 500 mg, 2.82 mmol, 1.0 equiv.) in dry DCM (20 mL), under Argon, EDCI (592 mg, 3.10 mmol, 1.1 equiv.), HOBt (419 mg, 3.10 mmol, 1.1 equiv.) and *p*-toluidine (332 mg, 3.10 mmol, 1.1 equiv.) were added in this order and the resulting mixture was stirred at room temperature. After 22h, an aqueous solution of $NaHCO_3$ (30 mL) was added and the mixture was extracted successively with DCM (10 mL x 3 times). The combined organic phases were dried (Na_2SO_4) and concentrated under reduced pressure. ^{1}H-NMR analysis of the crude mixture showed that the ratio between the resulting *trans* and *cis* amide was > 10/1. This crude material was purified by flash column chromatography on silica gel (*n*-pentane/EtOAc, 8/2) to afford pure *trans*-amide **6** (388 mg, 1.46 mmol, 52 %) as yellow solid.

IR (neat) ν_{max}: 3246, 3035, 2917. 1641, 1593, 1529, 1513, 1406, 1348, 1283, 1249, 1183, 989, 825, 797, 759, 741, 700.

^{1}H-NMR (500 MHz, THF-d_8): δ 9.13 (bs, 1H, CONH), 7.49 (d, J = 8.5 Hz, 2H, H_{arom}), 7.04 (d, J = 8.5 Hz, 2H, H_{arom}), 6.35 (m, 1H, H_{cB}), 6.30 (m, 1H, H_{cB}), 5.12 (s, 1H, H_{cB}), 3.81 (d, J = 0.8 Hz, 1H, H_{cB}), 2.25 (s, 3H, H_{tol}).

^{13}C-NMR (125 MHz, THF-d_8): δ 167.6, 142.0, 137.9, 137.5, 133.3, 129.6 (2C), 119.8 (2C), 61.2, 46.6, 20.7.

HRMS (ESI+): exact mass calculated for $[M+Na]^+$ ($C_{12}H_{12}BrNNaO$) requires m/z 287.9994, found m/z 287.9999.

3. Synthesis of intermediate 4

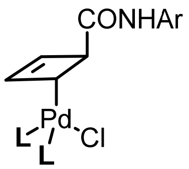

In a flame dried schlenk flask, under Argon atmosphere, Pd(dba)$_2$ (28 mg, 0.048 mmol, 1 equiv.), **L2a** (51.8 mg, 0.096 mmol, 2 equiv.) and *cis*-amide **3** (11 mg, 0.048 mmol, 1 equiv.) were added. After three vacuum-Argon cycles, 1.0 mL THF-d_8 was added. The solution was stirred at room temperature for 20 minutes (the color of the mixture slowly changes from purple to yellow). The mixture is then transferred to a schlenk NMR tube, sealed and analyzed at 278 K.

The compound underwent decomposition preventing full assignment.

HRMS (ESI+): exact mass calculated for [M] ($C_{34}H_{30}ClN_2O_3PPd$) requires m/z 1405.3671, found m/z 1405.3701.

^{31}P-NMR (203 MHz, THF-d_8): δ 144.8 (d, J = 98.9 Hz), 138.0 (d, J = 98.9 Hz).

4. Synthesis of intermediate 5

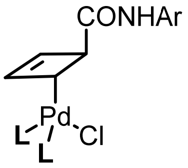

L = (*R*)-MonoPhos

In a flame dried schlenk flask, under Argon atmosphere, Pd(dba)$_2$ (28 mg, 0.048 mmol, 1 equiv.), (*R*)-Monophos (35 mg, 0.096 mmol, 2 equiv.) and *cis*-amide **3** (11 mg, 0.048 mmol, 1 equiv.) were added. After three vacuum-Argon cycles, 1.0 mL THF-d_8 was added. The solution was stirred at room temperature for 20 minutes (the color of the mixture slowly changes from purple to yellow). The mixture is then transferred to a schlenk NMR tube, sealed and analyzed at 278 K.

^{1}H-NMR (500 MHz, THF-d_8): δ 10.52 (s, 1H, CONH), 8.40 (d, J = 8.6 Hz, 1H), 8.18 (d, J = 8.6 Hz, 1H), 8.14-8.08 (m, 4H$_{arom}$), 8.04-8.00 (m, 3H$_{arom}$), 7.84-7.70 (m, dba + H$_{arom}$), 7.63-7.59 (m, H$_{arom}$), 7.48-7.24 (m, dba + H$_{arom}$), 7.07 (d, J = 8.0 Hz, 2H), 6.68 (s, 1H, H$_{cB}$), 6.00 (s, 1H, H$_{cB}$), 4.41 (d, $J_{P\text{-}H}$ = 4.7 Hz, H$_{cB}$), 4.16 (d, $J_{P\text{-}H}$ = 9.6 Hz, H$_{cB}$), 2.29 (s, 3H), 2.26 (s, 3H), 2.24 (s, 3H), 1.96 (s, 3H), 1.94 (s, 3H).

^{13}C-NMR (125 MHz, THF-d_8): δ 188.2 (dba), 171.2, 151.8, 150.4, 150.3, 150.0, 149.9 (2C), 148.9 (2C), 147.7 (dd, $J_{P\text{-}C}$ = 10.3, 5.8 Hz), 143.1 (dba), 139.0, 136.2 (dba), 135.6, 133.3, 133.2, 133.1 (2C), 132.7, 132.6, 132.3, 132.0, 131.9, 131.7, 131.4, 131.0 (dba), 130.3, 130.0, 129.7 (dba), 129.6 (2C), 129.5, 129.3, 129.2 (dba), 128.6, 127.7, 127.6 (2C), 127.4, 127.3, 127.1, 126.8, 126.5, 126.5 (dba), 126.4, 126.2, 126.1, 125.5, 124.3, 124.2, 123.4 (2C), 123.3, 122.2, 122.1, 121.9, 119.5, 118.9, 114.8, 58.2 (dd, $J_{P\text{-}C}$ = 8.8, 2.8 Hz), 53.9 (dd, $J_{P\text{-}C}$ = 152.0, 11.7 Hz), 37.2, 37.1, 37.0, 36.9, 21.0.

^{31}P-NMR (122 MHz, THF-d_8): δ 144.0 (d, J = 122 Hz), 141.2 (d, J = 122 Hz).

Relevant chemical shifts and coupling constants are listed in the table:

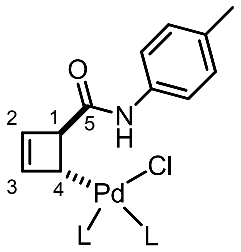

	^{1}H (ppm)	^{13}C(ppm)

1	4.41 (d, $J_{P\text{-}H}$ = 4.7 Hz)	58.2 (dd, $J_{P\text{-}C}$ = 8.8, 2.8 Hz)
2	6.00 (s)	130.3
3	6.68 (s)	147.7 (dd, $J_{P\text{-}C}$ = 10.3, 5.8 Hz)
4	4.16 (d, $J_{P\text{-}H}$ = 9.6 Hz)	53.9 (dd, $J_{P\text{-}C}$ = 152.0, 11.7 Hz)
5	-	171.2

5. Preparation of (*R, 1S, 4S*)-Pd-Complex 7a/b

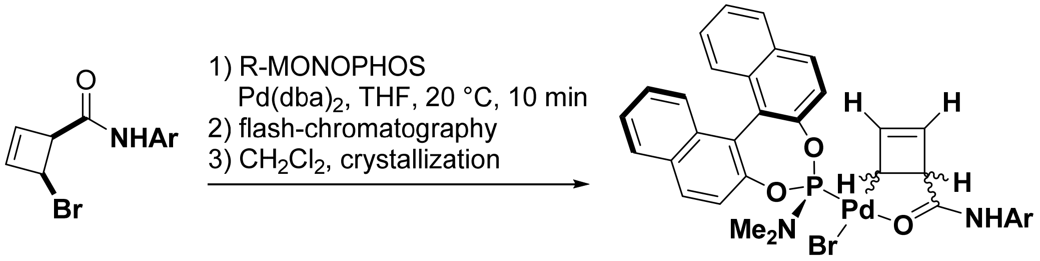

A Schlenk tube was charged in a glove-box with Teflon-coated stirring bar, Pd(dba)$_2$ (Aldrich, 30.0 mg, 52.2 μmol), MONOPHOS-ligand (**L2c**) (18.8 mg, 52.3 μmol), 4-bromocyclobut-2-enecarboxylic acid *para*-toluamide **6** (14 mg, 52.6 μmol), and connected to an argon-vacuum line. The solids were dissolved in degassed THF (2 mL) at ambient temperature and the reaction mixture was stirred for additional 30 min (usually, the reaction is accomplished after 5 min as indicated by color change from dark-red to pale green and precipitation of amounts of Pd-black). The solvent was then removed under reduced pressure at ambient temperature, the residue was re-dissolved in minimal amount of degassed CH_2Cl_2 (1–1.5 mL) and applied under argon onto plug of silica gel (2 cm, Ø15 mm). Elution with degassed CH_2Cl_2 (10 mL) followed by EtOH-DCM-mixture with polarity gradient from 2% to 3% of EtOH within the four following fractions (3 mL each), afforded after the solvents removal a crude product as yellow solid. This was dissolved/suspended in 1–1.5 mL of DCM, transferred into a weighed NMR-tube and left at 4 °C for crystallization. After the crystallization was accomplished, the supernatant solution was accurately drained off, the residue was washed with DCM (2×0.5 mL) and dried in high vacuum (<10^{-2} mbar) at ambient temperature over 2 h to give 25 mg (66%) of pure product as a colorless solid.

IR (neat) ν_{max}: 3244, 3187, 3059, 2921, 1615, 1586, 1549, 1511, 1462, 1324, 1302, 1225, 1202, 1178, 1069, 990, 948, 823, 806, 749, 724, 698, 686.

^{1}H-NMR (600 MHz, THF-d_8, 300K): δ 2.28 (s, 3H), 2.78 (s, 3H, NCH$_3$-1$_a$), 2.80 (s, 3H, NCH$_3$-2$_a$), 2.81 (s, 3H, NCH$_3$-1$_b$), 2.83 (s, 3H, NCH$_3$-2$_b$), 3.58 (m, 1H, *c*B-4$_b$, 3.90 (dd, *J* 6.4, 2.6 Hz, 1H, *c*B-4$_a$), 3.95 (d, *J* 2.6 Hz, 1H, *c*B-1$_b$), 3.99 (d, *J* 2.6 Hz, 1H, *c*B-1$_a$), 4.94 (d, *J* 1.8 Hz, 1 H, *c*B-3$_a$), 5.42 (m, 1H, *c*B-2$_a$), 5.86 (m, 1H, *c*B-2$_b$), 6.67 (d, *J* 1.8 Hz, 1 H, *c*B-3$_b$), 7.09-7.12 (m, 4H, *m*Tol$_{ab}$), 7.22-7.33 (m, 6H), 7.40-7.49 (m, 8H), 7.52-7.55 (m, 4H, *o*Tol$_{ab}$), 7.96-8.01 (m, 4H), 8.03 (d, *J* 8.8 Hz, 2H, 4-BNc$_{ab}$), 8.07 (d, *J* 8.8 Hz, 1H, 4-BNn$_b$), 8.12 (d, *J* 8.8 Hz, 1H, 4-BNn$_a$), 8.32 (d, *J* 8.8 Hz, 1H, 3-BNn$_a$), 8.33 (d, *J* 8.8 Hz, 1H, 3-BNn$_b$), 9.96 (s, 1H, N*H*$_a$), 9.98 (s, 1H, N*H*$_a$) ppm.

^{13}C-NMR (151 MHz, THF-d_8, 300K): δ 20.79 (2CH$_3$), 38.45 (NCH$_3$-1$_a$), 38.52 (NCH$_3$-2$_a$,1$_b$), 38.59 (NCH$_3$-2$_b$), 49.01 (d, *J* 13.2 Hz, *c*B-4$_a$), 50.25 (d, *J* 14.3 Hz, *c*B-4$_b$), 61.27 (d, *J* 2.7 Hz, *c*B-1$_a$), 61.63 (d, *J* 2.7 Hz, *c*B-1$_b$), 120.62 (2CH, *o*Tol), 120.64 (2CH, *o*Tol), 121.98 (CH, 3-BNc$_a$), 122.04 (CH, 3-BNc$_b$), 122.94, 123.03, 123.45 (d), 123.58 (d), 124.15 (CH, 3-BNn$_b$), 124.35 (CH, 3-BNn$_a$), 125.73, 125.82, 125.88, 126.45, 126.50, 126.61, 126.74, 127.16, 127.19, 127.39, 127.63, 128.73, 128.84, 129.08, 129.33, 129.38, 129.96 (4CH, *m*Tol), 131.13, 131.16 (CH, 4-BNn$_b$), 131.18, 131.32 (CH, 4-BNn$_a$), 132.15, 132.66, 132.92, 133.0, 133.21, 134.94 (*C*-Me), 134.96 (*C*-Me), 136.07 (NH-*C*), 149.47, 149.51, 149.54, 149.57, 150.36, 150,39, 150.46, 150.48, 150.61 (*c*B-3$_a$), 151.21 (*c*B-3$_b$), 181.78 (CONH$_b$), 181.95 (CONH$_a$) ppm.

^{31}P (162 MHz, THF-d_8, 300K): δ= 136.9 (m) ppm.

HRMS (ESI+): exact mass calculated for $[M-Br]^+$ ($C_{34}H_{30}N_2O_3PPd$) requires *m*/*z* 651.1022, found *m*/*z* 651.1039.

Relevant chemical shifts and coupling constants are listed in the table:

All measurements were recorded at 300 K without sample spinning on a Bruker Avance III 600 MHz spectrometer equipped with a triple resonance broadband inverse probe (1H/31P/2H-BB, *z*-gradient), unless noted otherwise. A BCU-Xtreme was used for temperature control. Acquisition and processing of NMR data was performed in TopSpin 3.1 (pl2).

Standard pulse programs from the Bruker library were used for assignment of resonances: 1D ^{1}H and ^{13}C, ^{1}H-^{1}H COSY, ^{1}H-^{13}C HSQC (each with and without ^{31}P decoupling), ^{1}H-^{13}C HMBC, and ^{1}H-^{1}H EASY-ROESY[1] (6.5 kHz spin lock, 45° tilt angle, 300 ms mixing). T_1 relaxation time constants were determined by the standard inversion recovery method. Quantitative NOE/EXSY spectra were acquired with the *selnogpzs* pulse program (see below).

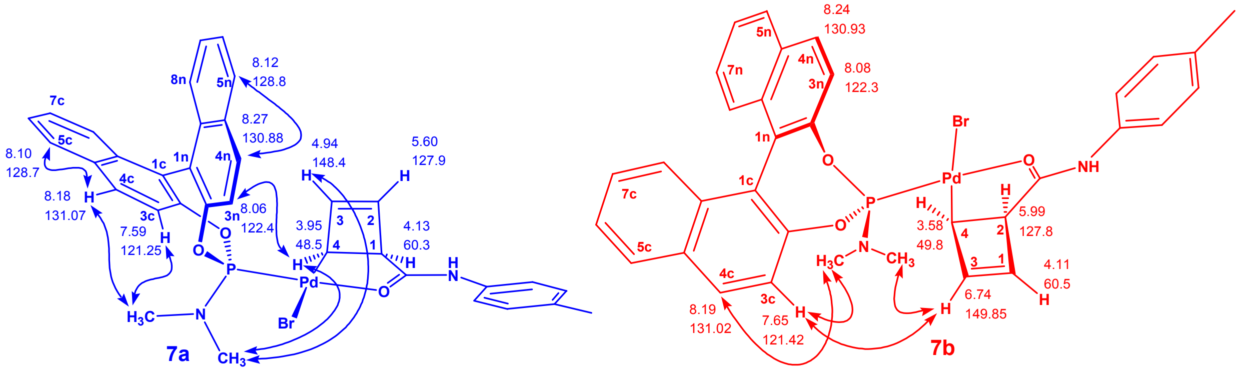

Figure SI-1: Atom numbering used for assignment and NOE analysis. Descriptors "*a*" or "*b*" refer to the corresponding isomers **7a** or **7b**. Naphthyl ring coplanar to cyclobutene is marked with "*c*"-suffix, normal - with "*n*".

Table SI-1: Chemical shifts and assignments of ^{1}H and ^{13}C spectra recorded in DMSO-*d6* at 300K, including observed COSY, ROESY and HMBC correlations as well as ^{1}H T_1 relaxation times (M=unresolved or only partially resolved multiplet comprising several signals, blue color code: signals belonging to **7a**, red: **7b**; black: non distinguishable/only one signal set observed).

Assign.	^{1}H ($J_{(C,H)}$)	^{13}C ($J_{(C,P)}$)	HH-COSY	HMBC ($J_{(C,P)}$)	ROESY	T_1, s
CH_3	2.27	20.5	7.19 (^{4}J)<br>7.47 (^{5}J)	129.4 (*m*Tol)<br>134.3 (C_{quat})	*m*Tol	1.786
NMe-1_a	2.71	37.62	-	-	3-a, 4-a<br>7.59 (3'c)<br>8.18	1.545
NMe-1_b	2.72	37.64	-	-	3-b, 4-b<br>7.65 (3'c)	=
NMe-2_a	2.73	37.69	-	-	=	=
NMe-2_b	2.74	37.71	-	-	=	=
*c*B-4_b	3.58 (m)	49.8 (13 Hz)	6.74 (3-b)<br>5.99 (2-b)<br>4.11 (1-b)	127.8 (2-b, 9 Hz)<br>149.8 (3-b)	(1-b, 3-b)	3.510
*c*B-4_a	3.95 (m)	48.5 (13 Hz)	5.60 (3-a)<br>4.92 (2-a)<br>4.13 (1-a)	127.9 (2-a, 8 Hz)<br>148.4 (3-a)	NMe, 1-a,<br>3-a	2.920
*c*B-1_b	4.11 (2.6)	60.5	6.74 (3-b)<br>5.99 (2-b)<br>3.58 (4-b)	127.8 (2-b, 9 Hz)<br>149.8 (3-b)<br>180.5	2-b, 4-b	2.331

*c*B-1_a	4.13 (2.6)	60.3	5.60 (3-a<br>4.92 (2-a)<br>3.95 (4-a)	127.9 (2-a, 8 Hz)<br>148.4 (3-a)<br>180.6	2-a, 4-a	2.193
*c*B-3_a	4.94	148.4	4.92 (2-a)<br>4.13 (1-a)<br>3.95 (4-a)	60.3 (1-a)<br>48.5 (4-a)-NOT observed	2-a, 4-a	4.450
*c*B-2_a	5.60	127.9	5.60 (3-a<br>4.13 (1-a)<br>3.95 (4-a)	48.5 (4-a)<br>60.3 (1-a)	1-a, 3-a<br>*o*Tol	4.272
*c*B-2_b	5.99	127.8	6.74 (3-b)<br>4.11 (1-b)<br>3.58 (4-b)	49.8 (4-b)<br>60.5 (1-b)	NMe, 1-b,<br>3-b	4.179
*c*B-3_b	6.74 (1.8)	149.85	5.99 (2-b)<br>4.11 (1-b)<br>3.58 (4-b)	49.8 (4-b)<br>60.5 (1-b)<br>127.8 (2-b, 9 Hz)	NMe, 2-b,<br>4-b	3.633
*m*Tol	7.19	129.3	2.27 (CH_3)<br>7.47 (*o*Tol)	20.5 (CH_3)<br>120.1 (*o*Tol, vw)<br>134.7 (*C*-NHCO-)	CH_3	3.180
8-BN_b	7.23 (8.6)	126.1	M1	125.6<br>131.29 (4a-BN_b*)	M1	2.910
8-BN_a	7.25 (8.6)	125.9	M1	122.23<br>126.76<br>131.21 (4a-BN_b)	M1	2.787
8-BN_a	7.28 (8.6)	126.0	7.35-7.41	130.86 (4a-BN_b)	M1	3.260
8-BN_b	7.29 (8.6)	=	7.35-7.41	130.91 (4a-BN_b*)	M1	=
M1, 2H<br>7-BNn_{ab}<br>6-BNc_{ab}	7.35-7.41	 126.67<br>126.76<br>126.89<br>126.91	M2<br>7.22 (8.6)<br>7.24 (8.6)<br>7.28 (8.6)<br>7.29 (8.6)	131.51<br>131.55<br>131.60	8.11	2.747
*o*Tol	7.47	120.12<br>120.13	7.19	134.28 (*C*-CH_3)	*c*B-1_{ab},<br>CH_3 (vw)	3.049
M2, 2H	7.51-7.57	125.45	M1	126.04	8.05-8.13	2.730

6-BNn$_{ab}$<br>7-BNc$_{ab}$		125.47<br>125.57<br>125.65	8.10<br>8.12	126.66<br>131.21<br>131.29		
3-BNc$_a$	7.59 (8.8)	121.25	8.18 (8.9)	*130.80***	NMe<br>4-BNc$_a$	3.462
3-BNc$_b$	7.65 (8.8)	121.42	8.19 (8.9)	125.46 (8a-BNc)	NMe<br>4-BNc$_b$<br>*c*B-3$_b$	3.488
M3, 3H<br>3+3 d	8.05-8.13<br>8.06 (8.9)-3-BNn$_a$<br>8.08 (8.9)-3-BNn$_b$	 122.4<br>122.3	7.51-7.57<br>8.27 (8.9)<br>8.24 (8.8)	126.8<br>126.9	8.27,<br>*c*B-4$_a$, NMe<br>8.18<br>8.24	3.055
5-BNc$_a$	8.10	128.7-8			8.18	=
5-BNc$_b$	8.10	128.7-8			8.19	
5-BNn$_a$	8.12	128.7-8	7.54, "M2"		8.27 (4BNn$_a$)	=
5-BNn$_b$	8.12	128.7-8				
4-BNc$_a$	8.18 (8.9)	131.07	7.59 (8.8)	149.00(2)	NMe (vw)<br>3-BNc$_a$<br>8.10(5BNc)	3.111
4-BNc$_b$	8.19 (8.9)	131.02	7.65 (8.8)	148.91(3)	NMe (vw)<br>3-BNc$_b$<br>8.10	=
4-BNn$_b$	8.24 (8.8)	130.93	8.08	147.64(8) (d)	8.08 (8.9)	2.737
4-BNn$_a$	8.27 (8.9)	130.88	8.06	147.64(8) (d)	8.06<br>8.13	2.681
CONH$_a$	11.08	180.6	-	120.12 (*o*Tol)	-	0.985
CONH$_b$	11.11	180.5	-	=	-	1.003

NOT observed: cross-peak that was expected was not observed

* no distinction between coplanar and normal orientation possible

** most probably

6. Synthesis of intermediate 8

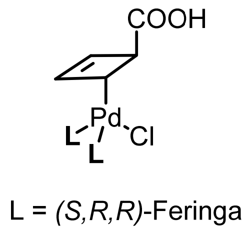

In a flame dried schlenk flask, under Argon atmosphere, Pd(dba)$_2$ (28 mg, 0.048 mmol, 1 equiv.), **L2a** (51.8 mg, 0.096 mmol, 2 equiv.) and *cis*-**2** (6.36 mg, 0.048 mmol, 1 equiv.) were added. After three vacuum-Argon cycles, 1.0 mL THF-d_8 was added. The solution was stirred at room temperature 20 minutes. The mixture is then transferred to a schlenk NMR tube, sealed and analyzed at 278 K.

The compound underwent decomposition preventing full assignment.

HRMS (ESI+): exact mass calculated for $[M\text{-}Cl]^+$ ($C_{34}H_{30}N_2O_3PPd$) requires m/z 1281.3353, found m/z 1281.3542.

^{31}P-NMR (203 MHz, THF-d_8): δ 144.9 (d, J = 99 Hz), 137.6 (d, J = 99 Hz).

7. Synthesis of intermediate 11a/b

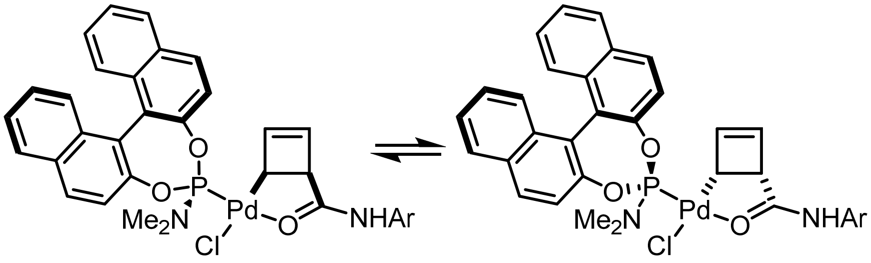

11a **11b**

In a schlenk flask (dry and under Argon atmosphere), Pd(dba)$_2$ (39 mg, 0.068 mmol, 1 equiv.), the (*R*)-MonoPhos ligand (24.4 mg, 0.068 mmol, 1 equiv.) and *trans*-amide **10** (15 mg, 0.068 mmol, 1 equiv.) were added. After three vacuum- Argon cycles, 3.0 mL THF was added. The solution was stirred at room temperature 20 minutes. The solvent was evaporated and the crude product was purified by flash chromatography on silica gel to give 26 mg (0.038 mmol, yield 56%) of a brown solid. [Column: diameter 2 cm; silica ca. 3 cm high. Eluent: DCM 100% (ca. 50 mL) to remove the dba, followed by DCM:EtOH 95:5 (ca. 50 mL)].

IR (neat) ν_{max} 3059, 2924, 1651, 1619, 1588, 1554, 1512, 1449, 1339, 1226, 1186, 1098, 1071, 1046, 992, 950, 826, 767, 752, 726, 699, 608.

^{1}H-NMR (400 MHz, THF-d_8): δ 10.6 (2 bs, 2H, CONH), 8.45 (t, J = 8.5 Hz, 2H, H$_{arom}$), 8.28 (d, J = 3.6 Hz, 2H, H$_{arom}$), 8.26 (d, J = 3.4 Hz, 2H, H$_{arom}$), 8.20 (m, 4H, H$_{arom}$), 7.82 (d, J = 8.5 Hz, 2H, H$_{arom}$), 7.79 (d, J = 8.8 Hz, 2H, H$_{arom}$), 7.73-7.61 (m, 8H, H$_{arom}$), 7.58-7.44 (m, 6H, H$_{arom}$),

7.22 (d, J = 8.3 Hz, 4H, H_{arom}), 6.84 (s, 1H, H_{cB}), 6.17 (s, 1H, H_{cB}), 5.72 (s, 1H, H_{cB}), 5.21 (s, 1H, H_{cB}), 4.42 (s, 1H, H_{cB}), 4.30 (s, 1H, H_{cB}), 4.05 (d, J = 5.6 Hz, 1H, H_{cB}), 3.76 (dm, J = 5.5 Hz, 1H, H_{cB}), 3.04 (s, 3H, N-CH_3), 3.01 (s, 3H, N-CH_3), 3.00 (s, 3H, N-CH_3), 2.97 (s, 3H, N-CH_3), 2.45 (s, 6H).

^{13}C-NMR (125 MHz, THF-d_8): δ 182.1, 182.0, 150.4, 150.3, 149.5, 149.4, 147.0, 143.3, 136.6, 136.4, 136.3, 135.3, 135.2, 133.6, 133.4, 133.1, 132.9, 132.7, 132.6, 131.9, 131.8, 131.6, 131.1, 130.3, 129.9, 129.8, 129.5, 129.3, 128.1 (d, J = 2.2 Hz, *c*B), 128.0 (d, J = 2.1 Hz, *c*B), 127.7, 127.6, 127.2, 127.1, 126.8, 126.3, 126.3, 126.2, 124.6, 124.5, 124.2, 124.0 (d), 123.8 (d), 123.4, 123.3, 122.4 (2C), 121.7, 121.4, 62.4 (d, J = 2.7 Hz, *c*B), 62.1 (d, J = 2.6 Hz, *c*B), 48.2 (d, J = 12.1 Hz, *c*B), 47.1 (d, J = 15.1 Hz, *c*B), 38.8, 38.7, 38.6, 21.3, 21.2.

HRMS (ESI+): exact mass calculated for $[M-Cl]^+$ ($C_{34}H_{30}N_2O_3PPd$) requires m/z 651.1040, found m/z 651.1042.

^{31}P-NMR (162 MHz, THF-d_8): 130.3 (major isomer), 130.2 (minor isomer).

Relevant chemical shifts and coupling constants are listed in the table:

Major isomer	^{1}H (ppm)	^{13}C (ppm)
1	4.42 (s, 1H)	62.1 (d, J_{P-C} = 2.6 Hz)
2	5.72 (s, 1H)	128.1 (d, J_{P-C} = 2.2 Hz)
3	5.21 (s, 1H)	149.5
4	4.05 (d, J_{P-H} = 5.6 Hz, 1H)	47.1 (d, J_{P-C} = 15.1 Hz)
5	-	182.1

Minor isomer	^{1}H (ppm)	^{13}C (ppm)
1	4.30 (s, 1H)	62.4 (d, J_{P-C} = 2.7 Hz)
2	6.17 (s, 1H)	128.0 (d, J_{P-C} = 2.1 Hz)
3	6.84 (s, 1H)	150.4
4	3.76 (d, J_{P-H} = 8.6 Hz, 1H)	48.2 (d, J_{P-C} = 12.1 Hz)
5	-	182.0

8. Synthesis of intermediate 12a/b

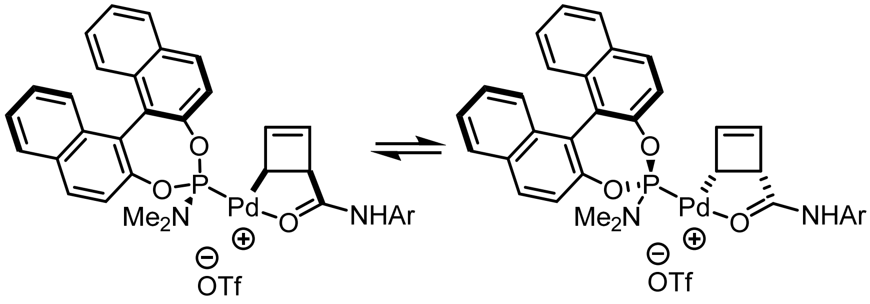

12a **12b**

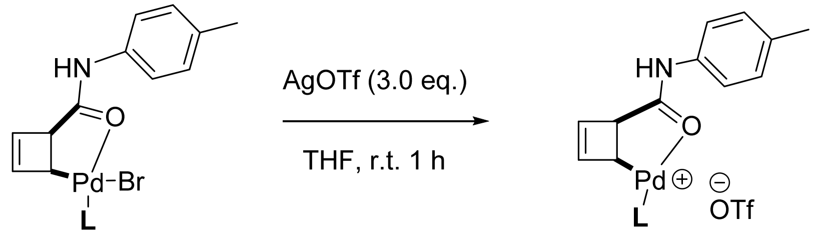

7a/b
2 isomers (1:1.2)

12a/b
2 isomers (1:1.4)

Procedure A: In a schlenk flask (dry and under Argon atmosphere), the bromo/Monophos complex **7a/b** (25 mg, 0.034 mmol, 1 equiv.) and AgOTf (26 mg, 0.102 mmol, 3 equiv.) were added. After three vacuum-Argon cycles, 1.0 mL THF-d_8 was added. The solution was stirred at room temperature for 1h. The crude mixture was filtered through a 25 mm syringe filter (w/ 0.45 µm, PTFE membrane) and characterized by NMR.

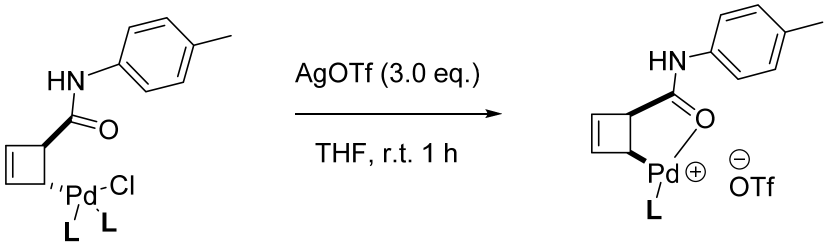

5

12a/b
2 isomers (1:1.4)

Procedure B: In a schlenk flask (dry and under Argon atmosphere), the chloro/Monophos complex **5** (10 mg, 0.015 mmol, 1 equiv.) and AgOTf (17 mg, 0.066 mmol, 4.4 equiv.) were added. After three vacuum-Argon cycles, 1.0 mL THF-d_8 was added. The solution was stirred at room temperature 20 min. The crude mixture was filtered through a 25 mm syringe filter (w/ 0.45 µm, PTFE membrane) and characterized by NMR.

IR (neat) ν_{max}: 3443, 2929, 1688, 1615, 1582, 1558, 1509, 1463, 1368, 1220, 1169, 1021, 948, 831, 761, 729.

^{1}H-NMR (600 MHz, THF-d_8):

The integral ratios are given as observed.

δ 10.74 (bs, 1H), 8.20 (t, J = 9.5 Hz, 1H), 8.12 (d, J = 8.9 Hz, 1H), 8.03 (m, 2H), 7.85 (d, J = 9.1 Hz, 0.7H), 7.82 (d, J = 9.1 Hz, 0.5H), 7.63 (d, J = 8.8 Hz, 0.5H), 7.60 (d, J = 8.6 Hz, 0.7H), 7.53 (d, J = 8.4 Hz, 2H), 7.49 (m, 2H), 7.37 (d, J = 8.5 Hz, 1H), 7.30 (m, 3.5H), 7.12 (m, 2.5H), 6.77 (d, $J_{P\text{-}H}$ = 2.2 Hz, 0.5H), 6.18 (s, 0.5H), 5.86 (s, 0.7H), 5.29 (d, $J_{P\text{-}H}$ = 2.2 Hz, 0.7H), 4.49 (dd, J = 2.8 Hz, $J_{P\text{-}H}$ = 0.9 Hz, 0.7H), 4.35 (dd, J = 2.8 Hz, $J_{P\text{-}H}$ = 0.9 Hz, 0.5H), 4.22 (dm, J = 2.7 Hz, 0.7H), 3.73 (m, 0.5H), 2.82 (s, 3H), 2.81 (s, 3H), 2.26 (s, 3H).

^{13}C-NMR (150 MHz, THF-d_8): δ 181.1, 181.0, 149.4, 149.3, 148.7 (d, $J_{P\text{-}C}$ = 0.8 Hz), 148.57, 148.45, 148.0 (d, $J_{P\text{-}C}$ = 2.2 Hz), 136.0, 135.6, 133.4, 133.3, 133.2, 133.0, 132.7, 132.3, 130.39 (d, $J_{P\text{-}C}$ = 7.4 Hz), 130.37 (d, $J_{P\text{-}C}$ = 7.6 Hz), 130.0, 129.6, 129.6, 129.5, 127.7, 127.6, 127.5, 127.5, 126.6, 126.6, 126.5, 123.7, 123.1, 123.0, 122.9, 122.4, 122.3, 121.9, 121.3, 121.5, 63.69 (d, $J_{P\text{-}C}$ = 1.8 Hz), 63.57 (d, $J_{P\text{-}C}$ = 1.8 Hz), 45.78 (d, $J_{P\text{-}C}$ = 6.2 Hz), 45.42 (d, $J_{P\text{-}C}$ = 5.9 Hz), 37.7, 37.6, 21.0.

HRMS (ESI+): exact mass calculated for $[M\text{-}OTf]^+$ ($C_{34}H_{30}N_2O_3PPd$) requires m/z 651.1040, found m/z 651.1042.

^{31}P-NMR (162 MHz, THF-d_8): 132.6 (major isomer), 132.2 (minor isomer).

^{19}F-NMR (376 MHz, THF-d_8): -78.4.

Relevant chemical shifts and coupling constants are listed in the table:

All measurements were recorded at 298 K without sample spinning on a Bruker Avance III 600 MHz spectrometer equipped with a triple resonance inverse Cryogenically cooled probe (1H/13C/15N, *z*-gradient), unless noted otherwise. A BCU-05 was used for temperature control. Acquisition and processing of NMR data was performed in TopSpin 2.1.

Standard pulse programs from the Bruker library were used for assignment of resonances: 1D ^{1}H and ^{13}C, ^{1}H-^{1}H COSY, ^{1}H-^{13}C HSQC, ^{1}H-^{13}C HMBC and ^{1}H-^{1}H NOESY.

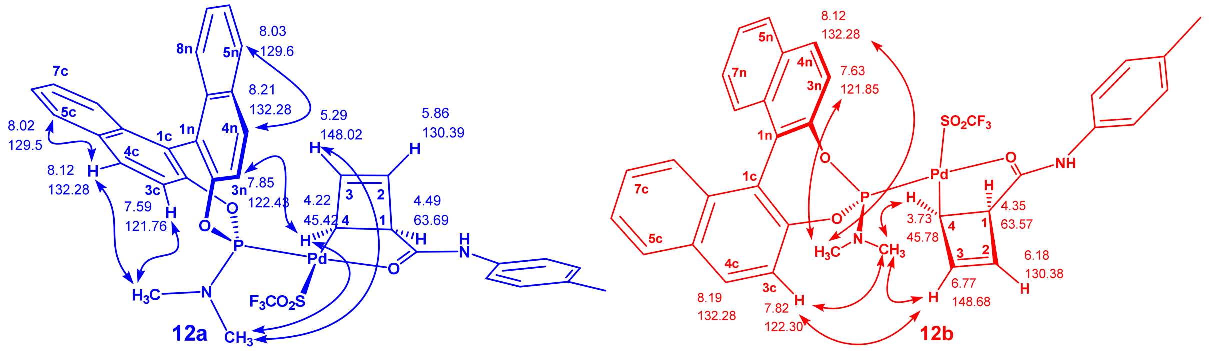

Figure SI-2: Atom numbering used for assignment and NOE analysis. Descriptors "*a*" or "*b*" refer to the corresponding isomers **12a** or **12b**. Naphthyl ring coplanar to cyclobutene is marked with "*c*"-suffix, normal - with "*n*".

Table SI-2: Chemical shifts and assignments of ^{1}H and ^{13}C spectra recorded in THF-*d8* at 298K, including observed COSY, NOESY and HMBC correlations (M=unresolved or only partially resolved multiplet comprising several signals, blue color code: signals belonging to **12a**, red: **12b**; black: non distinguishable/only one signal set observed).

Assign.	^{1}H ($J_{(C,H)}$)	^{13}C ($J_{(C,P)}$)	HH-COSY	HMBC ($J_{(C,P)}$)	NOESY
CH_3	2.27	21.0	7.12 (^{4}J)<br>7.53 (^{5}J)	130.0(*m*Tol)<br>136.0 (C_{quat})	*m*Tol
NMe	2.82<br>2.80	37.67<br>37.60	-<br>-	-<br>-	3-a, 4-a<br>7.59(3c), 8.12(4c)<br>3-b, 4-b<br>7.82(3c),<br>7.63(3n),8.12(4n)
*c*B-4_b	3.73	45.78 (6 Hz)	6.77 (3-b)<br>6.18 (2-b)<br>4.35 (1-b)	130.4 (2-b)<br>148.7 (3-b)	NMe, 1-b, 3-b
*c*B-4_a	4.22	45.42 (6 Hz)	5.29 (3-a)<br>5.86 (2-a)<br>4.49 (1-a)	130.4 (2-a)<br>148.0 (3-a)	NMe, 1-a, 3-a<br>3-BNn_a
*c*B-1_b	4.35	63.57 (2 Hz)	6.77 (3-b)<br>6.18 (2-b)<br>3.73 (4-b)	130.4 (2-b)<br>148.7 (3-b)<br>45.8 (4-b)<br>181.0	2-b, 4-b
*c*B-1_a	4.49	63.69 (2Hz)	5.29 (3-a)<br>5.86 (2-a)<br>4.22 (4-a)	130.4 (2-a)<br>148.0 (3-a)<br>45.4 (4-a)	2-a, 4-a

				181.1	
*c*B-3_a	5.29	148.02 (2 Hz)	5.86 (2-a)<br>4.49 (1-a)<br>4.22 (4-a)	63.7 (1-a)<br>45.4 (4-a)	2-a, 4-a, NMe
*c*B-2_a	5.86	130.39 (7Hz)	5.29 (3-a)<br>4.49 (1-a)<br>4.22 (4-a)	45.4 (4-a)<br>63.7 (1-a)<br>148.0 (3-a)	1-a, 3-a
*c*B-2_b	6.18	130.37 (7 Hz)	6.77 (3-b)<br>4.35 (1-b)<br>3.73 (4-b)	45.8(4-b)<br>63.6 (1-b)<br>148.7 (3-b)	$CONH_b$, 1-b,<br>3-b
*c*B-3_b	6.77	148.68 (1 Hz)	5.99 (2-b)<br>4.35 (1-b)<br>3.73 (4-b)	45.8 (4-b)<br>63.6 (1-b)<br>130.4 (2-b)	3-$BNc_{b,}$ NMe,<br>2-b, 4-b
*m*Tol	7.12	130.0<br>130.1	2.26 (CH_3)<br>7.53 (*o*Tol)	21.0 (CH_3)<br>130.0, 130.1 (mTol)<br>121.54, 121.55 (*o*Tol)<br>135.58, 135.56 (*C*-NHCO-)	CH_3
M1, 6H<br>7-BNn_{ab}<br>7-BNc_{ab}<br>8-BNn_a<br><br><br>8-BNc_b	7.27-7.34<br><br><br>7.32<br><br><br>7.32	127.48<br>127.55<br>127.58<br>127.66<br>127.68<br>127.71	M2<br>7.32(8-BNn)<br>7.37 (8-BNc)<br>M1<br><br><br>M1	 <br><br>123.74 (1-BNn_a)<br>126.64 (6-BNn_a)<br>132.92 (4a-BNn_a)<br>123.74 (1-BNc_b)<br>126.56 (6-BNc_b)<br>132.99 (4a-BNc_b)	M3<br><br><br>-<br><br><br>-
8-BNc_a	7.37	127.48	M1	123.06 (1-BNc_a)<br>126.49 (6-BNc_a)<br>132.73 (4a-BNc_a)	-
8-BNn_b	7.37	127.48	M1	123.04 (1-BNn_b)<br>126.49 (6-BNn_b)<br>132.75 (4a-BNn_b)	-

M2, 4H<br>6-BNn$_{ab}$<br>6-BNc$_{ab}$	7.46-7.51	126.49<br>126.49<br>126.56<br>126.64	M1<br>8.02<br>8.03	127.48, 127.55 (8-BN)<br>133.00, 132.92<br>132.75, 132,73	M1<br>8.02<br>8.03
*o*Tol	7.53	121.54<br>121.55	7.12 (*m*Tol)	135.99 (*C*-CH_3)<br>136.00 (*C*-CH_3)	NH,<br>*c*B-1$_a$, CH_3
3-BNc$_a$	7.59	121.76	8.12	132.73<br>149.4 (2-BNc$_a$)<br>123.1 (1-BNc$_a$)	NMe<br>4-BNc$_a$
3-BNn$_b$	7.63	121.85	8.12	132.75<br>149.4 (2-BNn$_b$)<br>123.0 (1-BNn$_b$)	NMe
3-BNc$_b$	7.82	122.30	8.19	132.99<br>148.46 (2-BNc$_b$)<br>123.74 (1-BNc$_b$)	NMe<br>*c*B-3$_b$
3-BNn$_a$	7.85	122.43	8.21	132.92<br>148.58 (2-BNn$_a$)<br>123.74 (1-BNn$_a$)	*c*B-4$_a$
5-BNc$_a$<br>5-BNc$_b$<br>5-BNn$_a$<br>5-BNn$_b$	8.02<br>8.03<br>8.02<br>8.03	129.5<br>129.6<br>129.5<br>129.6	7.46-7.51<br>M2	127.71 – 127.48 (7-BN)<br>132.28 (4-BN)<br>133.22, 133.30, 133.38	M2<br>4-BNc$_a$<br>4-BNc$_b$
4-BNc$_a$	8.12	132.28	7.59	149.43*<br>133.22	NMe<br>8.02(5BNc$_a$)
4-BNn$_b$	8.12	132.28	7.63	149.45*<br>133.22	NMe<br>8.02(5BNn$_b$)
4-BNc$_b$	8.19	132.28	7.82	148.46<br>133.38	8.03(5BNc$_b$)
4-BNn$_a$	8.21	132.28	7.85	148.58<br>133.30	5.29 (*c*B-3$_a$)<br>8.03(5BNc$_a$)
CONH$_a$<br>CONH$_b$	10.71<br>10.71			181.11, 121.54 (*o*Tol)<br>181.03, 121.55 (*o*Tol)	7.53 (*o*Tol)<br>4.49 (*c*B-1$_a$), 5.86 (*c*B-2$_a$)<br>4.35 (*c*B-1$_b$), 6.18 (*c*B-

					2_b)
1-BNn$_b$		123.04 (2 Hz)			
1-BNc$_a$		123.06 (2 Hz)			
1-BNn$_a$<br>1-BNc$_b$		123.74 (2 Hz)<br>123.74 (2 Hz)			
4a-BNc$_a$		132.73			
4a-BNn$_b$		132.75			
4a-BNn$_a$		132.92			
4a-BNc$_b$		132.99			
8a-BNc$_a$<br>8a-BNn$_b$		133.22<br>133.22			
8a-BNn$_a$		133.30			
8a-BNc$_b$		133.38			
C-NHCO-$_b$		135.56			
C-NHCO-$_a$		135.58			
-*C*-CH_{3a}		135.99			
-*C*-CH_{3b}		136.00			
2-BNn$_b$		148.46 (5 Hz)			
2-BNc$_a$		148.58 (5 Hz)			
2-BNn$_a$		149.39 (13 Hz)			
2-BNc$_b$		149.40 (13 Hz)			
CO-NH-$_b$		181.03			
CO-NH-$_a$		181.11			
CF_3SO_2		121.9 (J_{CF}=320 Hz)			

* interchangeable

9. Reaction of 7a/b and 12a/b with diethyl 2-methylmalonate sodium salt

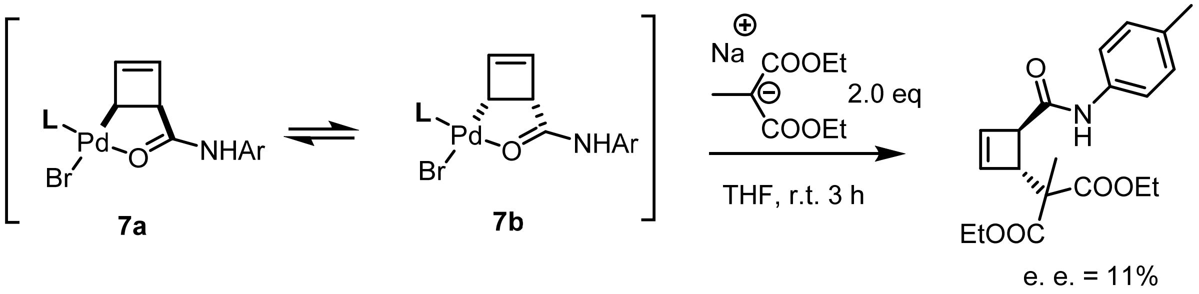

In a schlenk flask (dry and under Argon atmosphere), the bromo/Monophos complex **7a/b** (36.6 mg, 0.05 mmol, 1 equiv.) and THF 1 mL were added. Then a freshly prepared 1 M diethyl 2-methylmalonate sodium salt in THF was added. The mixture was allowed to stir at room temperature for 3 h before quenched with water. After extracted by DCM (3 X 5 mL) and dried on $MgSO_4$, the crude product was purified by column on silica gel (hexane:ethyl acetate 5:1).
If the reaction was conducted at 0 ˚C, after 3 h, only starting complexes were detected.

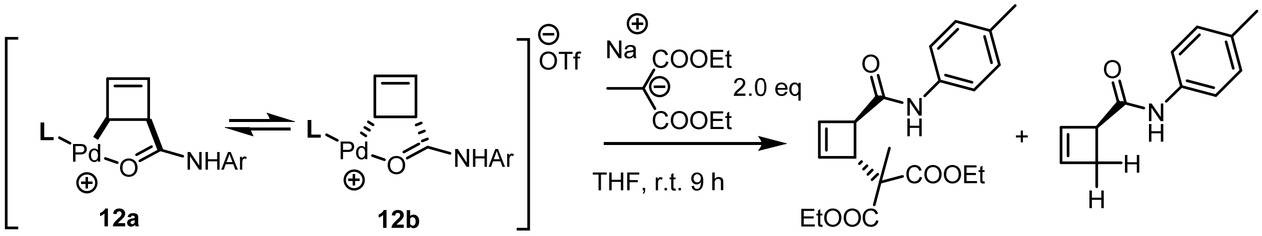

2 : **1**

In a schlenk flask (dry and under Argon atmosphere), the triflate/Monophos complex **12a/b** (36.6 mg, 0.05 mmol, 1 equiv.) and THF 1 mL were added. Then a freshly prepared 1 M diethyl 2-methylmalonate sodium salt in THF was added. The mixture was allowed to stir at room temperature overnight before quenched with water. After extracted by DCM (3 X 5 mL) and dried on $MgSO_4$, the crude product was purified by column on silica gel (hexane:ethyl acetate 5:1).

Diethyl 2-methyl-2-(*trans*-4-(*p*-tolylcarbamoyl)cyclobut-2-en-1-yl)malonate

^{1}H-NMR (500 MHz, $CDCl_3$): δ 9.33 (bs, 1H), 7.50 (d, J = 8.5 Hz, 2H), 7.11 (d, J = 8.5 Hz, 2H), 6.36 (d, J = 3.1 Hz, 1H), 6.08 (d, J = 3.0 Hz, 1H), 4.25 (m, 4H), 3.70 (s, 1H), 3.52 (s, 1H), 2.31 (s, 3H), 1.28 (m, 6H).

^{13}C-NMR (125 MHz, $CDCl_3$): δ 172.2, 172.1, 170.0, 137.1, 136.3, 133.2, 129.5, 119.4, 62.1 (2C), 55.3, 51.5, 49.9, 21.0, 17.6, 14.2 (2C).

HRMS (ESI+): exact mass calculated for $[M+Na]^+$ ($C_{20}H_{25}NNaO_5$) requires m/z 382.1630, found m/z 382.1625.

N-(*p*-tolyl)cyclobut-2-ene-1-carboxamide

^{1}H-NMR (500 MHz, CDCl3): δ 7.40 (d, J = 8.4 Hz, 2H), 7.31 (bs, 1H), 7.12 (d, J = 8.2 Hz, 2H), 6.42 (d, J = 2.5 Hz, 1H), 6.17 (d, J = 2.5 Hz, 1H), 3.70 (d, J = 4.1 Hz, 1H), 2.97 (dd, J = 14.0, 4.7 Hz, 1H), 2.72 (d, J = 13.9 Hz, 1H), 2.31 (s, 3H).

^{13}C-NMR (125 MHz, CDCl3): δ 171.1, 141.9, 135.4, 135.1, 134.0, 49.3, 36.1, 21.0.

HRMS: exact mass calculated for $C_{12}H_{13}NO$ requires m/z 187.0997, found m/z 187.0968.

10. Enrichment of the diastereomeric mixture 7a/7b with isomer 7a

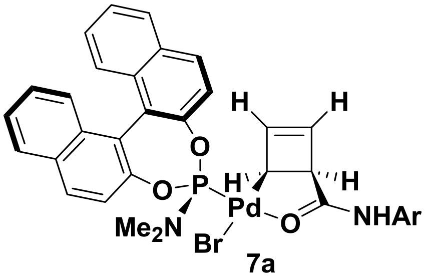

7a

The procedure is based on a limited solubility of **7a** in THF. Thus, the crude product obtained from 14 mg of *para*-toluamide of 4-bromocyclobut-2-enecarboxylic acid as described above (note, it is actually NMR-tube containing the solid product, *vide infra*), was triturated at 0–5 °C with THF-d_8 (0.5 mL) by occasional shaking. When the solids settled down, the supernatant solution was carefully cannulated into another NMR-tube and **7a**:**7b**-ratio was checked (it may vary but never exceeds 45:55 – which is the equilibrium point). To the residue in the 1st tube another portion of THF-d_8 (0.5 mL) was added at 0 °C and, after occasional shaking, it was subjected to NMR analysis at 0–5 °C. At this point, it typically shows 10-fold enrichment with **7a**-isomer. Further enrichment can be achieved after repeated decantation-dissolving sequence and can ultimately furnish *d.r.* as high as 20:1 at its best (see Figure SI-**3**).

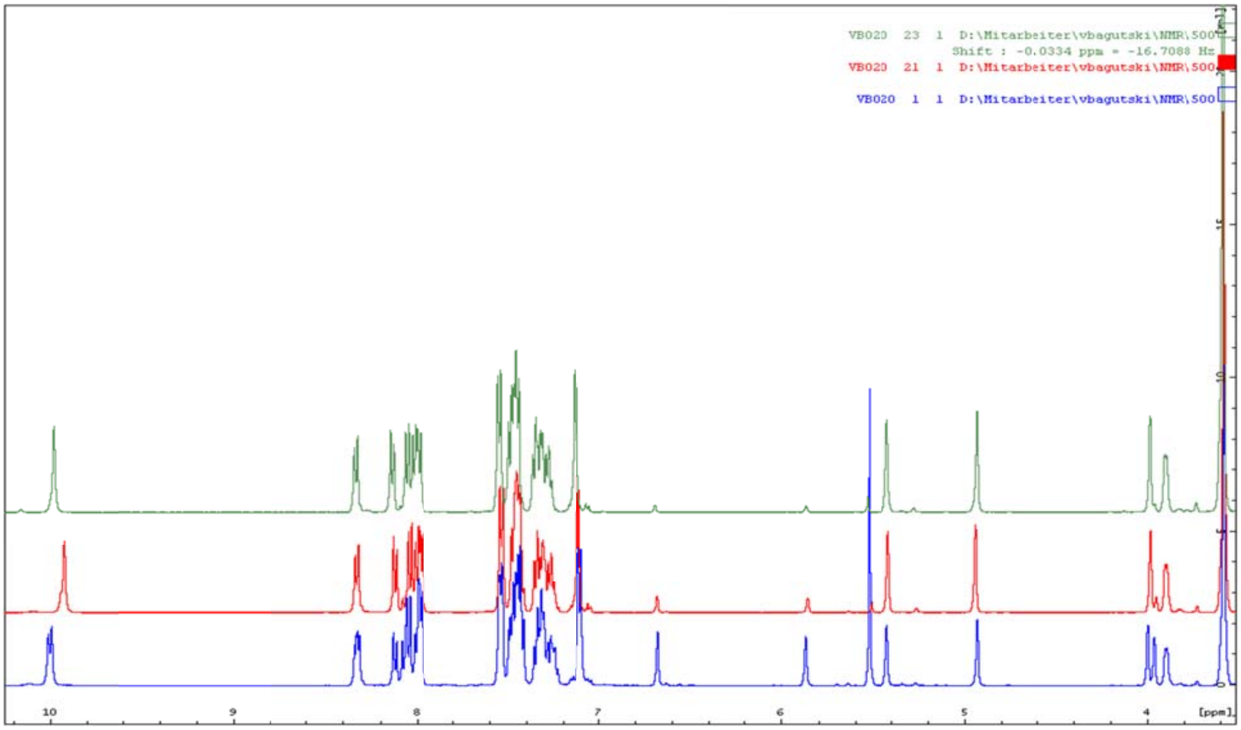

Figure SI-3: Enrichment of the isomeric mixture with isomer **7a**. Blue – initial mixture, **7a/7b** = 6:5; red – 1st depletion, **7a/7b** = 6:1; green – 2nd depletion, **7a/7b** = 20:1.

11. X-Ray Crystallography of 7a

Sample Preparation. Single crystal of **7a** suitable for X-Ray diffraction data collection was grown after dissolving the crude **7** (contained some miniscule amounts of ethanol remaining after concentration of the chromatographic fractions at ambient temperature on rotary evaporator) in CH_2Cl_2 and allowing it to crystallize in a fridge (+4 °C) over 1 week.

X-Ray data were collected using a IPDS II (Fa. Stoe&Cie GmbH, Darmstadt) diffractometer.

The crystal structure was determined in the monoclinic space group $P\ 2_1$ using direct methods (SHELXL-97) and refined on F^2 (SHELXS-97). The positions of the hydrogen atoms were calculated using HFIX. All non-hydrogen atoms with the exception of atom C33 were anisotropically refined. The crystals contain solvent water (two molecules in the asymmetric unit). Full details regarding the crystal structure determination are deposited in the Cambridge Crystallographic Database (CCDC 991087).

Table SI-3: Crystallographic data and structure refinement for **7a**.

Empirical formula	$C_{34}H_{30}BrN_2O_5PPd$
Formula weight	729.90 (765.93)

Spacegroup	*P* 2_1 (No. 4)
Crystal system	monoclinic
Z	2
Temperature	193.2 K
Unit cell dimensions	*a* = 956.6(2) pm, α = 90°
	b = 1064.9(2) pm, β = 95.18(3)°
	c = 1711.5(3) pm, γ = 90°
Volume	1736.4(6)×10^6 pm^3
Wavelength	71.073 pm
Diffractometer	IPDS II. Fa. Stoe&Cie GmbH. Darmstadt
Reflections collected	15415
Independent reflections	4118
Refined parameters	396
$\delta_{min}/\delta_{max}$ (e/10^6 pm^3)	–0.50/1.41
R_{int}	0.1429
R_1 (F_0> 4 σ)	0.0590
R_1 (all Reflections)	0.0909
wR_2	0.1127
Goodness–of–fit on F^2	1.151

12. Dynamics studies by selective 1D PFGSE NOE Spectra

To study the temperature dependence of the allylic migration of Pd interconverting the two species **7a** into **7b**, multiple series of selective 1D PFGSE NOE spectra[2-4] with varying mixing times (τ_m = 300 – 650 ms) and relaxation delays > 5*T_1 were acquired. An adiabatically swept-frequency pulse/gradient pair was used to suppress unwanted contributions to the NOE/exchange signal by zero-quantum transitions.[5] The offset of the selective inversion (80ms Gaussian shaped pulse) was set to the chemical shift of H2 in **7a** and the intensity of the exchange peak at the shift of H4 in **7b** was observed as a function of the mixing time. Similarly the exchange of H2 in **7b** to H4 in **7a** was used to study the back reaction. All exchange signals were normalized with respect to the inverted signal according to the PANIC method.[6] At temperatures up to 320K this exchange could only be observed in DMSO, whereas only NOE signals were observed in THF.

Table SI-4: Integrals table of the H2→H4 exchange measured at 320K.

Mixing time (τ_m) / s	Normalized intensity, I_{norm} / a.u.	
	H2→H4	H2→H4
0.30	0.0071	0.0093
0.35	0.0085	0.0115
0.40	0.0102	0.0137
0.45	0.0114	0.0153
0.50	0.0128	0.0177
0.55	0.0140	0.0202
0.60	0.0151	0.0226
0.65	0.0166	0.0246

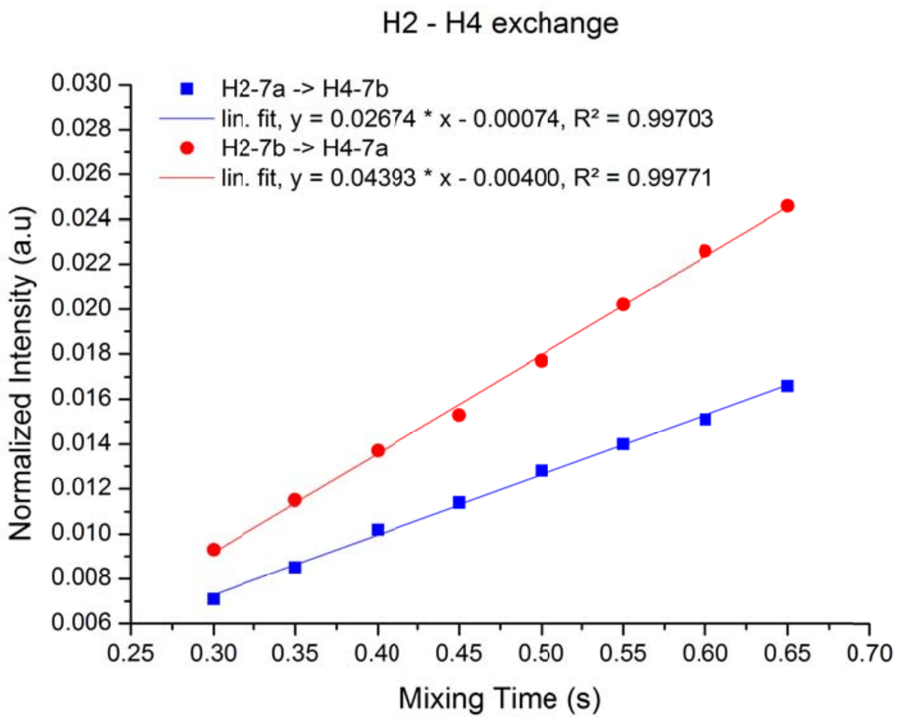

Figure SI-4: Arrhenius plot of the mixing series H2→H4 (blue, H2 of **7a** was irradiated at 5.60 ppm and the intensity of H4-**7b** at 3.58 ppm was measured) and H2→H4 (red, H2 of **7b** was irradiated at 5.99 ppm and intensity of H4-**7a** at 3.95 ppm was measured) at T = 320 K.

The slightly higher value of the equilibrium constant derived from the ratio $k_{(H2\rightarrow H4)}/k_{(H2\rightarrow H4)}$ (1.62) compared to the product ratio observed in the equilibrated **7a**/**7b**-mixture (1.26)[3] might be attributed to a higher decomposition rate of **7b**, which is evidenced by slowly growing exchange peak at 5.74 ppm while irradiating H2 (see Figure SI-5, top). The relative intensity of this peak compared at equal conditions (the same measuring session, mixing time 500 ms for both) to the analogous decomposition product's peak of **7a** (5.39 ppm, Figure SI-5, bottom) yielded 1.28 which after multiplying by 1.26 would match the observed *K*-value almost perfectly (1.61).

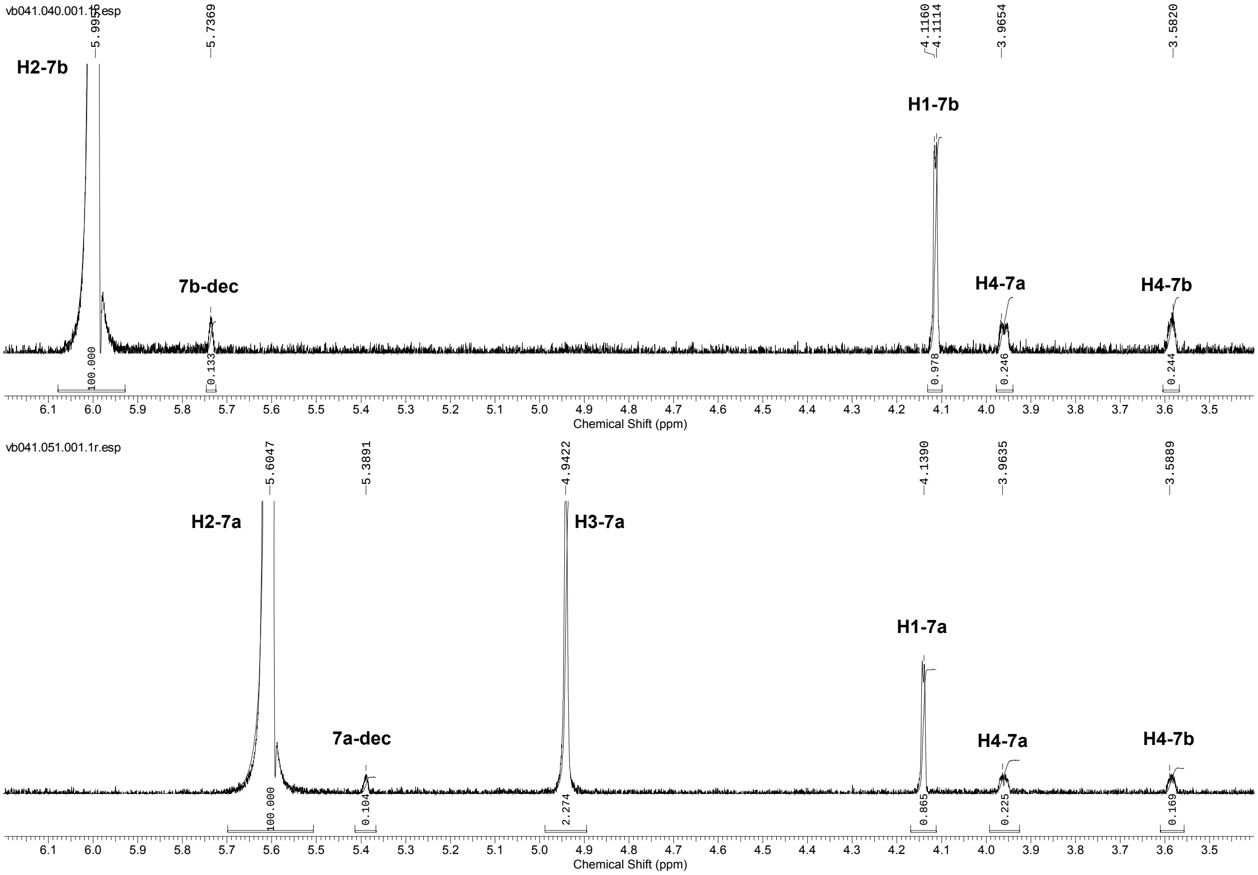

Figure SI-5: Selective 1D PFGSE NOE spectra (τ_m = 500ms) of **7** in DMSO-d_6 at 300K. **Top:** The signal of H2 in 7b at 6.0 ppm was selectively inverted and the corresponding exchange signal of H4 in **7a** at 3.9 ppm observed. **Bottom:** The signal of H2 in **7a** at 5.6 ppm was selectively inverted and the corresponding exchange signal of H4 in **7b** at 3.6 ppm observed. Apart from the intramolecular NOEs and **7a**↔**7b** exchange signals, the spectra also show small signals of a decomposition product (labeled **7a-dec** and **7b-dec**, respectively).

[3] **7a**/**7b**-ratio was calculated using the relative intensities of H2-peaks (at 5.60 and 5.99 ppm respectively) for both isomers obtained from 1-scan ^{1}H-NMR spectra of both isomers.

13. Calculation of Structure Models for 7a/b by DFT

Based on the crystal structure of **7a**, structure models to be used in the RDC fits were calculated by DFT using the ORCA[7] software package. Geometry optimization and subsequent frequency calculation was performed using the B3LYP functional with the def2-TZVP basis set[8] for all atoms except Pd and Br, for which the appropriate 28-electron effective core potentials from the Stuttgart/Cologne group[9, 10] were used, as implemented in ORCA v2.9.1. Based on the optimized geometry for **7a**, the cyclobutene fragment was rotated along the C1–C=O bond (numbering see Figure SI-6) and the full structure reoptimized to give the geometry of **7b** as well as the transitional η^3-coordinated species **7-η^3**.

Cartesian Coordinates of 7a

```
Pd0       -0.7694804623   -0.1079632836    1.4265589054
Br0       -1.5808014623    2.3734857164    1.5382509054
P14       -1.5849794623   -0.3004842836   -0.6947300946
O5         0.0660475377   -0.0817762836    3.4461169054
O16a      -1.3220004623    0.8722147164   -1.8142760946
O16b      -0.8325214623   -1.5591092836   -1.4880160946
N13       -3.2292584623   -0.4376562836   -0.8241130946
N6         1.2385025377   -1.2820552836    4.9766369054
C24a       1.9205075377    0.5191727164   -3.5382490946
C16a      -0.0168484623    1.1043827164   -2.2346900946
C15a       0.5536555377    0.2683877164   -3.1737500946
C24b      -0.4298464623   -1.0620592836   -5.1376420946
C8a        2.3040565377   -0.7285112836    7.0544339054
C17a       0.6334915377    2.2271957164   -1.6927650946
C19a       2.5847155377    1.6694207164   -3.0070950946
C7         1.6075525377   -0.2952002836    5.9262269054
C23b       0.0478205377   -0.1500272836   -6.1145140946
C8b        1.3329455377    1.0658027164    5.7740879054
C21b      -0.7984304623   -1.5370092836   -7.9047760946
C21a       4.6041795377    1.0978427164   -4.2265920946
C10        2.5018405377    1.5492017164    7.8582289054
C20b      -1.3054834623   -2.4216662836   -6.9909090946
C23a       2.6705305377   -0.3457382836   -4.3758790946
C15b      -0.2504414623   -0.8558492836   -3.7256490946
C17b      -1.6302214623   -2.8395772836   -3.3198390946
C18b      -1.7340784623   -3.0877602836   -4.6583000946
C22b      -0.1268894623   -0.3818492836   -7.4551280946
C1         0.5412825377   -2.3656732836    2.9126549054
C9a        2.7447395377    0.1843877164    8.0016079054
C18a       1.9065605377    2.5132637164   -2.0952320946
C20a       3.9230165377    1.9368067164   -3.3848690946
C16b      -0.9052994623   -1.7206522836   -2.8650990946
C9b        1.7837005377    1.9611767164    6.7339219054
C5         0.5841765377   -1.1507542836    3.8022019054
C22a       3.9705535377   -0.0640592836   -4.7119060946
C19b      -1.1546194623   -2.2047422836   -5.5997130946
C2         1.7989325377   -2.6183242836    2.0844999054
C3         1.1791395377   -2.3983062836    0.9150419054
C4        -0.1478234623   -2.0983182836    1.5165759054
C12b      -3.9725924623   -0.1933072836   -2.0596200946
C12a      -4.0584574623   -0.5136002836    0.3755079054
C11        3.0002495377    2.5458297164    8.8721809054
H6         1.6095685377   -2.2012362836    5.1607069054
H8a        2.5177885377   -1.7840262836    7.1890029054
H17a       0.1044315377    2.8240157164   -0.9613200946
H23b       0.5552765377    0.7476127164   -5.7937810946
H8b        0.7763235377    1.4176737164    4.9205529054
H21b      -0.9256684623   -1.7160112836   -8.9653300946
```

H21a	5.6244405377	1.3204867164	-4.5137940946
H20b	-1.8476944623	-3.2999822836	-7.3210820946
H23a	2.2082725377	-1.2444042836	-4.7580320946
H17b	-2.0879464623	-3.4866352836	-2.5835180946
H18b	-2.2812134623	-3.9522882836	-5.0143040946
H22b	0.2513495377	0.3339067164	-8.1747180946
H1	0.1643725377	-3.2345932836	3.4631379054
H9a	3.2939315377	-0.1762412836	8.8633739054
H18a	2.4215475377	3.3785997164	-1.6959900946
H20a	4.4030785377	2.8204907164	-2.9806840946
H9b	1.5660655377	3.0140157164	6.5925579054
H22a	4.5165175377	-0.7452012836	-5.3528750946
H2	2.8089835377	-2.8822092836	2.3715869054
H3	1.5065345377	-2.4368472836	-0.1149400946
H4	-0.9760084623	-2.7583752836	1.2604449054
H12ba	-3.3029954623	-0.1432072836	-2.9123210946
H12bb	-4.5174394623	0.7533417164	-1.9946690946
H12bc	-4.6913824623	-1.0007992836	-2.2229920946
H12aa	-3.4337784623	-0.6322372836	1.2589229054
H12ab	-4.7428394623	-1.3642792836	0.3059189054
H12ac	-4.6389324623	0.4049177164	0.4960029054
H11a	2.1747155377	3.0854897164	9.3440419054
H11b	3.6465015377	3.2913547164	8.4016769054
H11c	3.5724165377	2.0542587164	9.6598379054

Cartesian Coordinates of 7b

Pd0	-0.7451812311	-0.1125985228	1.3740148215
Br0	-1.7133772311	2.3058134772	1.6098318215
P14	-1.5697592311	-0.2553405228	-0.7446561785
O5	0.0844807689	-0.1697665228	3.3987988215
O16a	-1.2705212311	0.9305194772	-1.8411781785
O16b	-0.8478962311	-1.5207125228	-1.5556311785
N13	-3.2152682311	-0.3555655228	-0.8798411785
N6	1.5440747689	-1.2753655228	4.7424178215
C24a	2.0386587689	0.5513474772	-3.4212091785
C16a	0.0533407689	1.1524264772	-2.2028091785
C15a	0.6518867689	0.3210974772	-3.1278141785
C24b	-0.2644322311	-0.9390755228	-5.1666101785
C8a	2.7399117689	-0.6745485228	6.7342778215
C17a	0.6961097689	2.2553574772	-1.6130691785
C19a	2.6965207689	1.6829544772	-2.8436441785
C7	1.8190577689	-0.3142845228	5.7489858215
C23b	0.2825977689	-0.0155955228	-6.0947611785
C8b	1.2405837689	0.9559844772	5.7874978215
C21b	-0.5026962311	-1.3388135228	-7.9596331785
C21a	4.7602377689	1.0905964772	-3.9755021785
C10	2.5702027689	1.5155644772	7.7540818215
C20b	-1.0781962311	-2.2312705228	-7.0950881785
C23a	2.8119727689	-0.3173195228	-4.2330381785
C15b	-0.1491232311	-0.7722395228	-3.7428421785
C17b	-1.5938142311	-2.7301735228	-3.4556001785
C18b	-1.6395982311	-2.9421125228	-4.8036781785
C22b	0.1718647689	-0.2112395228	-7.4479321785
C1	0.7920347689	-2.3352315228	2.6782358215
C9a	3.1097857689	0.2310854772	7.7170878215
C18a	1.9902087689	2.5269694772	-1.9528881785
C20a	4.0548067689	1.9316414772	-3.1558761785
C16b	-0.8650652311	-1.6413225228	-2.9370111785
C9b	1.6250907689	1.8458944772	6.7816268215
C5	0.7801597689	-1.1699425228	3.6332018215
C22a	4.1308107689	-0.0550495228	-4.5037331785
C19b	-0.9926192311	-2.0515885228	-5.6928581785
C4	0.0832867689	-2.0239735228	1.3021498215
C3	-0.8622002311	-3.1135215228	1.6546458215
C2	-0.3132762311	-3.3748075228	2.8511268215
C12b	-4.0505842311	-0.4291015228	0.3170448215
C12a	-3.9452652311	-0.0678175228	-2.1139131785
C11	3.0145567689	2.5232564772	8.7825998215
H6	2.0760357689	-2.1281895228	4.8175398215
H8a	3.1806607689	-1.6660375228	6.7276908215
H17a	0.1429667689	2.8509494772	-0.8985581785

H23b	0.7942337689	0.8612414772	-5.7266511785
H8b	0.5144647689	1.2482034772	5.0463838215
H21b	-0.5774282311	-1.4904105228	-9.0293371785
H21a	5.7960927689	1.2992484772	-4.2130441785
H20b	-1.6242792311	-3.0874385228	-7.4736291785
H23a	2.3518377689	-1.2034015228	-4.6458991785
H17b	-2.1005582311	-3.3849965228	-2.7592991785
H18b	-2.1892732311	-3.7836345228	-5.2077991785
H22b	0.6031497689	0.5118374772	-8.1292921785
H1	1.7972007689	-2.7593015228	2.5855538215
H9a	3.8332657689	-0.0700125228	8.4655238215
H18a	2.4997647689	3.3785064772	-1.5186271785
H20a	4.5305527689	2.8022384772	-2.7195151785
H9b	1.1733847689	2.8313524772	6.7898318215
H22a	4.6950607689	-0.7385705228	-5.1260501785
H4	0.6859027689	-2.1216465228	0.4018228215
H3	-1.6966612311	-3.5466575228	1.1181948215
H2	-0.5393802311	-4.0597465228	3.6576988215
H12ba	-3.4350462311	-0.5936715228	1.1988588215
H12bb	-4.7646102311	-1.2525265228	0.2221258215
H12bc	-4.5962302311	0.5068704772	0.4609558215
H12aa	-3.2737472311	-0.0474965228	-2.9667101785
H12ab	-4.4435542311	0.9038614772	-2.0455731785
H12ac	-4.7021162311	-0.8389795228	-2.2803491785
H11a	2.1641767689	3.0412954772	9.2318538215
H11b	3.6522637689	3.2855594772	8.3255418215
H11c	3.5840687689	2.0496504772	9.5831758215

Cartesian Coordinates of 7-η^3

Pd0	1.2728751892	-0.0793068761	-0.9363488225
Br0	0.8603771892	-0.5635268761	1.4674251775
P14	-0.9029288108	0.8631901239	-1.3223268225
O5	3.4366641892	1.6831821239	-0.6508158225
O16a	-2.1999918108	1.4470451239	-0.2430458225
O16b	-1.7066598108	-0.6789258761	-1.7368788225
N13	-1.1430208108	2.0287671239	-2.5695858225
N6	5.5863181892	0.7490491239	-0.7389758225
C24a	-3.2424018108	-1.3074618761	2.0743411775
C16a	-2.4638058108	0.7061111239	0.9324381775
C15a	-3.1582898108	-0.5131878761	0.8573921775
C24b	-5.2169888108	-1.2582938761	-0.4826998225
C8a	7.7106671892	1.2494951239	0.3237871775
C17a	-2.0079228108	1.2638641239	2.1599101775
C19a	-2.7592598108	-0.7497828761	3.3268711775
C7	6.3357111892	1.5570261239	0.1610831775
C23b	-6.0940878108	-1.0489268761	0.6356251775
C8b	5.7695461892	2.6207641239	0.9089511775
C21b	-8.0142118108	-1.9008888761	-0.6330878225
C21a	-3.3404358108	-2.8333268761	4.5022151775
C10	7.9620931892	3.0618201239	1.9662241775
C20b	-7.2004818108	-2.0911928761	-1.7496488225
C23a	-3.7384518108	-2.6549998761	2.0876231775
C15b	-3.7936718108	-0.9412688761	-0.4301468225
C17b	-3.6570558108	-1.4832428761	-2.8514928225
C18b	-4.9848598108	-1.8866968761	-2.8814378225
C22b	-7.4536118108	-1.3598558761	0.5619001775
C1	3.7812731892	-0.3889858761	-1.9044358225
C9a	8.5077291892	1.9920461239	1.2126071775
C18a	-2.1847238108	0.5630181239	3.3432901775
C20a	-2.8348418108	-1.5342068761	4.5239891775
C16b	-3.0718988108	-0.9979508761	-1.6405608225
C9b	6.5851191892	3.3531201239	1.7935051775
C5	4.2420081892	0.7970311239	-1.0648148225
C22a	-3.7838218108	-3.3984068761	3.2689271775
C19b	-5.8048048108	-1.7628568761	-1.7122748225
C4	2.9173581892	-1.4933058761	-1.1886498225
C3	2.0415981892	-1.5778738761	-2.3480718225
C2	2.5155501892	-0.3051838761	-2.8307228225
C12b	-2.4579948108	2.4471481239	-3.0700978225
C12a	0.0347631892	2.6107001239	-3.2275558225
C11	8.8231351892	3.8763521239	2.9100181775
H6	6.1197011892	-0.0161238761	-1.1683548225
H8a	8.1601081892	0.4123851239	-0.2407778225

H17a	-1.5011328108	2.2394931239	2.1314401775
H23b	-5.6862368108	-0.6229848761	1.5641611775
H8b	4.7038181892	2.8571161239	0.7848041775
H21b	-9.0862438108	-2.1554248761	-0.6735348225
H21a	-3.3855298108	-3.4299998761	5.4291221775
H20b	-7.6213928108	-2.4863188761	-2.6907908225
H23a	-4.0686948108	-3.1110348761	1.1414001775
H17b	-3.0104758108	-1.5437378761	-3.7410328225
H18b	-5.4283488108	-2.2837618761	-3.8106018225
H22b	-8.1006498108	-1.1823088761	1.4373181775
H1	4.6452841892	-0.8446548761	-2.4519928225
H9a	9.5720551892	1.7263691239	1.3357271775
H18a	-1.8329058108	0.9835281239	4.3003521775
H20a	-2.4645718108	-1.0928098761	5.4654891775
H9b	6.1292691892	4.1758191239	2.3720291775
H22a	-4.1576968108	-4.4355448761	3.2474741775
H4	3.1688691892	-2.1911528761	-0.3759918225
H3	1.3265601892	-2.3254258761	-2.7158048225
H2	2.3468141892	0.2534241239	-3.7632968225
H12ba	-2.5355318108	3.5605431239	-3.0603548225
H12bb	-3.2585638108	2.0419591239	-2.4175358225
H12bc	-2.6215548108	2.0920021239	-4.1175318225
H12aa	0.9597491892	2.2983171239	-2.6913018225
H12ab	0.1140251892	2.2837471239	-4.2942428225
H12ac	-0.0192758108	3.7254021239	-3.2045398225
H11a	9.7508501892	3.3302381239	3.1925521775
H11b	9.1379811892	4.8438671239	2.4481671775
H11c	8.2759501892	4.1281221239	3.8473461775

14. Validation of Structure Models for 7a/b using Residual Dipolar Couplings

Sample Preparation. A PDMS-stick (14 × 2.5 mm) – the preparation of which will be subject to a further report – was carefully immersed under argon atmosphere into an NMR-tube containing a saturated solution of a **7a**/**7b**-mixture at such a depth, that the middle point of the stick would be positioned exactly in the middle of the NMR-probe used for measurements, and left to swell at ambient temperature to fix it to the walls of the NMR-tube (~10-15) min. After the sample was equilibrated at ambient temperature (~20 °C for 5 days), it was subjected to NMR-study.

NMR Measurements. Isotropic $^{1}J_{CH}$ and anisotropic $^{1}T_{CH}$ couplings (see Table SI-5) were extracted from scaled ω_1-coupled HSQC spectra using a G-BIRD(r) filter to suppress long-range couplings (Bruker pulse program *hsqcbietgpjcsp.2*).[11] Spectra were acquired with spectral widths of 7 ppm and 160 ppm for the direct and indirect dimension, respectively. 1024 points were sampled for the FID in the direct dimension, while 2048 points were sampled linearly for the indirect dimension. Delays were optimized to $^{1}J_{CH}$ = 160 Hz and a scaling factor of 8 was applied for the *J*-evolution in the indirect dimension. Shaped pulses were used for broadband inversion (Crp60,0.5,20.1) and refocusing (Bip720,100,10.1), where appropriate. Total experiment times were 18h10min each.

RDC Fits. Calculation of molecular order tensors and derived quality factors was performed with the self-written RDC module[12] of the hotFCHT[13, 14] software package. The software calculates the order tensor in analogy to the SVD method proposed by Losonczi *et al.*[15] Using the order tensor, a set of RDCs (D_{calc}) is back-calculated and compared to the experimental values (D_{exp}) to give quality factors describing the validity of the structure model,[16] and additional parameters derived from the order tensor describing the alignment properties.[17]

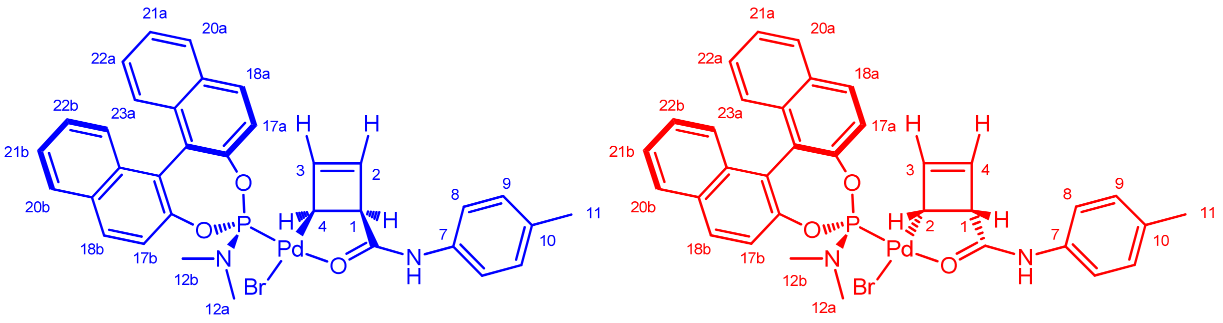

7a **7b**

Figure SI-6: Atom numbering for complexes **7a** and **7b** used for RDC analysis.

Table SI-5: Chemical shifts and assignments of ^{1}H and ^{13}C spectra recorded in an anisotropically swollen PDMS-gel with 2.5% of cross-linker, THF-*d8*, at 293K. $^{1}J_{CH}$ and $^{1}T_{CH}$ couplings were determined from ω_1-coupled HSQC experiments.[11] RDCs are then calculated according to $D = (T-J)/2$.

		^{1}H (ppm)	^{13}C (ppm)	$^{1}J_{CH}$ (Hz)	$^{1}T_{CH}$ (Hz)	RDC (Hz)
11	CH_3	2.28	20.82	125.7	129.0	1.65
12	NMe_2-1_a	2.78	38.45	138.1	140.5	2.4
12	NMe_2-2_a	2.80	38.52	=[4]	=	=
12	NMe_2-1_b	2.81	38.52	=	=	=
12	NMe_2-2_b	2.83	38.59	=	=	=
4	*c*B-4_b	3.58	50.25	156.5	151.8	–2.4
4	*c*B-4_a	3.89	49.01	155.6	165.7	5.05
1	*c*B-1_b	3.95	61.63	142.1	150.7	4.3
1	*c*B-1_a	3.98	61.27	141.96	150.8	4.4
3	*c*B-3_a	4.93	150.61	173.1	158.9	–7.1
2	*c*B-2_a	5.41	126.5	177.8	182.3	2.26
2	*c*B-2_b	5.86	126.7	177.2	173.1	–2.1
3	*c*B-3_b	6.67	151.21	170.5	171.2	0.37
9	*m*Tol	7.09-7.12	129.94	157.4	154.5	–1.5
17b	3-BNc_a	7.47	121.98	n/a[5]	166.6	n/a
17b	3-BNc_b	7.48	122.04	n/a	166.3	n/a
8	*o*Tol	7.52<br>7.54	120.62<br>120.64	162.0	158.6	–1.7
18b	4-BNc_{ab}	8.03	131.11<br>131.14	161.6	169.4	3.9
18a	4-BNn_b	8.06	131.17	162.0	168.2	3.1
18a	4-BNn_a	8.12	131.31	162.1	167.95	2.9
17a	3-BNn_a	8.32	124.4	167.8	163.8	–2.0

[4] $^{1}J_{(C,H)}$ and $^{1}T_{(C,H)}$ were not extracted, as the fitting of the corresponding RDCs would not be reasonable.
[5] $^{1}J_{(C,H)}$ weren't extracted due to partial signal overlap.

17a	3-BNn$_b$	8.33	124.2	167.6	164.05	–1.80
-	CONH$_a$	9.96	181.95	-	-	-
-	CONH$_b$	9.98	181.77	-	-	-

The RDC of the methyl group (C11 in Figure SI-6) is converted to the corresponding C–C RDC according to Verdier *et al.*[18] and used in the fitting process with the same weight as the C–H-RDCs. Other RDCs of the tolyl- and the NMe_2-fragments have not been used. Table SI- gives the details of the RDC fits of the experimental data to the calculated structures. Experimental errors for the RDCs are estimated based on the lineshape and signal/noise ratio of the analyzed peaks in the traces of the ω_1-coupled HSQC as well as the observed differences in the (as defined by the geometry of the MONOPHOS ligand) parallel RDCs of C18a–H18a and C18b–H18b (see Figure SI-6). A graphical representation of the correlation of experimental and calculated RDC values for all combinations of fits of RDC data sets to the proposed geometries is given in Figure SI-7. Full details of the fitting results are given for the two best-fitting data sets in Table SI-6, supporting the proposed conformation of the complexes **7a** and **7b** in solution.

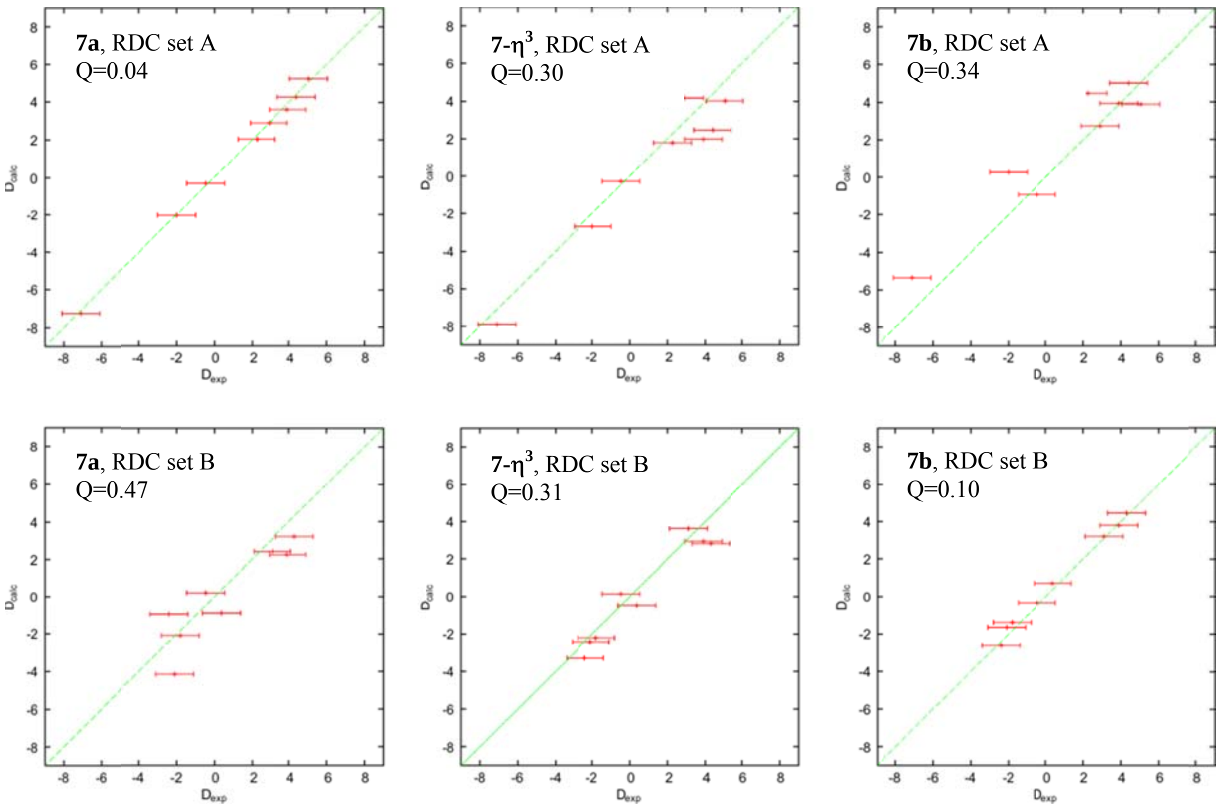

Figure SI-7: Correlation of experimental and back-calculated RDCs of **7a** and **7b**. Both fits show an excellent correlation and support the proposed conformation of the complexes. Details of the RDC fit are given in Table SI-**6**.

Table SI-6: RDC fits of **7a** and **7b**. Experimental and back-calculated RDC values as well as parameters describing the goodness-of-fit and the order tensor properties.

	RDC set A / complex **7a**				RDC set B / complex **7b**			
RDC	D_{exp} (Hz)		D_{calc} (Hz)		D_{exp} (Hz)		D_{calc} (Hz)	
C1 – H1	4.40	±1.00	4.28	±1.54	4.30	±1.00	4.45	±1.20
C2 – H2	2.26	±1.00	2.03	±0.84	-2.10	±1.00	-1.64	±0.64
C3 – H3	-7.10	±1.00	-7.24	±0.55	0.37	±1.00	0.71	±2.52
C4 – H4	5.05	±1.00	5.23	±1.30	-2.40	±1.00	-2.61	±0.04
C10 – C11	-0.47	±1.00	-0.33	±0.16	-0.47	±1.00	-0.32	±0.17
C17a – H17a	-2.00	±1.00	-2.03	±0.92	-1.80	±1.00	-1.37	±0.90
C18a – H18a	2.90	±1.00	2.90	±0.71	3.10	±1.00	3.20	±1.00
C18b – H18b	3.90	±1.00	3.61	±0.65	3.90	±1.00	3.79	±1.02
Quality Factor	Q		0.042				0.105	
Condition Number	cond.		4.76				5.34	
Tensor Magnitude	GDO (*10^{-4})		4.93				5.48	
Axial Component	D_a (*10^{-4})		2.36				2.60	
Rhombic Component	D_r (*10^{-4})		0.81				0.98	
Tensor Orientation	α (°)		67.81				126.01	
—	β (°)		11.01				168.36	
Euler Angles	γ (°)		166.74				2.17	

15. Investigation of the Aggregation Behavior

X-Ray. Intermolecular distances between molecules are evaluated. The short Br-H(N) distance is shown as a dashed line in Figure SI-8, indicating an H-bond.

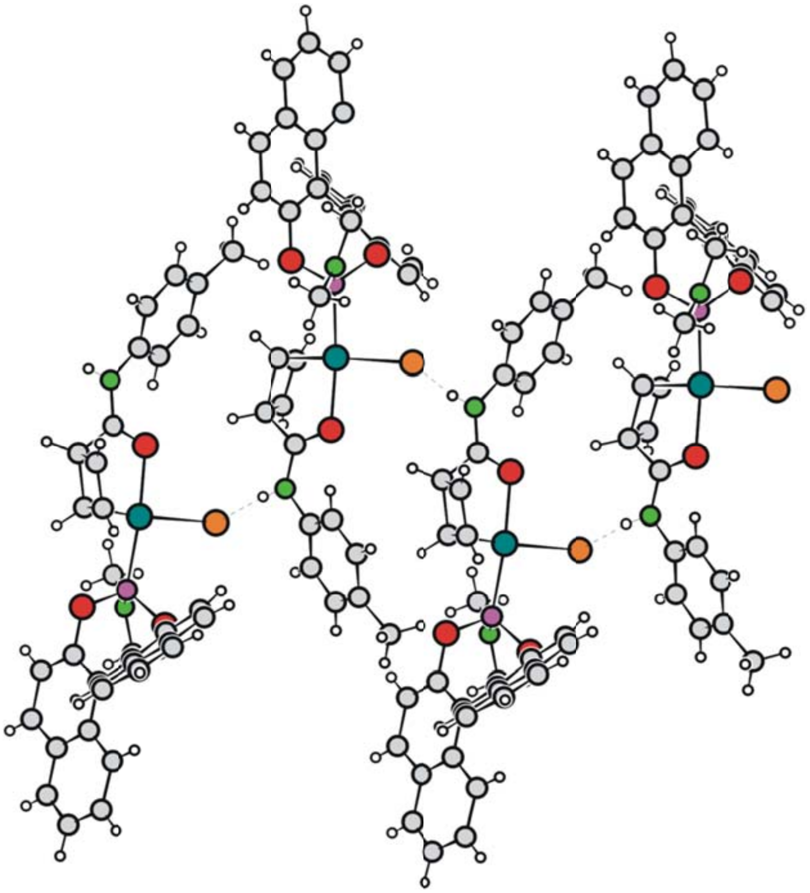

Figure SI-8: Four molecules of 7a, as measured by X-ray diffraction. The intermolecular Br-H(N) distances (represented by dashed lines from orange → white → green) are fairly short, indicative of an attractive H-bond.

Concentration dependence of chemical shifts. As the NMR chemical shift is well-known to show differences in the case of aggregation, we also acquired ^{1}H (Figure SI-9) and ^{31}P spectra (Figure SI-10) of the isomeric mixture of **7a/b** at 0.1 M and 0.01 M concentrations. Multiple signals show a pronounced positive and negative differences in chemical shift between the two concentrations. This strongly indicates aggregation in solution.

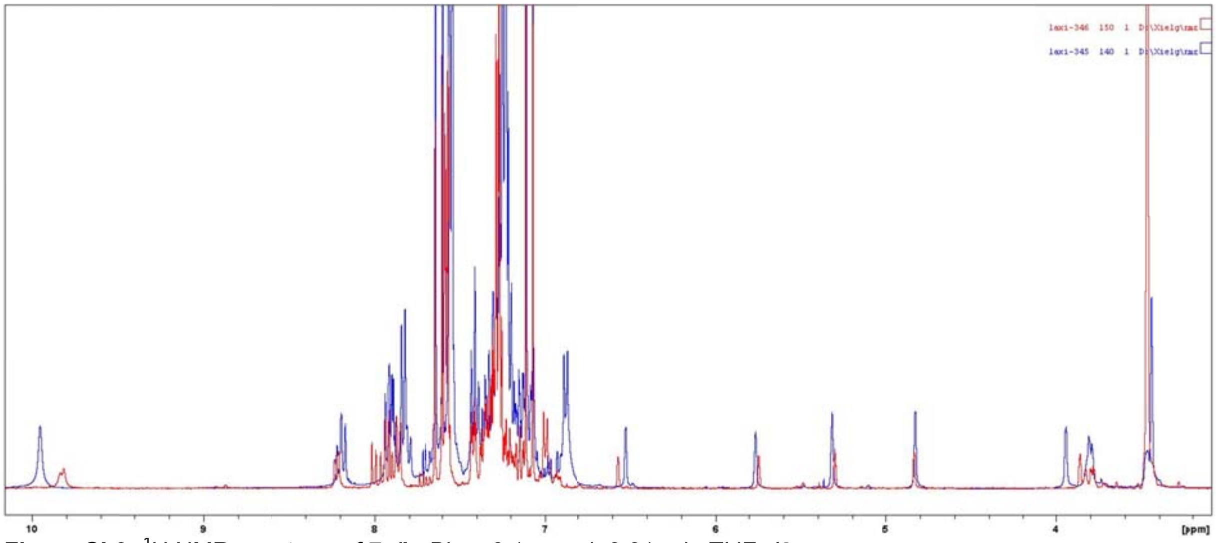

Figure SI-9: ^{1}H NMR spectrum of **7a/b**. Blue: 0.1 M, red: 0.01 M in THF-*d8*.

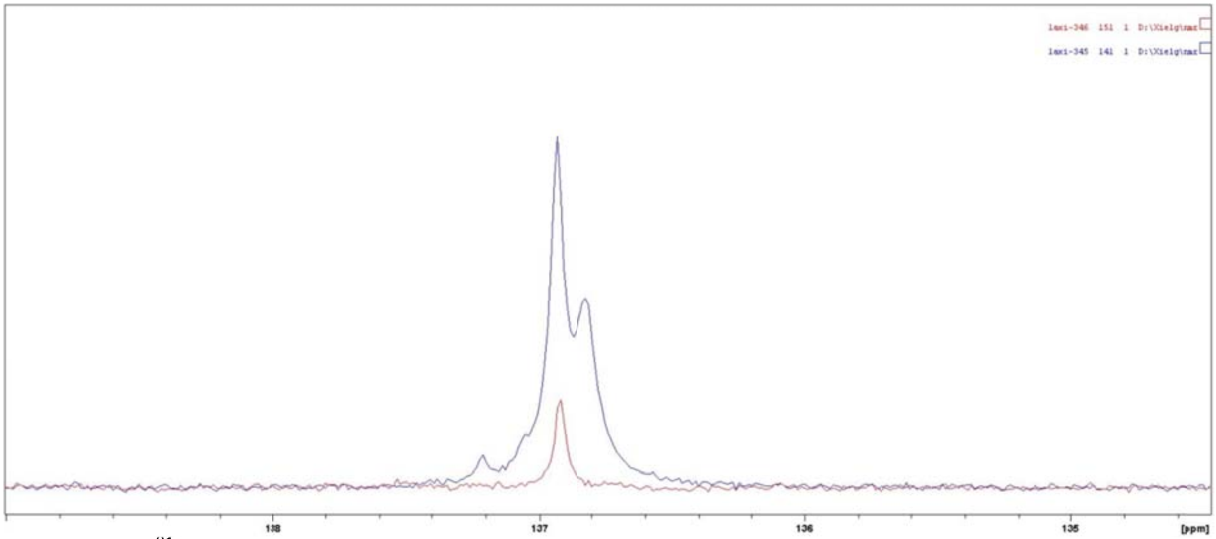

Figure SI-10: ^{31}P NMR spectrum of **7a/b**. Blue: 0.1 M, red: 0.01 M in THF-*d8*.

16. Facial exchange of complex 5 in presence of AgOTf and additives

See Figure **4** of the main text and Spectra on pages 51-54.

A) 7.1 × 10^{-2} M

In a flame dried schlenk flask, under Argon atmosphere, $Pd(dba)_2$ (28.8 mg, 0.05 mmol), (*R*)-Monophos (35.9 mg, 0.1 mmol) and *cis*-chlorocarboxylic amide **3** (11.1 mg, 0.05 mmol) were added. After three vacuum-Argon cycles, 0.7 mL THF-d_8 was added. The solution was stirred at room temperature for 20 minutes, following by the addition of the corresponding amount of additive and AgOTf (25.7 mg, 0.1 mmol). The mixture was immediately transferred to a NMR tube, sealed and fast ^{1}H NMR analyzed at 298 K in 5 minutes and then every 10 minutes.

B) 1.4 × 10^{-2} M and additives

In a flame dried schlenk flask, under Argon atmosphere, $Pd(dba)_2$ (5.75 mg, 0.01 mmol), (*R*)-Monophos (7.19 mg, 0.02 mmol) and *cis*-chlorocarboxylic amide **3** (2.22 mg, 0.01 mmol) were added. After three vacuum-Argon cycles, 0.7 mL THF-d_8 was added. The solution was stirred at room temperature for 20 minutes, following by the addition of the corresponding amount of additive and AgOTf (5.14 mg, 0.02 mmol). The mixture was immediately transferred to a NMR tube, sealed and fast ^{1}H NMR analyzed at 298 K every 10 minutes (~30 minutes after 1h).

References

1. C. M. Thiele, K. Petzold and J. Schleucher, *Chem. Eur. J.*, 2009, **15**, 585-588.
2. H. Kessler, H. Oschkinat, C. Griesinger and W. Bermel, *J. Magn. Reson.*, 1986, **70**, 106-133.
3. J. Stonehouse, P. Adell, J. Keeler and A. J. Shaka, *J. Am. Chem. Soc.*, 1994, **116**, 6037-6038.
4. K. Stott, J. Stonehouse, J. Keeler, T. L. Hwang and A. J. Shaka, *J. Am. Chem. Soc.*, 1995, **117**, 4199-4200.
5. M. J. Thrippleton and J. Keeler, *Angew. Chem. Int. Ed.*, 2003, **42**, 3938-3941.
6. S. Macura, B. T. Farmer and L. R. Brown, *J. Magn. Reson.*, 1986, **70**, 493-499.
7. F. Neese, *Wiley Interdiscip. Rev.: Comput. Mol. Sci.*, 2012, **2**, 73-78.
8. F. Weigend and R. Ahlrichs, *Phys. Chem. Chem. Phys.*, 2005, **7**, 3297-3305.
9. D. Andrae, Diploma thesis, unpublished, 1989.
10. A. Bergner, M. Dolg, W. Kuchle, H. Stoll and H. Preuss, *Mol. Phys.*, 1993, **80**, 1431-1441.
11. C. M. Thiele and W. Bermel, *J. Magn. Reson.*, 2012, **216**, 134-143.
12. V. Schmidts, PhD thesis, Technische Universität Darmstadt, Darmstadt, 2013.
13. R. Berger, C. Fischer and M. Klessinger, *J. Phys. Chem. A*, 1998, **102**, 7157-7167.

14. H. C. Jankowiak, J. L. Stuber and R. Berger, *J. Chem. Phys.*, 2007, **127**.
15. J. A. Losonczi, M. Andrec, M. W. F. Fischer and J. H. Prestegard, *J. Magn. Reson.*, 1999, **138**, 334-342.
16. G. Cornilescu, J. L. Marquardt, M. Ottiger and A. Bax, *J. Am. Chem. Soc.*, 1998, **120**, 6836-6837.
17. M. Zweckstetter, *Nat. Protoc.*, 2008, **3**, 679-690.
18. L. Verdier, P. Sakhaii, M. Zweckstetter and C. Griesinger, *J. Magn. Reson.*, 2003, **163**, 353-359.

NMR and HPLC

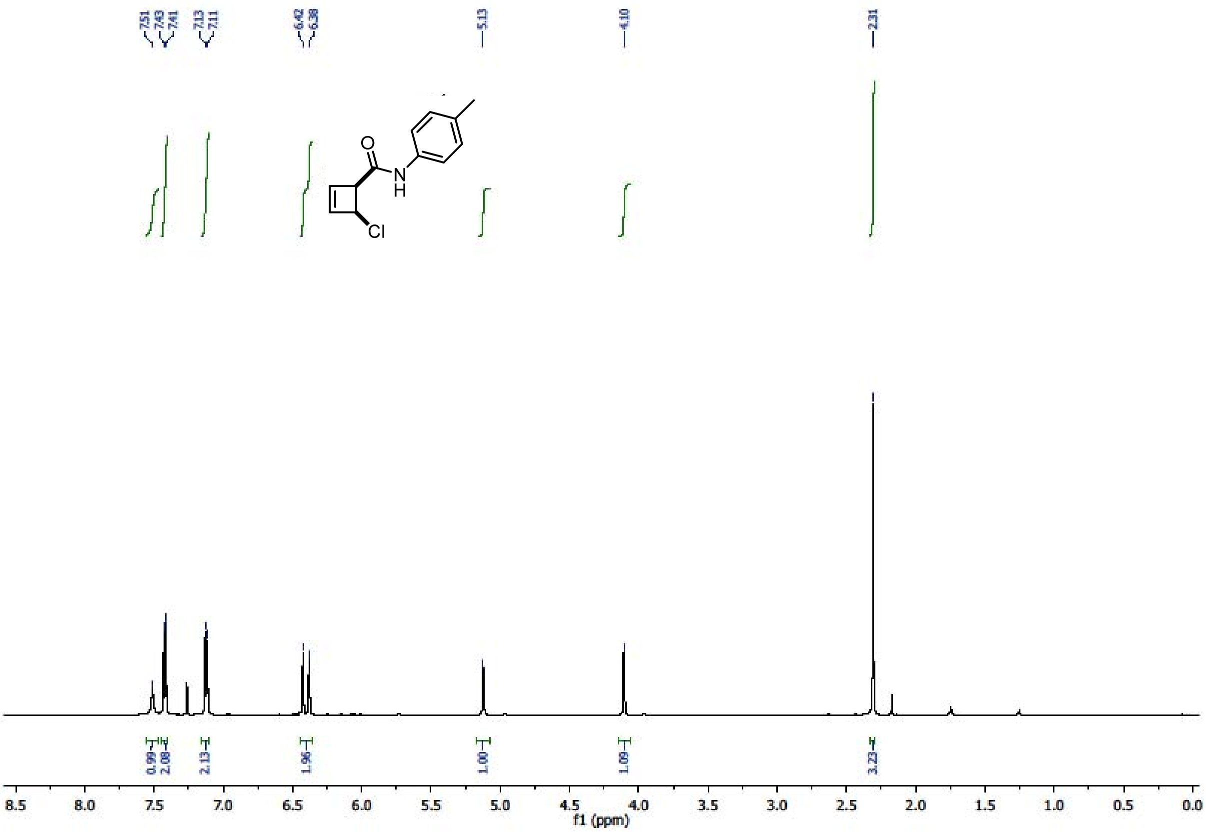

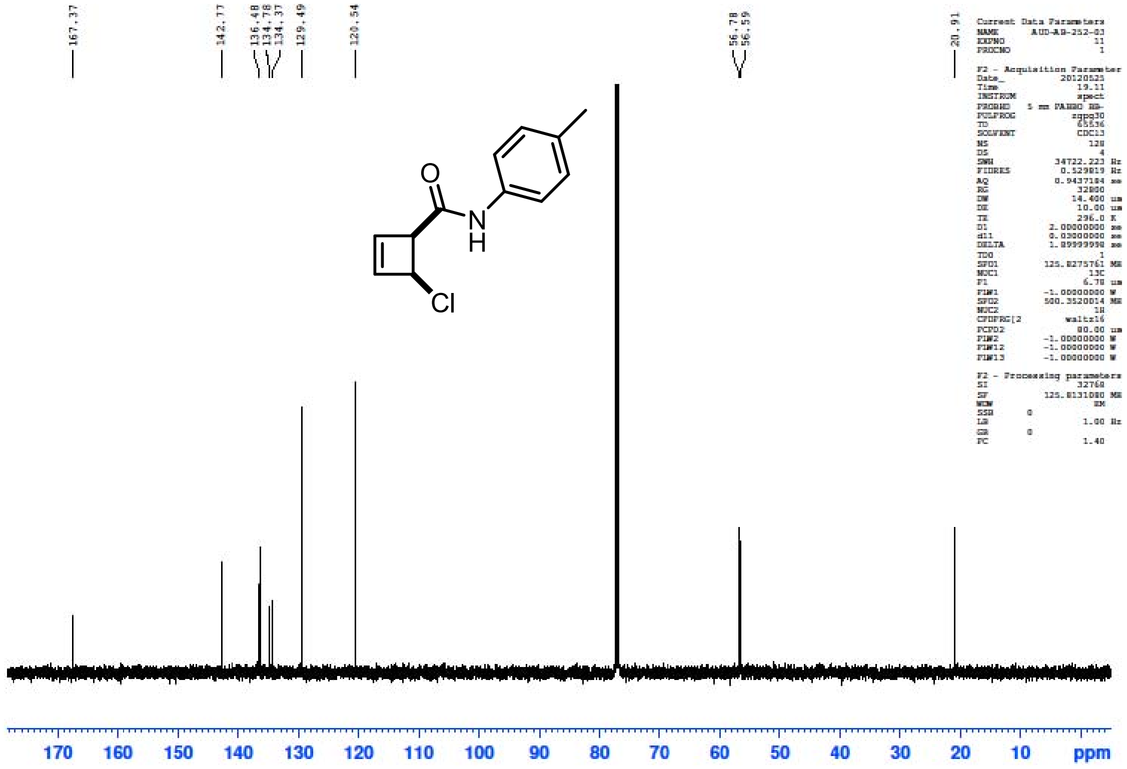

167.37
142.77
136.48
134.78
134.37
129.49
120.54
56.78
56.59
20.91
O
N
H
Cl
Current Data Parameters
NAME AUD-AB-252-03
EXPNO 11
PROCNO 1
F2 - Acquisition Parameter
Date_ 20120525
Time 19.11
INSTRUM spect
PROBHD 5 mm PABBO BB-
PULPROG zgpg30
TD 65536
SOLVENT CDCl3
NS 128
DS 4
SWH 34722.223 Hz
FIDRES 0.529819 Hz
RG 32800
DW 14.400
DE 10.00
TE 296.0 K
TD0 1
NUC1 13C
F1 6.78
NUC2 1H
CPDPRG[2 waltz16
PCPD2 80.00
F2 - Processing parameters
SI 32768
WDW EM
SSB 0
LB 1.00 Hz
GB 0
PC 1.40
170 160 150 140 130 120 110 100 90 80 70 60 50 40 30 20 10 ppm

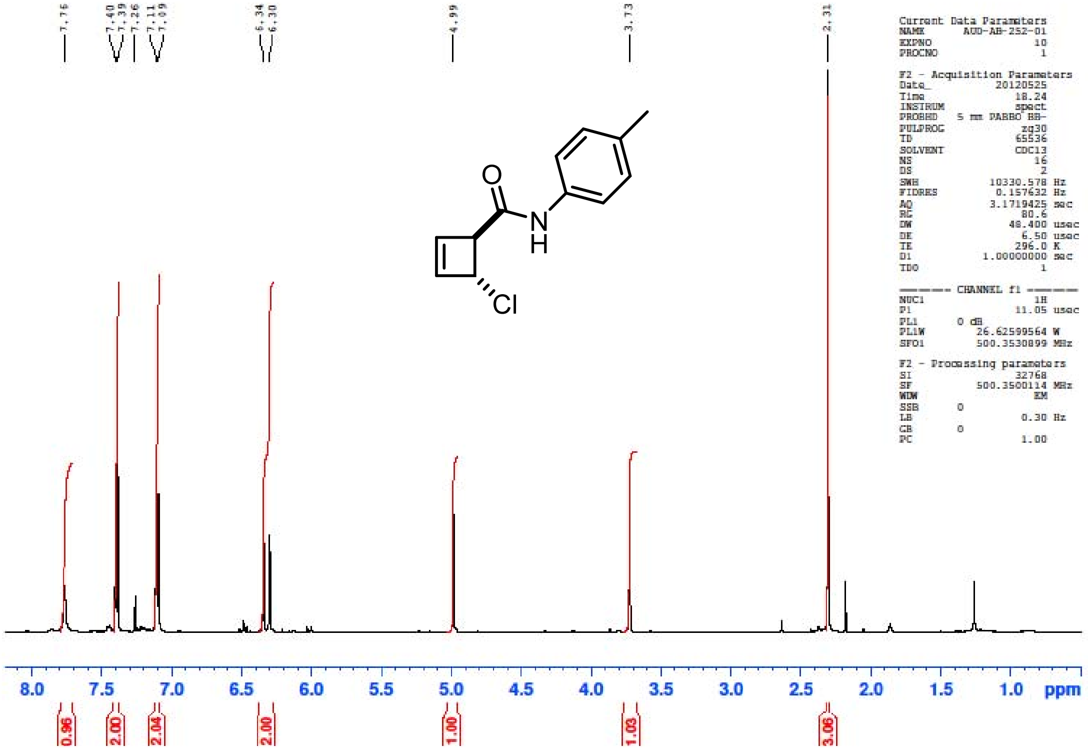

7.76
7.40
7.39
7.26
7.11
7.09
6.34
6.30
4.99
3.73
2.31
O
N
H
Cl
Current Data Parameters
NAME AUD-AB-252-01
EXPNO 10
PROCNO 1
F2 - Acquisition Parameters
Date_ 20120525
Time 18.24
INSTRUM spect
PROBHD 5 mm PABBO BB-
PULPROG zg30
TD 65536
SOLVENT CDCl3
NS 16
DS 2
SWH 10330.578 Hz
FIDRES 0.157632 Hz
AQ 3.1719425 sec
RG 80.6
DW 48.400 usec
DE 6.50 usec
TE 296.0 K
D1 1.00000000 sec
TD0 1
======== CHANNEL f1 ========
NUC1 1H
P1 11.05 usec
PL1 0 dB
PL1W 26.62599564 W
SFO1 500.3530899 MHz
F2 - Processing parameters
SI 32768
SF 500.3500114 MHz
WDW EM
SSB 0
LB 0.30 Hz
GB 0
PC 1.00
8.0 7.5 7.0 6.5 6.0 5.5 5.0 4.5 4.0 3.5 3.0 2.5 2.0 1.5 1.0 ppm
0.96
2.00
2.04
2.00
1.00
1.03
3.06

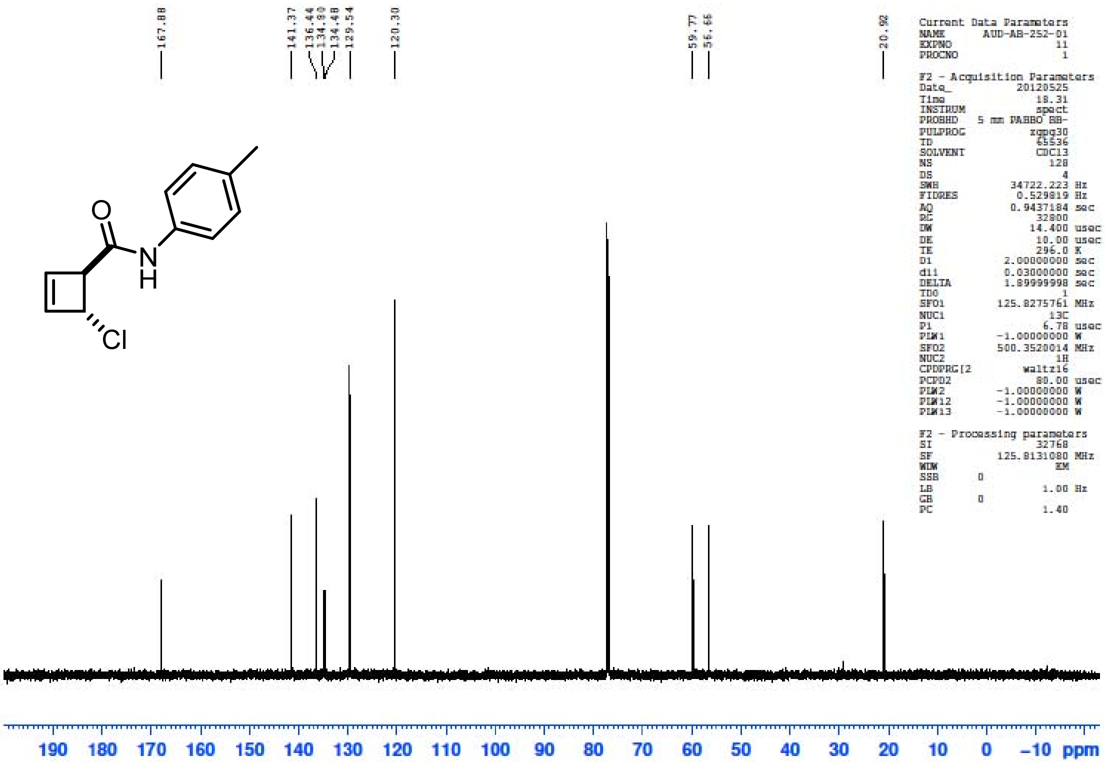

167.88
141.37
136.44
134.90
134.48
129.54
120.30
59.77
56.66
20.92
O
N
H
Cl
190 180 170 160 150 140 130 120 110 100 90 80 70 60 50 40 30 20 10 0 −10 ppm

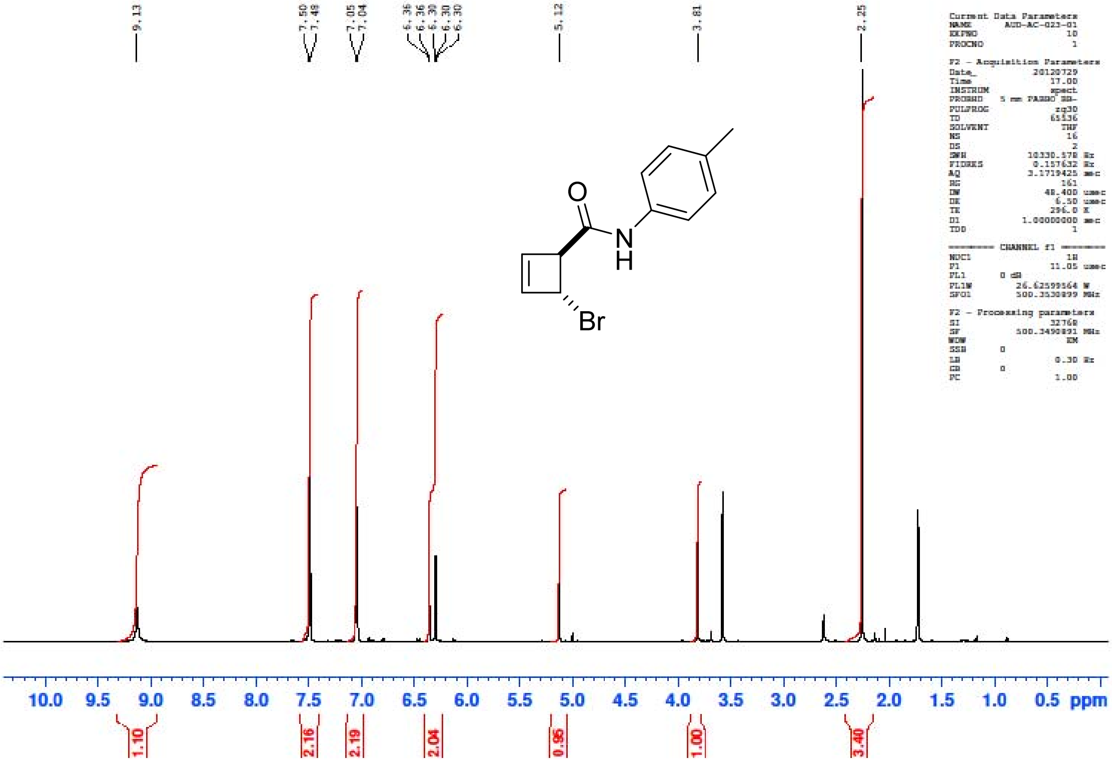

9.13
7.50
7.49
7.05
7.04
6.36
6.36
6.30
6.30
6.30
5.12
3.81
2.25
O
N
H
Br
10.0 9.5 9.0 8.5 8.0 7.5 7.0 6.5 6.0 5.5 5.0 4.5 4.0 3.5 3.0 2.5 2.0 1.5 1.0 0.5 ppm
1.10
2.16
2.19
2.04
0.95
1.00
3.40

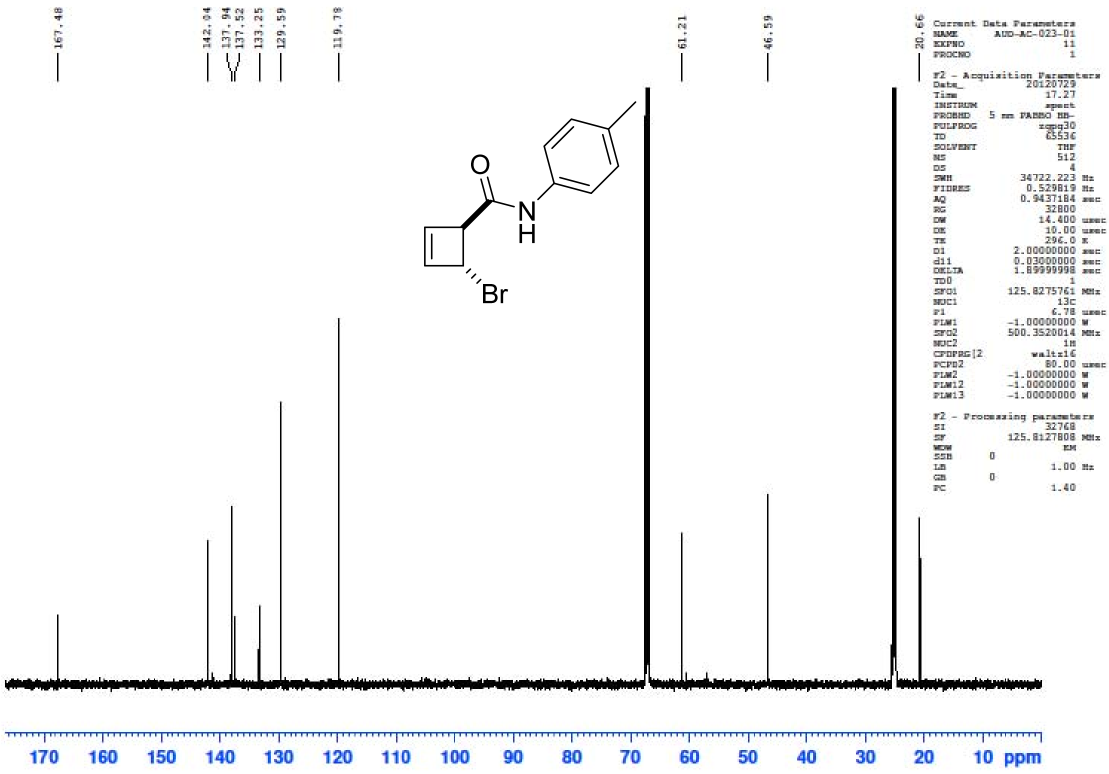
167.48
142.04
137.94
137.52
133.25
129.59
119.79
61.21
46.59
20.66
Br
O
N
H
Current Data Parameters
NAME AUD-AC-023-01
EXPNO 11
PROCNO 1
F2 - Acquisition Parameters
Date_ 20120729
Time 17.27
INSTRUM spect
PROBHD 5 mm PABBO BB-
PULPROG zgpg30
TD 65536
SOLVENT THF
NS 512
DS 4
SWH 34722.223 Hz
FIDRES 0.529819 Hz
AQ 0.9437184 sec
RG 32800
DW 14.400 usec
DE 10.00 usec
TE 296.0 K
D1 2.00000000 sec
d11 0.03000000 sec
DELTA 1.89999998 sec
TD0 1
SFO1 125.8275761 MHz
NUC1 13C
P1 6.78 usec
PLW1 -1.00000000 W
SFO2 500.3520014 MHz
NUC2 1H
CPDPRG[2 waltz16
PCPD2 80.00 usec
PLW2 -1.00000000 W
PLW12 -1.00000000 W
PLW13 -1.00000000 W
F2 - Processing parameters
SI 32768
SF 125.8127808 MHz
WDW EM
SSB 0
LB 1.00 Hz
GB 0
PC 1.40
170 160 150 140 130 120 110 100 90 80 70 60 50 40 30 20 10 ppm

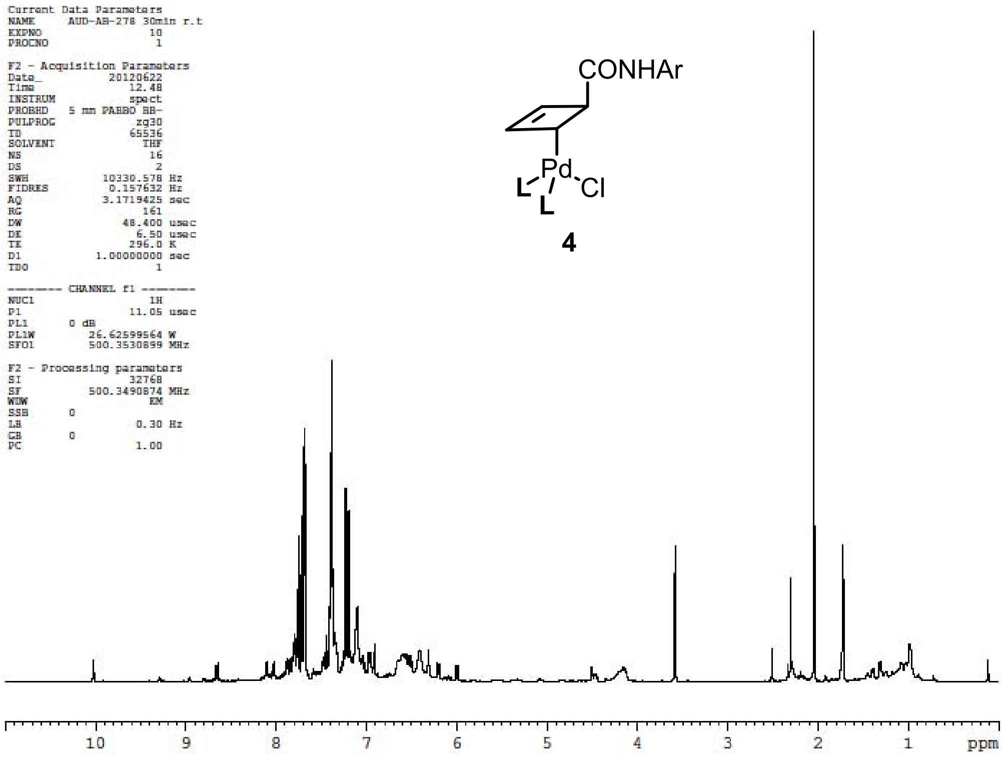
Current Data Parameters
NAME AUD-AB-278 30min r.t
EXPNO 10
PROCNO 1
F2 - Acquisition Parameters
Date_ 20120622
Time 12.48
INSTRUM spect
PROBHD 5 mm PABBO BB-
PULPROG zg30
TD 65536
SOLVENT THF
NS 16
DS 2
SWH 10330.578 Hz
FIDRES 0.157632 Hz
AQ 3.1719425 sec
RG 161
DW 48.400 usec
DE 6.50 usec
TE 296.0 K
D1 1.00000000 sec
TD0 1
======== CHANNEL f1 ========
NUC1 1H
P1 11.05 usec
PL1 0 dB
PL1W 26.62599564 W
SFO1 500.3530899 MHz
F2 - Processing parameters
SI 32768
SF 500.3490874 MHz
WDW EM
SSB 0
LB 0.30 Hz
GB 0
PC 1.00
CONHAr
Pd
Cl
L
L
4
10 9 8 7 6 5 4 3 2 1 ppm

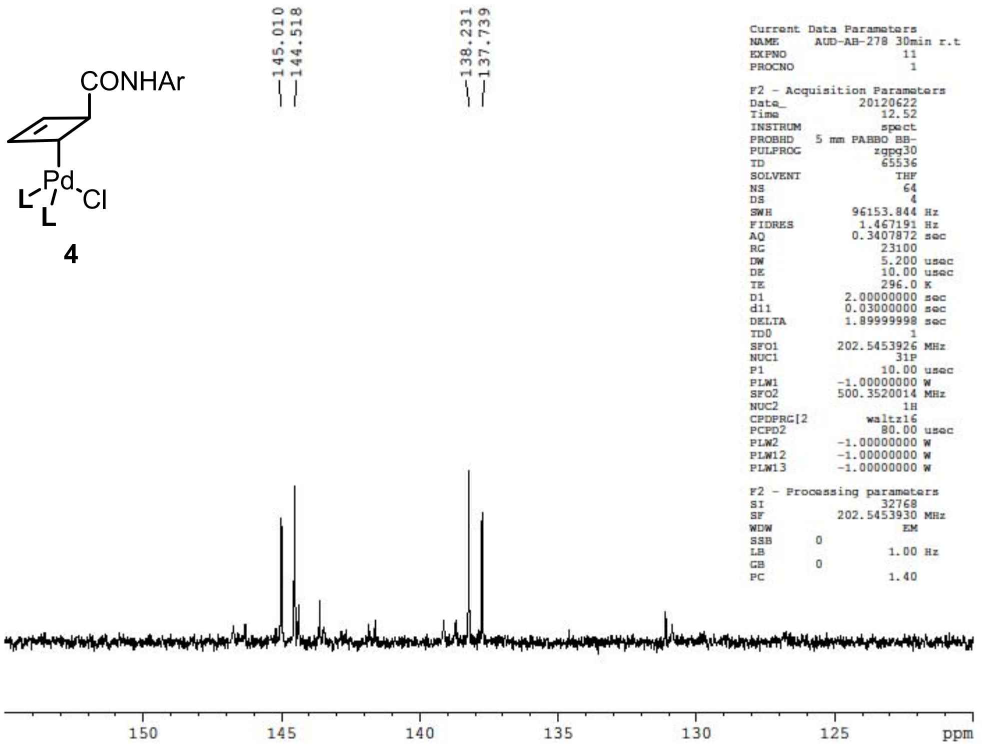
CONHAr
Pd
L
L
Cl
4
145.010
144.518
138.231
137.739
Current Data Parameters
NAME AUD-AB-278 30min r.t
EXPNO 11
PROCNO 1
F2 - Acquisition Parameters
Date_ 20120622
Time 12.52
INSTRUM spect
PROBHD 5 mm PABBO BB-
PULPROG zgpg30
TD 65536
SOLVENT THF
NS 64
DS 4
SWH 96153.844 Hz
FIDRES 1.467191 Hz
AQ 0.3407872 sec
RG 23100
DW 5.200 usec
DE 10.00 usec
TE 296.0 K
D1 2.00000000 sec
d11 0.03000000 sec
DELTA 1.89999998 sec
TD0 1
SFO1 202.5453926 MHz
NUC1 31P
P1 10.00 usec
PLW1 -1.00000000 W
SFO2 500.3520014 MHz
NUC2 1H
CPDPRG[2 waltz16
PCPD2 80.00 usec
PLW2 -1.00000000 W
PLW12 -1.00000000 W
PLW13 -1.00000000 W
F2 - Processing parameters
SI 32768
SF 202.5453930 MHz
WDW EM
SSB 0
LB 1.00 Hz
GB 0
PC 1.40
150
145
140
135
130
125
ppm

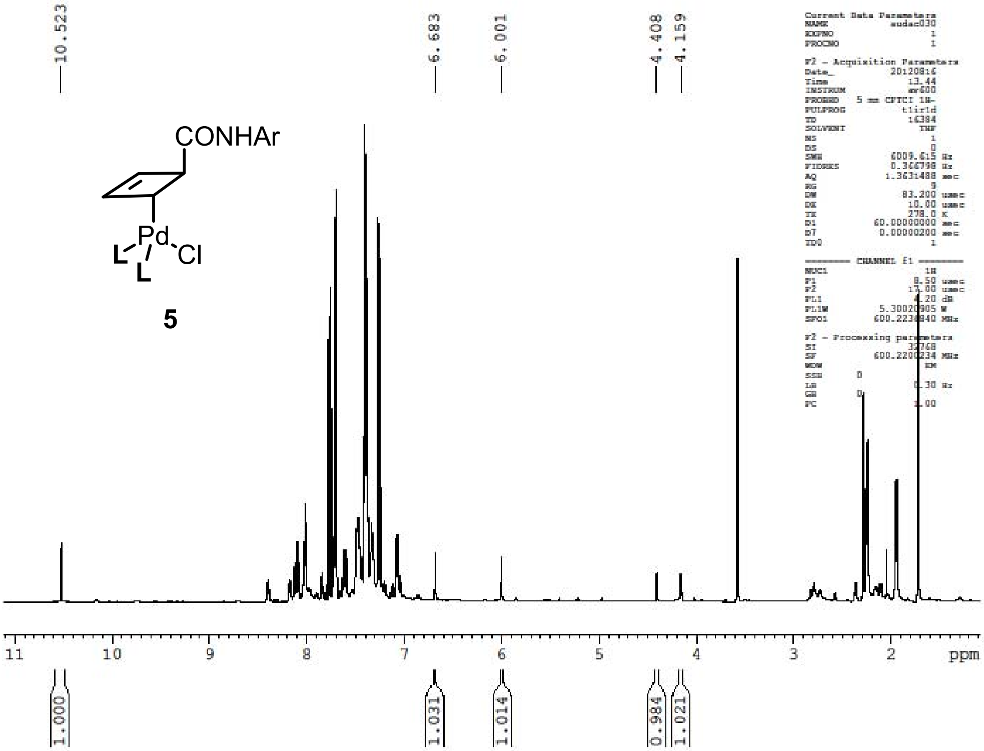
CONHAr
Pd
L
L
Cl
5
10.523
6.683
6.001
4.408
4.159
Current Data Parameters
F2 - Acquisition Parameters
CHANNEL f1
F2 - Processing parameters
11
10
9
8
7
6
5
4
3
2
ppm
1.000
1.031
1.014
0.984
1.021

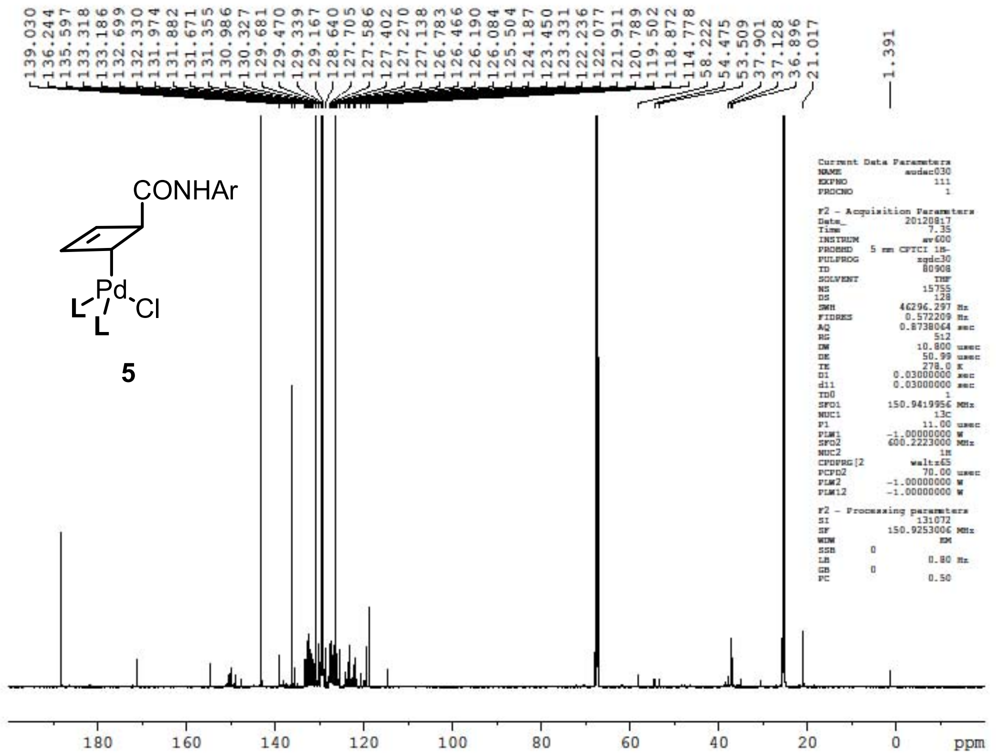
139.030
136.244
135.597
133.318
133.186
132.699
132.330
131.974
131.882
131.671
131.355
130.986
130.327
129.681
129.470
129.339
129.167
128.640
127.705
127.586
127.402
127.270
127.138
126.783
126.466
126.190
126.084
125.504
124.187
123.450
123.331
122.236
122.077
121.911
120.789
119.502
118.872
114.778
58.222
54.475
53.509
37.901
37.128
36.896
21.017
1.391
CONHAr
Pd
L
L
Cl
5
Current Data Parameters
NAME audac030
EXPNO 111
PROCNO 1
F2 - Acquisition Parameters
Date_ 20120817
Time 7.35
INSTRUM av600
PULPROG zgdc30
TD 80906
SOLVENT THF
NS 15755
DS 128
SWH 46296.297 Hz
FIDRES 0.572209 Hz
AQ 0.8738064 sec
RG 512
DW 10.800 usec
DE 50.99 usec
TE 278.0 K
D1 0.03000000 sec
d11 0.03000000 sec
TD0 1
SFO1 150.9419956 MHz
NUC1 13C
P1 11.00 usec
PLW1 -1.00000000 W
SFO2 600.2223000 MHz
NUC2 1H
CPDPRG[2 waltz65
PCPD2 70.00 usec
PLW2 -1.00000000 W
PLW12 -1.00000000 W
F2 - Processing parameters
SI 131072
SF 150.9253006 MHz
WDW EM
SSB 0
LB 0.80 Hz
GB 0
PC 0.50
180
160
140
120
100
80
60
40
20
0
ppm

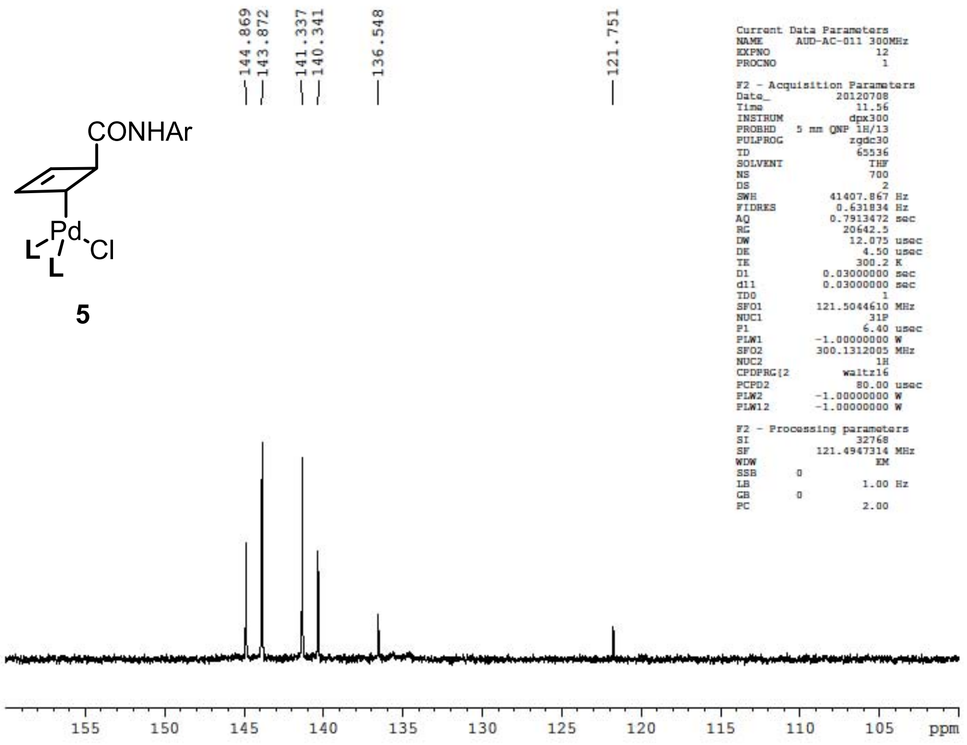
144.869
143.872
141.337
140.341
136.548
121.751
CONHAr
Pd
L
L
Cl
5
Current Data Parameters
NAME AUD-AC-011 300MHz
EXPNO 12
PROCNO 1
F2 - Acquisition Parameters
Date_ 20120708
Time 11.56
INSTRUM dpx300
PROBHD 5 mm QNP 1H/13
PULPROG zgdc30
TD 65536
SOLVENT THF
NS 700
DS 2
SWH 41407.867 Hz
FIDRES 0.631834 Hz
AQ 0.7913472 sec
RG 20642.5
DW 12.075 usec
DE 4.50 usec
TE 300.2 K
D1 0.03000000 sec
d11 0.03000000 sec
TD0 1
SFO1 121.5044610 MHz
NUC1 31P
P1 6.40 usec
PLW1 -1.00000000 W
SFO2 300.1312005 MHz
NUC2 1H
CPDPRG[2 waltz16
PCPD2 80.00 usec
PLW2 -1.00000000 W
PLW12 -1.00000000 W
F2 - Processing parameters
SI 32768
SF 121.4947314 MHz
WDW EM
SSB 0
LB 1.00 Hz
GB 0
PC 2.00
155
150
145
140
135
130
125
120
115
110
105
ppm

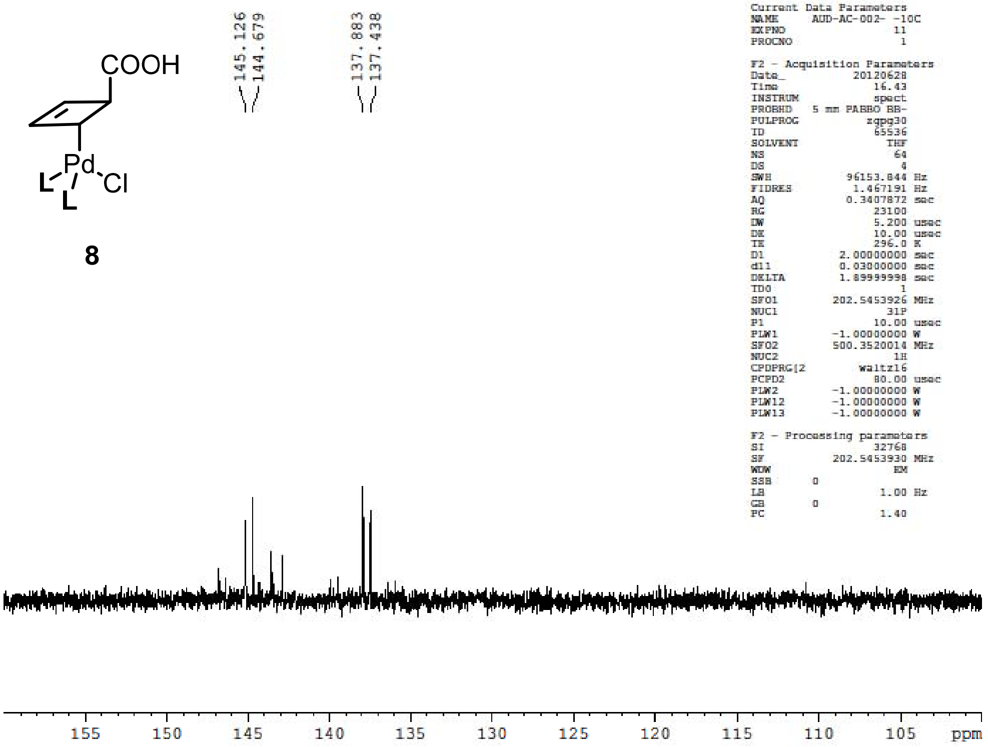
COOH
Pd
L
L
Cl
8
145.126
144.679
137.883
137.438
Current Data Parameters
NAME AUD-AC-002- -10C
EXPNO 11
PROCNO 1
F2 - Acquisition Parameters
Date_ 20120628
Time 16.43
INSTRUM spect
PROBHD 5 mm PABBO BB-
PULPROG zgpg30
TD 65536
SOLVENT THF
NS 64
DS 4
SWH 96153.844 Hz
FIDRES 1.467191 Hz
AQ 0.3407872 sec
RG 23100
DW 5.200 usec
DE 10.00 usec
TE 296.0 K
D1 2.00000000 sec
d11 0.03000000 sec
DELTA 1.89999998 sec
TD0 1
SFO1 202.5453926 MHz
NUC1 31P
P1 10.00 usec
PLW1 -1.00000000 W
SFO2 500.3520014 MHz
NUC2 1H
CPDPRG[2 waltz16
PCPD2 80.00 usec
PLW2 -1.00000000 W
PLW12 -1.00000000 W
PLW13 -1.00000000 W
F2 - Processing parameters
SI 32768
SF 202.5453930 MHz
WDW EM
SSB 0
LB 1.00 Hz
GB 0
PC 1.40
155 150 145 140 135 130 125 120 115 110 105 ppm

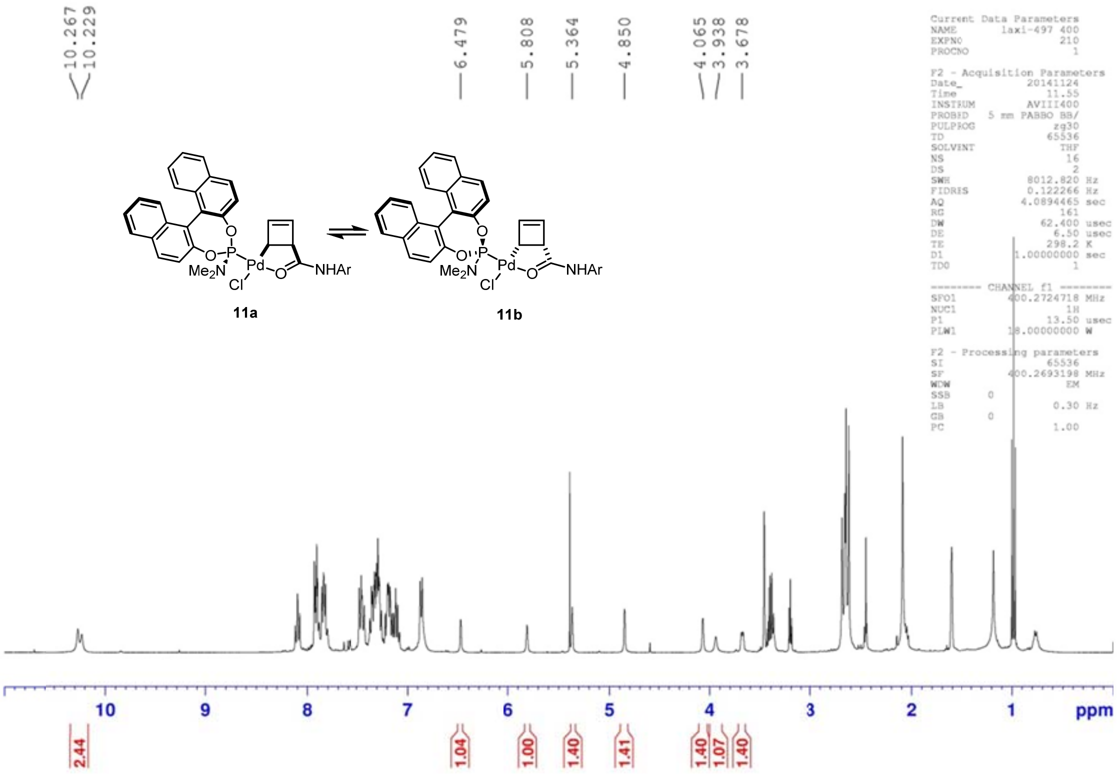
10.267
10.229
6.479
5.808
5.364
4.850
4.065
3.938
3.678
O
O
P
Pd
Me2N
Cl
O
NHAr
11a
11b
Current Data Parameters
NAME laxi-497 400
EXPNO 210
PROCNO 1
F2 - Acquisition Parameters
Date_ 20141124
Time 11.55
INSTRUM AVIII400
PROBHD 5 mm PABBO BB/
PULPROG zg30
TD 65536
SOLVENT THF
NS 16
DS 2
SWH 8012.820 Hz
FIDRES 0.122266 Hz
AQ 4.0894465 sec
RG 161
DW 62.400 usec
DE 6.50 usec
TE 298.2 K
D1 1.00000000 sec
TD0 1
======== CHANNEL f1 ========
SFO1 400.2724718 MHz
NUC1 1H
P1 13.50 usec
PLW1 18.00000000 W
F2 - Processing parameters
SI 65536
SF 400.2693198 MHz
WDW EM
SSB 0
LB 0.30 Hz
GB 0
PC 1.00
10 9 8 7 6 5 4 3 2 1 ppm
2.44
1.04
1.00
1.40
1.41
1.40
1.07
1.40

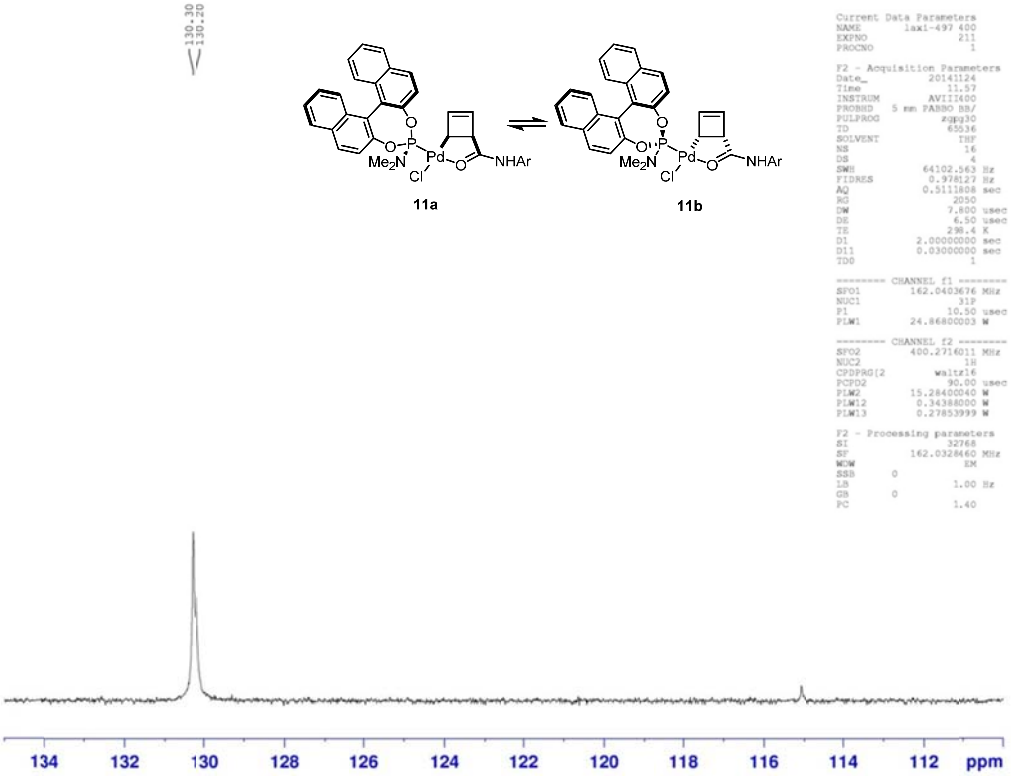
130.30
130.20
Pd
O
NHAr
Me2N
Cl
11a
11b
134
132
130
128
126
124
122
120
118
116
114
112
ppm

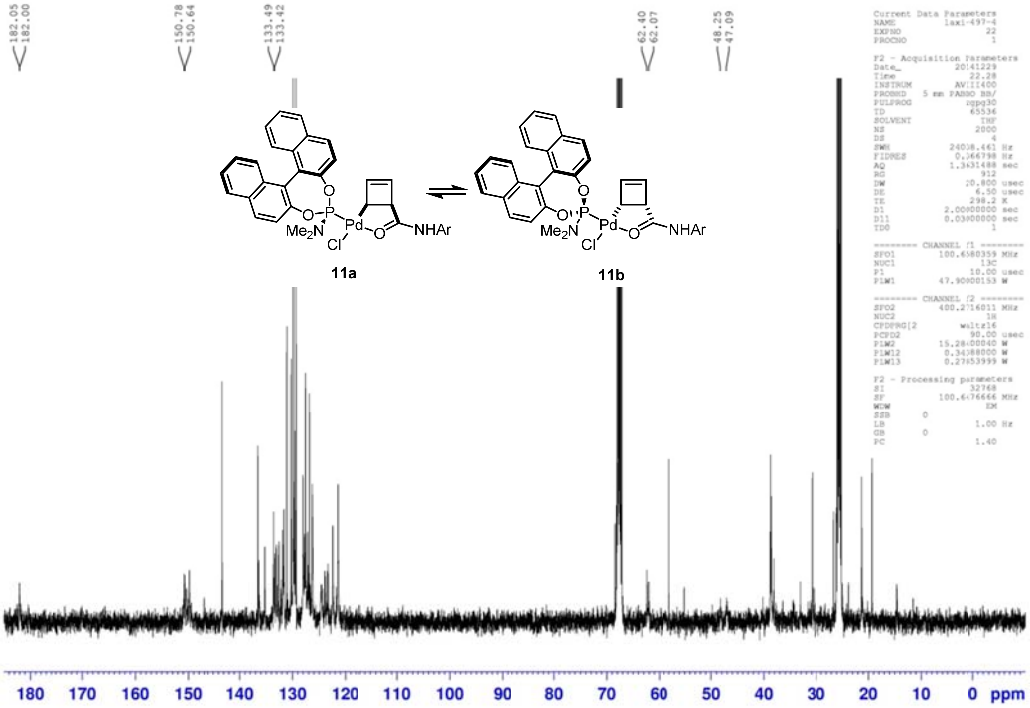
182.05
182.00
150.78
150.64
133.49
133.42
62.40
62.07
48.25
47.09
Pd
O
NHAr
Me2N
Cl
11a
11b
180
170
160
150
140
130
120
110
100
90
80
70
60
50
40
30
20
10
0
ppm

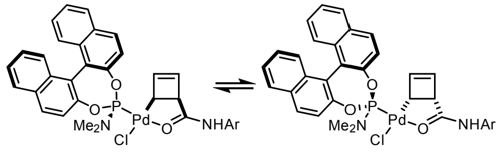

11a ^{1}H-^{1}H COSY **11b**

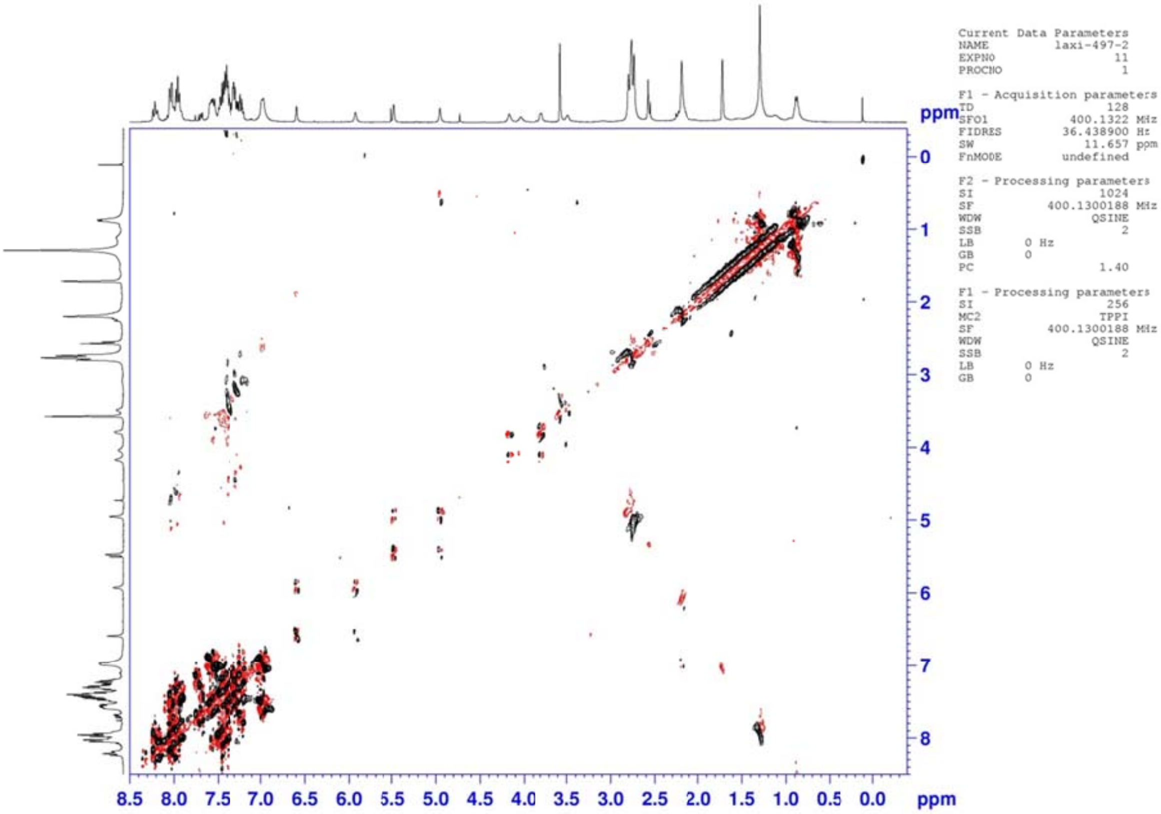

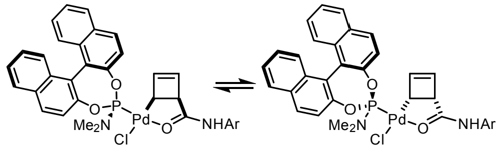

11a ^{1}H-^{13}C HSQC **11b**

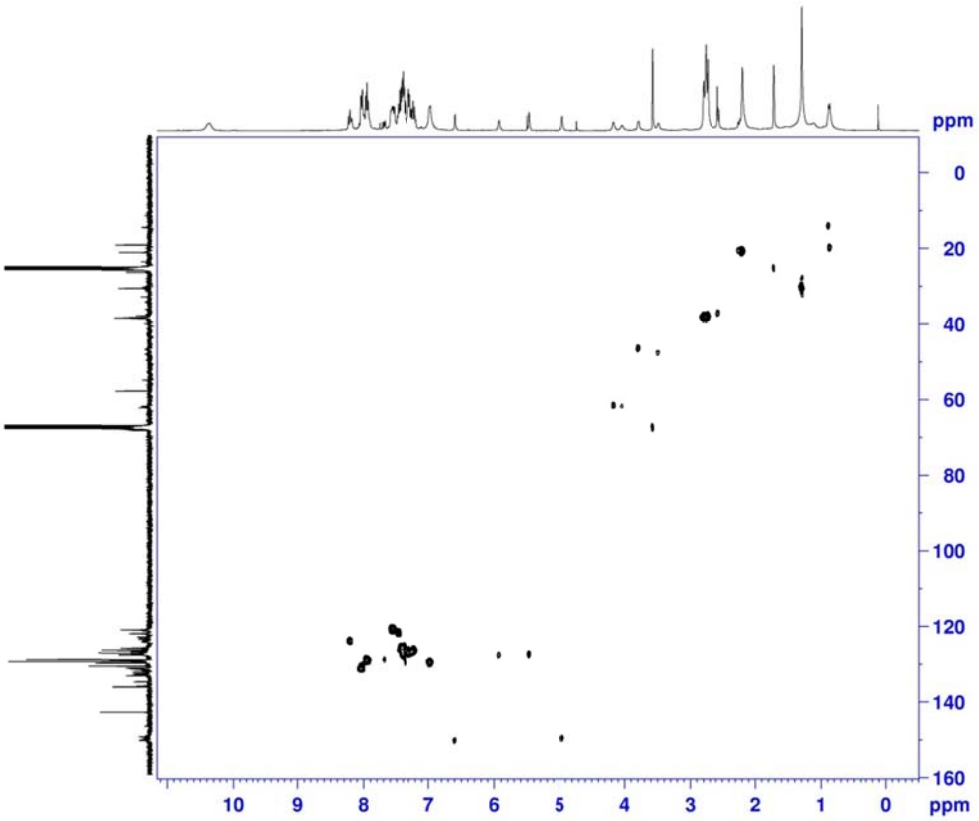


```
Current Data Parameters
NAME           laxi-497-2
EXPNO                  12
PROCNO                  1

F1 - Acquisition parameters
TD                    128
SFO1          100.6203 MHz
FIDRES      133.547012 Hz
SW             169.886 ppm
FnMODE               TPPI

F2 - Processing parameters
SI                   2048
SF         400.1300188 MHz
WDW                 QSINE
SSB                     2
LB          0 Hz
GB          0
PC                   1.40

F1 - Processing parameters
SI                    256
MC2                  TPPI
SF         100.6126855 MHz
WDW                 QSINE
SSB                     2
LB          0 Hz
GB          0
```

Facial exchange of complex 5.

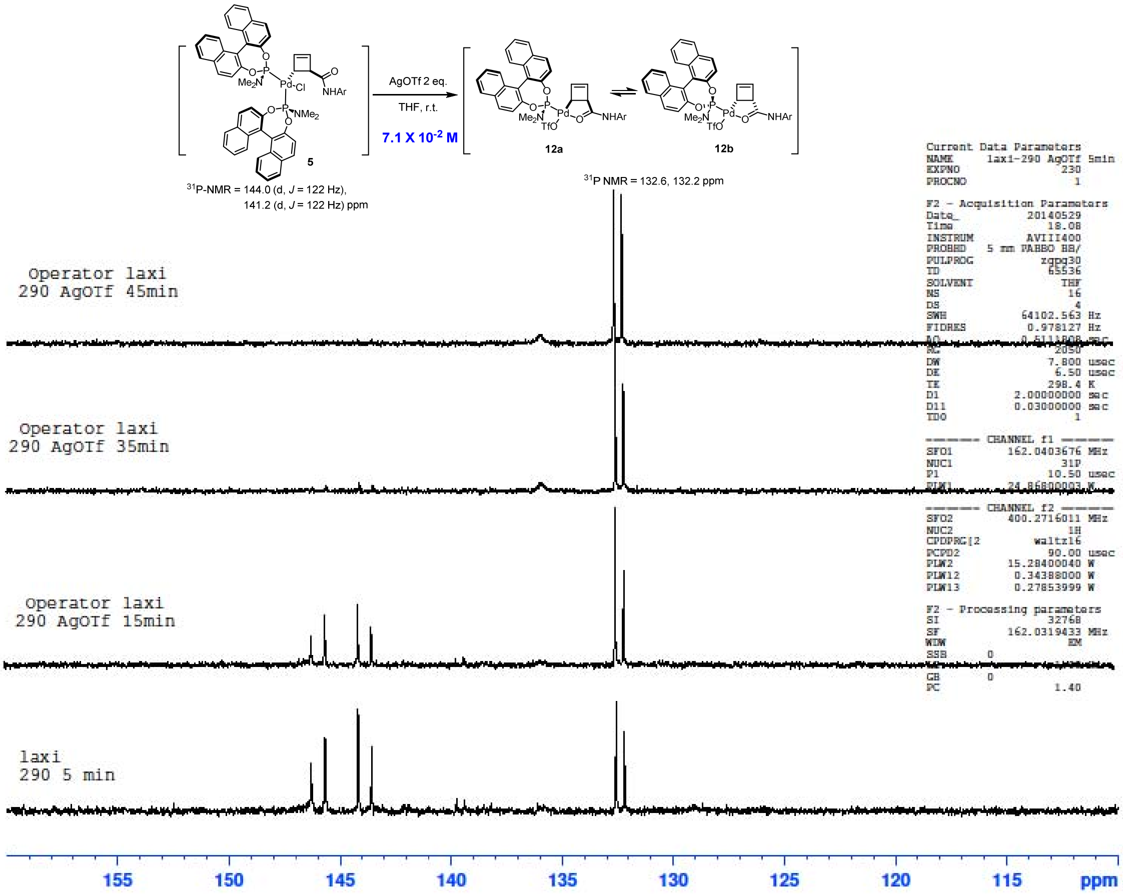

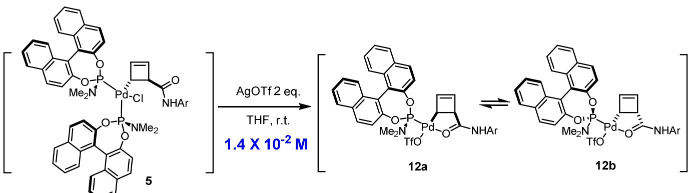

^{31}P-NMR = 144.0 (d, *J* = 122 Hz), 141.2 (d, *J* = 122 Hz) ppm

^{31}P NMR = 132.6, 132.2 ppm

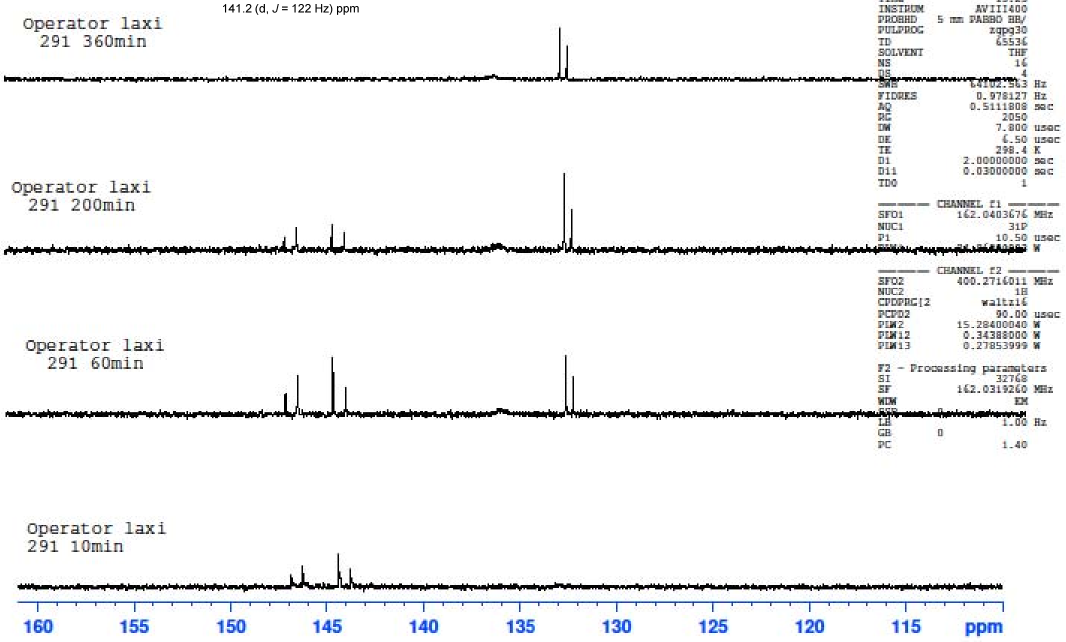

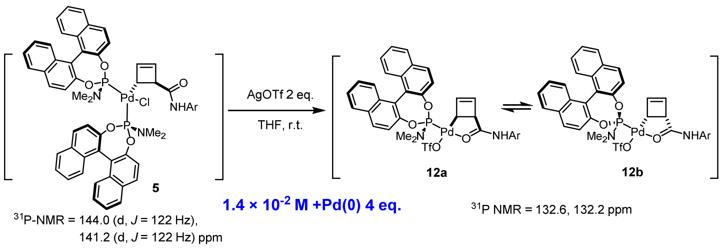
AgOTf 2 eq.
THF, r.t.
Me2N
Pd-Cl
NHAr
NMe2
5
TfO
12a
12b
1.4 × 10-2 M +Pd(0) 4 eq.
31P-NMR = 144.0 (d, J = 122 Hz), 141.2 (d, J = 122 Hz) ppm
31P NMR = 132.6, 132.2 ppm

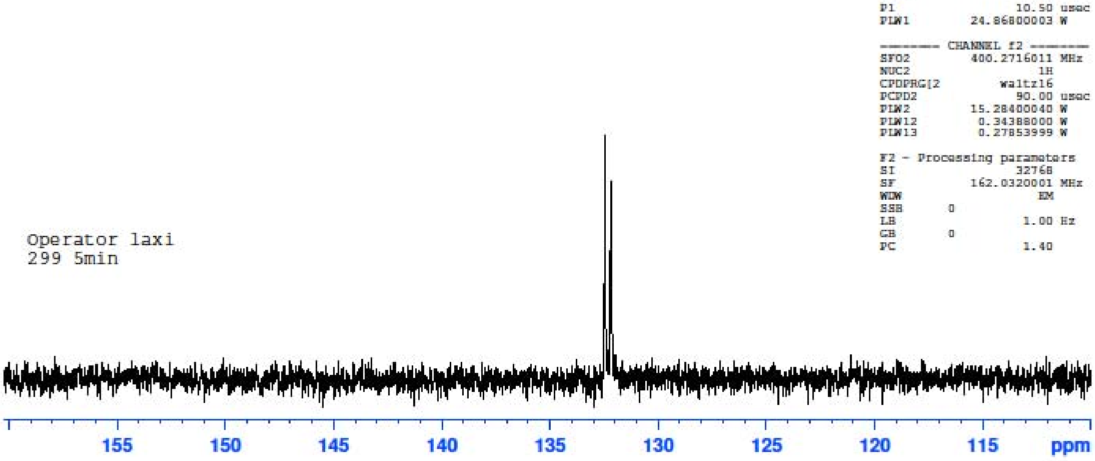
132.50
132.20
Current Data Parameters
NAME laxi-299
EXPNO 240
PROCNO 1
F2 - Acquisition Parameters
Date_ 20140605
Time 14.29
INSTRUM AVIII400
PROBHD 5 mm PABBO BB/
PULPROG zgpg30
TD 65536
SOLVENT THF
NS 16
DS 4
SWH 64102.563 Hz
FIDRES 0.978127 Hz
AQ 0.5111808 sec
RG 2050
DW 7.800 usec
DE 6.50 usec
TE 298.4 K
D1 2.00000000 sec
D11 0.03000000 sec
TD0 1
======== CHANNEL f1 ========
SFO1 162.0403676 MHz
NUC1 31P
P1 10.50 usec
PLW1 24.86800003 W
======== CHANNEL f2 ========
SFO2 400.2716011 MHz
NUC2 1H
CPDPRG[2 waltz16
PCPD2 90.00 usec
PLW2 15.28400040 W
PLW12 0.34388000 W
PLW13 0.27853999 W
F2 - Processing parameters
SI 32768
SF 162.0320001 MHz
WDW EM
SSB 0
LB 1.00 Hz
GB 0
PC 1.40
Operator laxi
299 5min
155
150
145
140
135
130
125
120
115
ppm

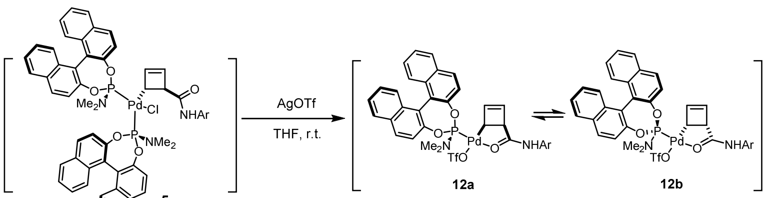

^{31}P-NMR = 144.0 (d, *J* = 122 Hz), 141.2 (d, *J* = 122 Hz) ppm

1.4 × 10^{-2} M + AgOTf 8eq.

^{31}P NMR = 132.6, 132.2 ppm

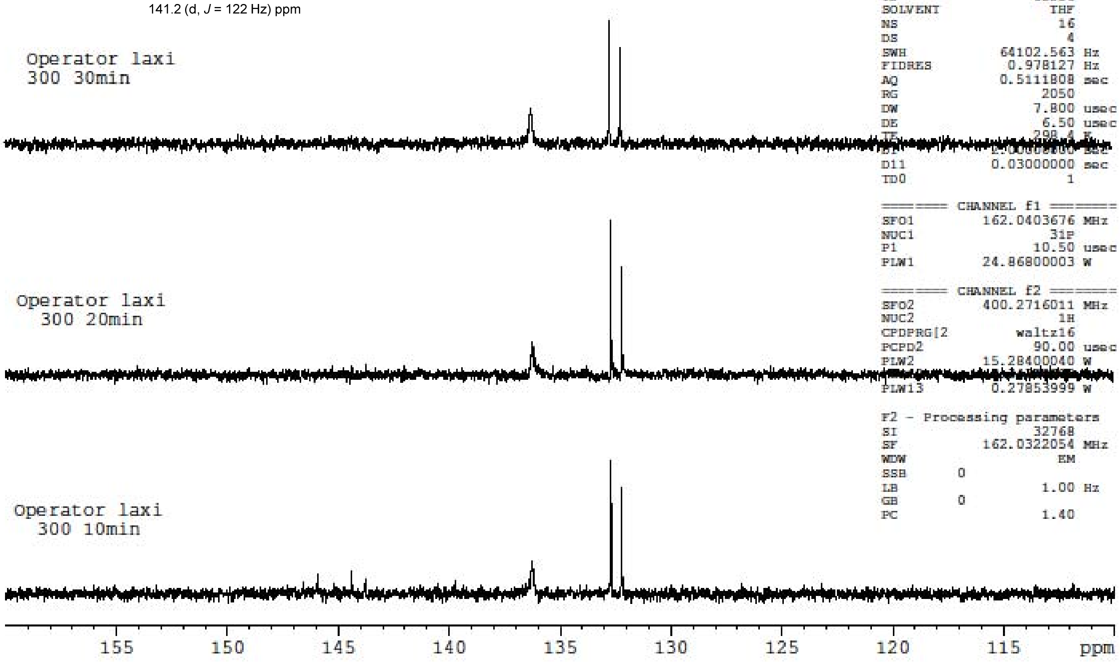

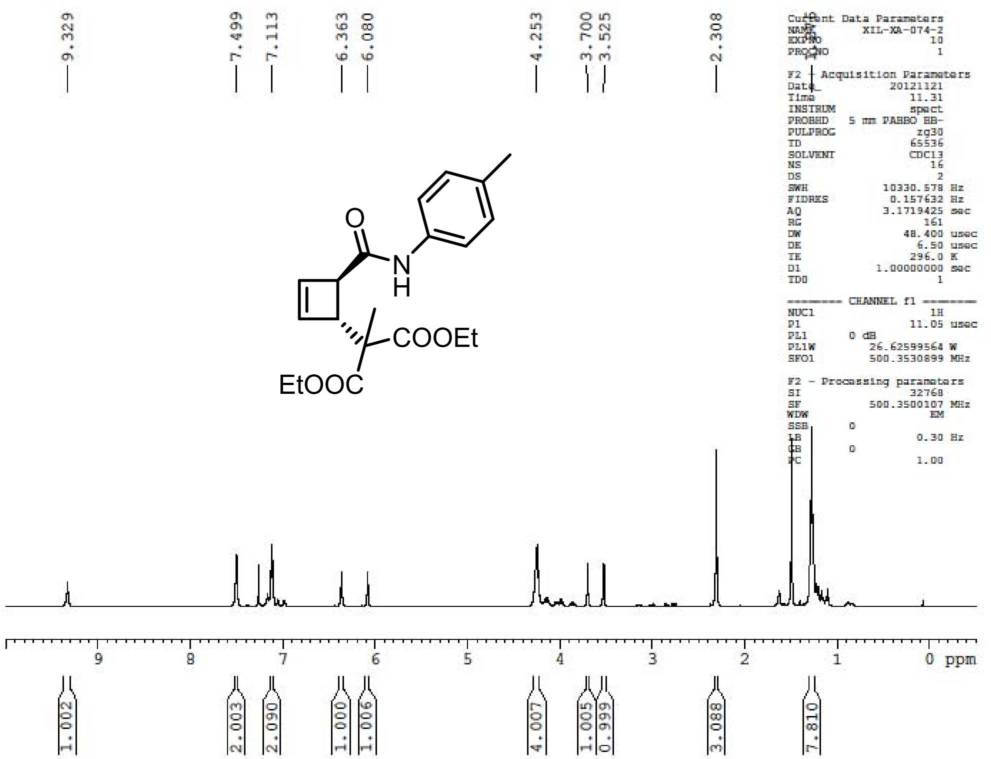

9.329
7.499
7.113
6.363
6.080
4.253
3.700
3.525
2.308
O
N
H
COOEt
EtOOC
Current Data Parameters
NAME XIL-XA-074-2
EXPNO 10
PROCNO 1
F2 - Acquisition Parameters
Date_ 20121121
Time 11.31
INSTRUM spect
PROBHD 5 mm PABBO BB-
PULPROG zg30
TD 65536
SOLVENT CDCl3
NS 16
DS 2
SWH 10330.578 Hz
FIDRES 0.157632 Hz
AQ 3.1719425 sec
RG 161
DW 48.400 usec
DE 6.50 usec
TE 296.0 K
D1 1.00000000 sec
TD0 1
======== CHANNEL f1 ========
NUC1 1H
P1 11.05 usec
PL1 0 dB
PL1W 26.62599564 W
SFO1 500.3530899 MHz
F2 - Processing parameters
SI 32768
SF 500.3500107 MHz
WDW EM
SSB 0
LB 0.30 Hz
GB 0
PC 1.00
9
8
7
6
5
4
3
2
1
0 ppm
1.002
2.003
2.090
1.000
1.006
4.007
1.005
0.999
3.088
7.810

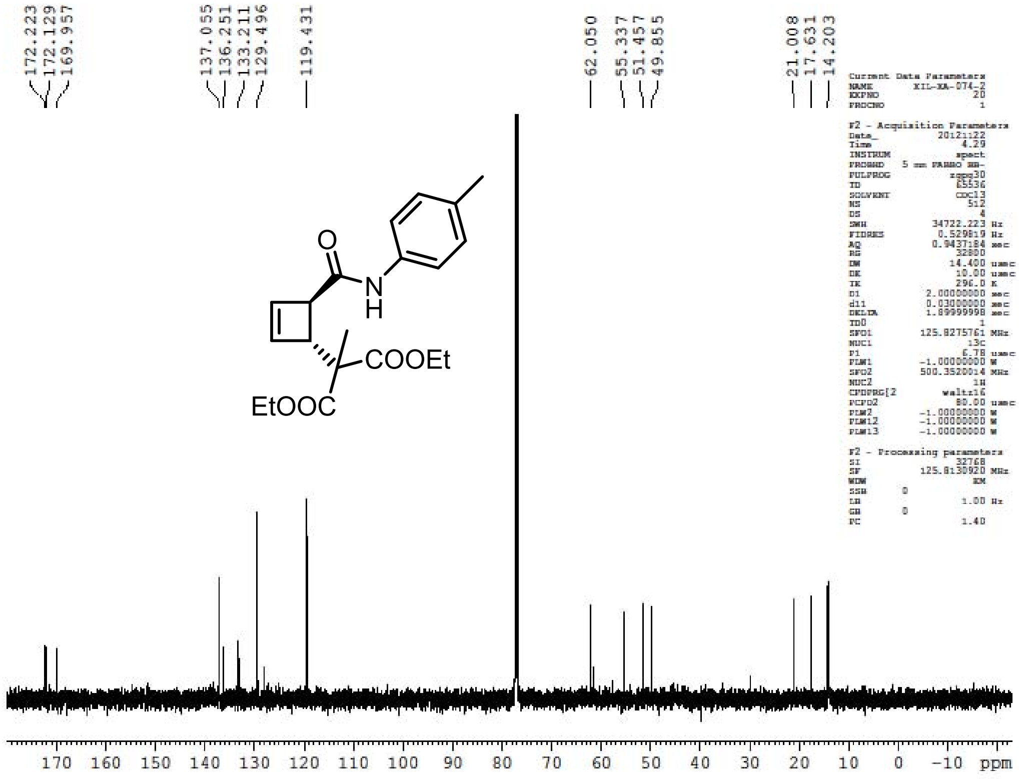

172.223
172.129
169.957
137.055
136.251
133.211
129.496
119.431
62.050
55.337
51.457
49.855
21.008
17.631
14.203
O
N
H
COOEt
EtOOC
Current Data Parameters
NAME XIL-XA-074-2
EXPNO 20
PROCNO 1
F2 - Acquisition Parameters
Date_ 20121122
Time 4.29
INSTRUM spect
PROBHD 5 mm PABBO BB-
PULPROG zgpg30
TD 65536
SOLVENT CDCl3
NS 512
DS 4
SWH 34722.223 Hz
FIDRES 0.529819 Hz
AQ 0.9437184 sec
RG 32800
DW 14.400 usec
DE 10.00 usec
TE 296.0 K
D1 2.00000000 sec
d11 0.03000000 sec
DELTA 1.89999998 sec
TD0 1
SFO1 125.8275761 MHz
NUC1 13C
P1 6.78 usec
PLW1 -1.00000000 W
SFO2 500.3520014 MHz
NUC2 1H
CPDPRG[2 waltz16
PCPD2 80.00 usec
PLW2 -1.00000000 W
PLW12 -1.00000000 W
PLW13 -1.00000000 W
F2 - Processing parameters
SI 32768
SF 125.8130920 MHz
WDW EM
SSB 0
LB 1.00 Hz
GB 0
PC 1.40
170
160
150
140
130
120
110
100
90
80
70
60
50
40
30
20
10
0
-10
ppm

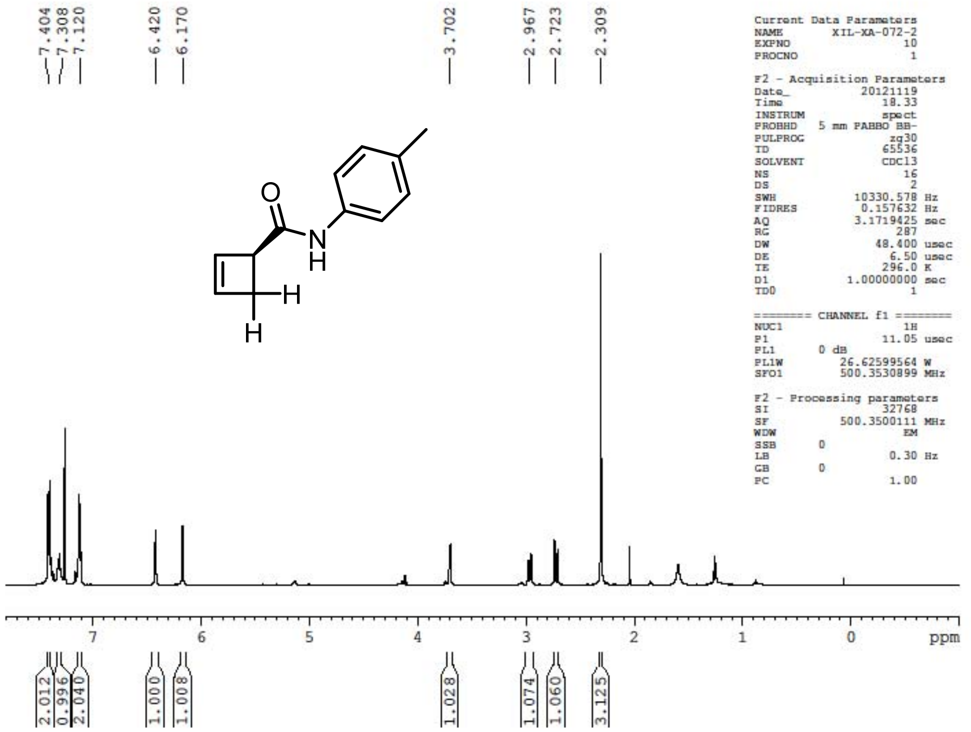
7.404
7.308
7.120
6.420
6.170
3.702
2.967
2.723
2.309
O
N
H
H
H
Current Data Parameters
NAME XIL-XA-072-2
EXPNO 10
PROCNO 1
F2 - Acquisition Parameters
Date_ 20121119
Time 18.33
INSTRUM spect
PROBHD 5 mm PABBO BB-
PULPROG zg30
TD 65536
SOLVENT CDCl3
NS 16
DS 2
SWH 10330.578 Hz
FIDRES 0.157632 Hz
AQ 3.1719425 sec
RG 287
DW 48.400 usec
DE 6.50 usec
TE 296.0 K
D1 1.00000000 sec
TD0 1
======== CHANNEL f1 ========
NUC1 1H
P1 11.05 usec
PL1 0 dB
PL1W 26.62599564 W
SFO1 500.3530899 MHz
F2 - Processing parameters
SI 32768
SF 500.3500111 MHz
WDW EM
SSB 0
LB 0.30 Hz
GB 0
PC 1.00
7
6
5
4
3
2
1
0
ppm
2.012
0.996
2.040
1.000
1.008
1.028
1.074
1.060
3.125

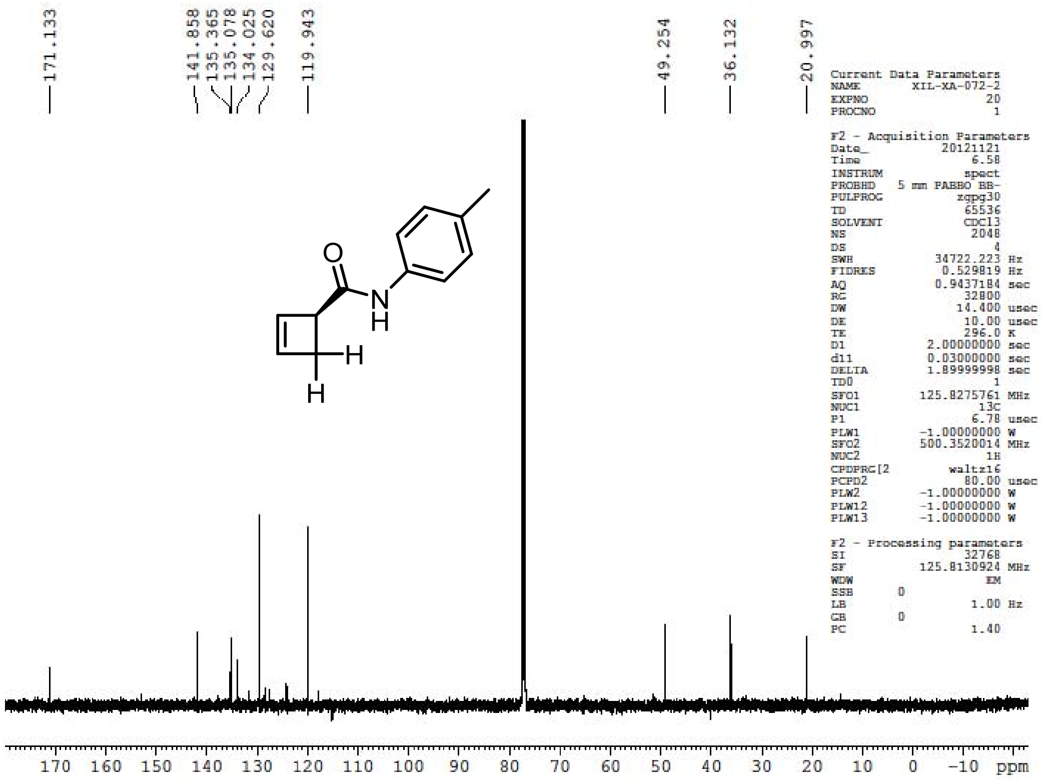
171.133
141.858
135.365
135.078
134.025
129.620
119.943
49.254
36.132
20.997
O
N
H
H
H
Current Data Parameters
NAME XIL-XA-072-2
EXPNO 20
PROCNO 1
F2 - Acquisition Parameters
Date_ 20121121
Time 6.58
INSTRUM spect
PROBHD 5 mm PABBO BB-
PULPROG zgpg30
TD 65536
SOLVENT CDCl3
NS 2048
DS 4
SWH 34722.223 Hz
FIDRES 0.529819 Hz
AQ 0.9437184 sec
RG 32800
DW 14.400 usec
DE 10.00 usec
TE 296.0 K
D1 2.00000000 sec
d11 0.03000000 sec
DELTA 1.89999998 sec
TD0 1
SFO1 125.8275761 MHz
NUC1 13C
P1 6.78 usec
PLW1 -1.00000000 W
SFO2 500.3520014 MHz
NUC2 1H
CPDPRG[2 waltz16
PCPD2 80.00 usec
PLW2 -1.00000000 W
PLW12 -1.00000000 W
PLW13 -1.00000000 W
F2 - Processing parameters
SI 32768
SF 125.8130924 MHz
WDW EM
SSB 0
LB 1.00 Hz
GB 0
PC 1.40
170 160 150 140 130 120 110 100 90 80 70 60 50 40 30 20 10 0 -10 ppm

Report

Sample Information

Sample Name	: Laxi-147
Vial#	: 16
Injection Volume	: 6
Method File	: equi_ISOHeptan8-IPA2.lcm
Report Format File	: ReportColumn1.lsr
Date Acquired	: 10.02.2014 16:46:47
Date Processed	: 10.02.2014 17:36:48
Batch File	: C:\LabSolutions\Data\BATCHES\NP\batch_10.02.2014_2.lcb
Data File	: C:\LabSolutions\Data\BATCHES\NP\Langui Xie\LaXi 147 10.02.2014.lcd

Method Description:
Column:Chiralpak AS-3
Solvent System: n-Heptan+0,1%IPA/IPA 8:2
Flow:0,7 ml/min
T=25°C P= 3,7 MPa

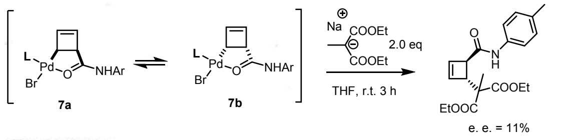

Chromatogram

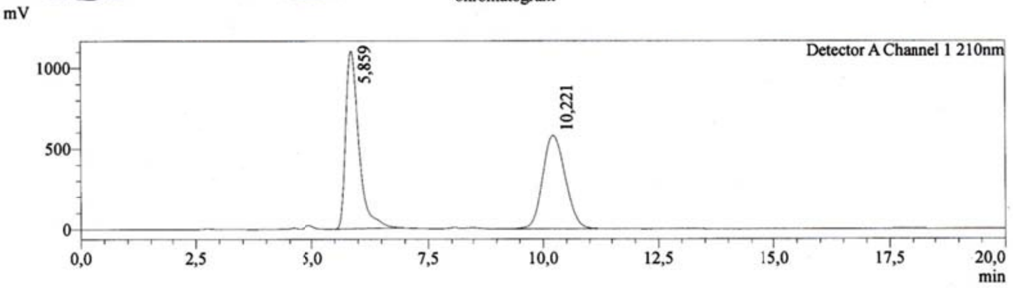

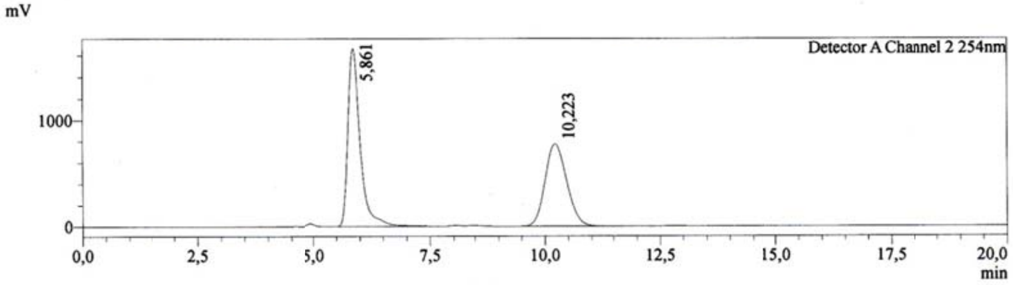

Peak Table

Detector A Channel 1 210nm

Peak#	Ret. Time	Area	Area%
1	5,859	21741537	52,788
2	10,221	19444905	47,212
Total		41186442	100,000

Detector A Channel 2 254nm

Peak#	Ret. Time	Area	Area%
1	5,861	30841864	55,309
2	10,223	24920679	44,691
Total		55762543	100,000

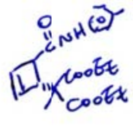

Supporting Information
Computational Part

Dynamic behavior of monohaptoallylpalladium species: internal coordination as a driving force in allylic alkylation chemistry

Lan-Gui Xie, Viktor Bagutski, Davide Audisio, Larry Wolf, Volker Schmidts, Kathrin Hofmann, Cornelia Wirtz, Walter Thiel, Christina M. Thiele* and Nuno Maulide*

nuno.maulide@univie.ac.at

cthiele@thielelab.de

Table of Contents

Computational Methods S2

Dissociative Mechanism S3

Tables and Figures S5

Cartesians Coordinates of Optimized Geometries S12

References S34

Computational Methods

Density functional theory (DFT) was applied to study the mechanism for racemization of **7a** and **7b** (Scheme 3) as well as for the exclusive formation of the single diastereomer **5** (Scheme 2). All geometry optimizations were performed using the B3LYP[1] and TPSS[2] functionals with and without inclusion of Grimme's DFT-D3[3] empirical dispersion correction (B3LYP-D3 and TPSS-D3). For geometry optimizations, the def2-TZVP[4] basis set was used for Cl and Pd and the def2-SVP basis set was used for all remaining atoms. The 28 inner-shell core electrons of the palladium atom were described by an effective core potential[5] accounting for scalar relativistic effects (def2-ecp). For the purpose of computational efficiency, the resolution-of-identity (RI) approximation[6] was applied using auxiliary basis sets to approximate Coulomb potentials in conjunction with the multipole accelerated resolution of the identity approximation (MA-RI) method.[7]

Stationary points were characterized by evaluating the harmonic vibrational frequencies at the optimized geometries. Zero-point vibrational energies (ZPVE) were computed from the corresponding harmonic vibrational frequencies without scaling. Relative Gibbs free energies (ΔG) were determined at standard pressure (1 bar) and at room temperature (298.15 K). The thermal and entropic contributions were evaluated within the rigid-rotor harmonic-oscillator approximation. Single-point energies were computed at the B3LYP-D3, TPSS-D3, M06L[8]-D3, and M06[9]-D3 level using the def2-TZVP basis set for all atoms. Solvation contributions were examined for THF at the optimized gas-phase geometries employing the SMD solvation model.[10]

All geometry optimizations were performed with TURBOMOLE (version 6.4)[11] and single-point SMD solvation calculations. M06 and M06L single-point calculations were carried out with Gaussian09 using ultrafine grids.[12]

Dissociative Mechanism

A mechanism involving initial phosphine dissociation was also considered since the stabilizing interaction between palladium and the proximal amide carbonyl in the square planar complexes **7a** and **7b** could be suspected to persist during the formation of the η^3 intermediates (**7a-INT1** and **7b-INT1**), thus promoting dissociation of the phosphine ligand from a possible 18 electron complex. The overall barrier height is computed to be prohibitively high ($\Delta G^{\ddagger}_{\text{diss}}$ = 32.4 kcal/mol) as compared to the associative mechanism ($\Delta G^{\ddagger}$ = 19.7 kcal/mol). On account of geometrical constraints within a cyclobutyl allyl moiety, there is effectively very little bonding interaction between the carbonyl and the palladium atom, which is also reflected in the result that the η^3 geometry is slightly more favorable when the carbonyl group is directed away from the palladium (**7-INT3-diss**) rather than toward the palladium (**7-INT2-diss**). The dissociative mechanism is thus highly disfavored because of the lack of bonding interactions between the carbonyl and the palladium atom in the $\eta^1 \rightarrow \eta^3$ transition state resulting in a high-energy 14 electron intermediate (**7-INT2-diss**).

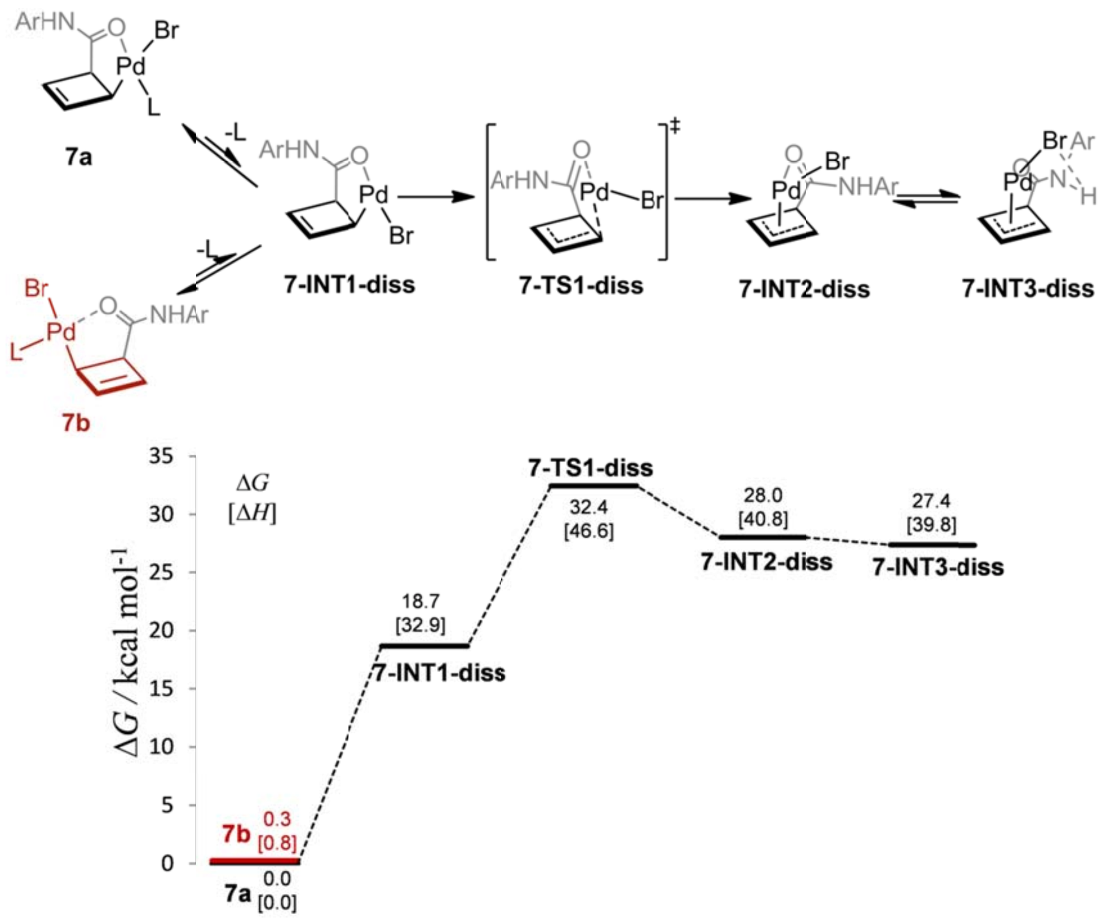

Figure S1. Computed Gibbs free energy profile (298.15 K) for phosphine dissociation and subsequent $\eta^1 \rightarrow \eta^3$ transition. SMD(THF)-B3LYP-D3/def2-TZVP//B3LYP-D3/def2-SVP(def2-TZVP for Pd). L=L2c.

Tables and Figures

Stationary points for the energy profile from Figure 3 were located with and without the D3 dispersion correction (Table S1). With the D3 dispersion correction applied, barrier heights tend to be greater, η-1 intermediates are higher in energy (~1 kcal/mol), and η-3 intermediates are lower in energy (~2-3 kcal/mol) relative to the results without the D3 dispersion correction. The dispersion-corrected energies are in better agreement with the overall experimental barrier height ($\Delta G^{\ddagger}_{calc}$ = 17.9 kcal/mol; $\Delta G^{\ddagger}_{obs}$ = 21.1 kcal/mol) and equilibrium constant (ΔG_{calc} = -0.3 kcal/mol; ΔG_{obs} = -0.1 kcal/mol).

Table S1. Relative electronic energies Δ***E***, electronic energies with ZPVE, enthalpies Δ***H***, and free energies Δ***G*** (kcal/mol) with respect to complex **7a** obtained at the indicated level of theory.

Method	7b	7a-INT2	7a-TS2	7a-INT1	7a-TS1	7a	7b-INT2	7b-TS2	7b-INT1	7b-TS1
					Δ***E***					
B3LYP/def2-SVP[a]	0.24	4.78	16.12	9.94	12.58	0.00	6.91	17.81	11.65	14.21
B3LYP/def2-TZVP	0.12	5.97	17.15	9.13	12.76	0.00	8.04	18.81	12.12	14.60
SMD-B3LYP/def2-TZVP	-0.18	7.00	17.11	8.48	11.79	0.00	8.56	18.69	10.53	13.45
B3LYP-D3/def2-SVP[a]	1.79	0.48	19.00	5.14	14.25	0.00	8.36	18.36	5.49	11.11
B3LYP-D3/def2-TZVP	1.40	3.12	19.85	6.29	14.31	0.00	9.26	19.61	5.05	11.51
SMD-B3LYP-D3/def2-TZVP	0.79	6.12	19.27	6.02	13.17	0.00	9.46	19.64	5.78	10.85
					Δ***E*** + ΔZPVE					
B3LYP/def2-SVP[a]	0.21	4.35	15.35	9.26	12.01	0.00	6.54	17.01	11.04	13.57
B3LYP/def2-TZVP	0.09	5.54	16.38	8.45	12.18	0.00	7.68	18.01	11.51	13.97
SMD-B3LYP/def2-TZVP	-0.21	6.58	16.34	7.80	11.21	0.00	8.20	17.89	9.92	12.82
B3LYP-D3/def2-SVP[a]	1.74	0.29	18.21	4.75	13.51	0.00	8.10	17.66	5.22	10.72
B3LYP-D3/def2-TZVP	1.35	2.93	19.06	5.91	13.56	0.00	9.00	18.91	4.78	11.12
SMD-B3LYP-D3/def2-TZVP	0.74	5.93	18.48	5.64	12.42	0.00	9.20	18.94	5.51	10.46
					Δ***H***					
B3LYP/def2-SVP[a]	0.24	4.48	15.16	9.53	11.77	0.00	6.64	16.83	11.27	13.34
B3LYP/def2-TZVP	0.12	5.67	16.19	8.73	11.94	0.00	7.78	17.83	11.74	13.73
SMD-B3LYP/def2-TZVP	0.01	3.69	15.46	7.78	12.01	0.00	6.36	16.73	9.92	13.87
B3LYP-D3/def2-SVP[a]	1.79	0.24	18.03	4.88	13.37	0.00	8.14	17.38	5.34	9.83
B3LYP-D3/def2-TZVP	1.40	2.88	18.88	6.04	13.43	0.00	9.04	18.62	4.90	10.23
SMD-B3LYP-D3/def2-TZVP	0.79	5.89	18.30	5.76	12.29	0.00	9.24	18.65	5.63	9.57
					Δ***G***					
B3LYP/def2-SVP[a]	0.01	3.69	15.46	7.78	12.01	0.00	6.36	16.73	9.92	13.87
B3LYP/def2-TZVP	-0.12	4.88	16.49	6.97	12.19	0.00	7.50	17.73	10.39	14.27
SMD-B3LYP/def2-TZVP	-0.42	5.91	16.46	6.32	11.22	0.00	8.02	17.61	8.80	13.12
B3LYP-D3/def2-SVP[a]	1.27	1.63	17.68	4.30	12.96	0.00	7.17	18.41	4.41	13.07
B3LYP-D3/def2-TZVP	0.87	4.27	18.53	5.45	13.02	0.00	8.07	19.66	3.97	13.47
SMD-B3LYP-D3/def2-TZVP	0.27	7.27	17.95	5.18	11.88	0.00	8.27	19.69	4.70	12.81

[a] The def2-TZVP basis set was used for Pd (def2-ecp), and the def2-SVP basis set was used for all remaining atoms during geometry optimization.

Complexes **5a** and **5b** (Figure 4 of main text) exhibit interactions that are notoriously difficult to describe with standard DFT. The next low energy conformers of each diasteromer are provided for comparison (see **5a′** and **5b′**, Figure S2). It is worth noting that optimization without the D3 correction (TPSS) results in structures that lack π-stacking interactions. Inclusion of dispersion corrections is important for describing this system, as otherwise the diastereomer inconsistent with experiment would be predicted (Table S2).

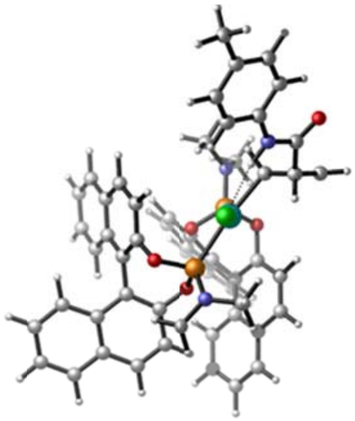

5a

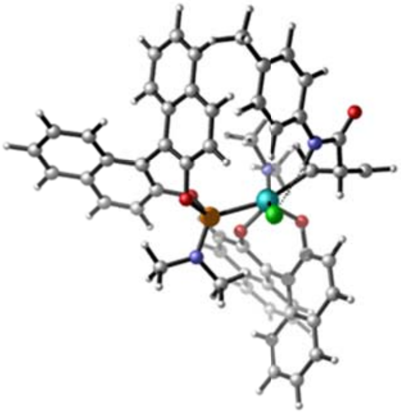

5a′

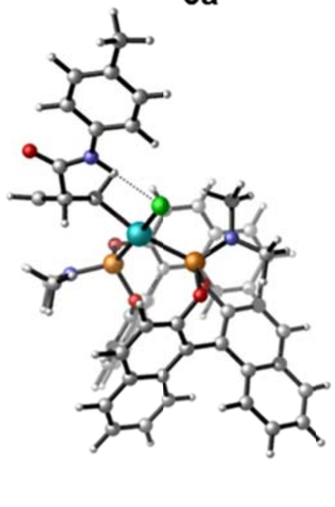

5b

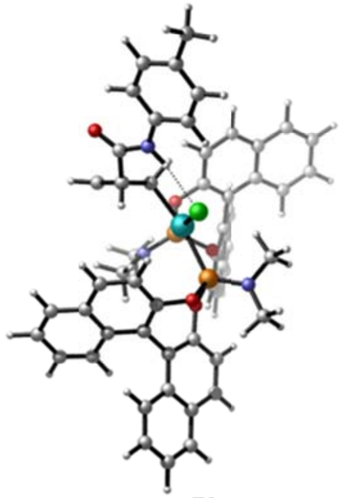

5b′

Figure S2. Complexes **5a** and **5a′** correspond to conformers of **5** (Scheme 2 of main text) while **5b** and **5b′** correspond to conformers of its diastereomer.

Table S2. Relative electronic energies Δ***E***, electronic energies with ZPVE, enthalpies Δ***H***, and free energies Δ***G*** (kcal/mol) with respect to complex **5a′** excluding the D3 correction and with respect to **5a** including the D3 correction obtained at the indicated level of theory.

	**5**	**5a**	**5b**	**5`**	**5a`**	**5b`**
**Method**			Δ***E***			
TPSS/def2-SVP[a]	-	-	0.00	-	-	-1.57
TPSS/def2-TZVP	-	-	0.00	-	-	-1.64
TPSS-D3/def2-SVP	0.00	1.22	-	0.90	0.95	-
TPSS-D3/def2-TZVP	0.00	0.20	-	1.18	0.23	-
M06L-D3/def2-TZVP	0.00	2.32	-	1.21	1.58	-
B3LYP-D3/def2-TZVP	0.00	1.04	-	1.30	1.23	-
M06/def2-TZVP	0.00	1.66	-	1.23	0.40	-
M06-D3/def2-TZVP	0.00	2.84	-	1.25	3.34	-
SMD[b]-M06-D3/def2-TZVP	0.00	2.37	-	1.52	2.30	-
			Δ***E*** + Δ***ZPVE***			
TPSS/def2-SVP	-	-	0.00	-	-	-1.76
TPSS/def2-TZVP	-	-	0.00	-	-	-1.82
TPSS-D3/def2-SVP	0.00	0.67	-	0.93	0.74	-
TPSS-D3/def2-TZVP	0.00	-0.35	-	1.21	0.01	-
M06L-D3/def2-TZVP	0.00	1.77	-	1.24	1.36	-
B3LYP-D3/def2-TZVP	0.00	0.49	-	1.34	1.01	-
M06/def2-TZVP	0.00	1.11	-	1.26	0.18	-
M06-D3/def2-TZVP	0.00	2.29	-	1.28	3.13	-
SMD-M06-D3/def2-TZVP	0.00	1.82	-	1.55	2.08	-
			Δ***H***			
TPSS/def2-SVP	-	-	0.00	-	-	-1.62
TPSS/def2-TZVP	-	-	0.00	-	-	-1.69
TPSS-D3/def2-SVP	0.00	1.07	-	1.42	1.00	-
TPSS-D3/def2-TZVP	0.00	0.05	-	1.71	0.27	-
M06L-D3/def2-TZVP	0.00	2.17	-	1.74	1.62	-
B3LYP-D3/def2-TZVP	0.00	0.88	-	1.83	1.27	-
M06/def2-TZVP	0.00	1.51	-	1.75	0.44	-
M06-D3/def2-TZVP	0.00	2.69	-	1.78	3.39	-
SMD-M06-D3/def2-TZVP	0.00	2.20	-	2.05	2.35	-
			Δ***G***			
TPSS/def2-SVP	-	-	0.00	-	-	-1.27
TPSS/def2-TZVP	-	-	0.00	-	-	-1.34
TPSS-D3/def2-SVP	0.00	-1.11	-	-1.21	-0.68	-
TPSS-D3/def2-TZVP	0.00	-2.13	-	-0.92	-1.40	-
M06L-D3/def2-TZVP	0.00	0.00	-	-0.89	-0.06	-
B3LYP-D3/def2-TZVP	0.00	-1.29	-	-0.80	-0.41	-
M06/def2-TZVP	0.00	-0.67	-	-0.88	-1.24	-
M06-D3/def2-TZVP	0.00	0.67	-	-1.38	1.67	-

SMD-M06-D3/def2-TZVP	0.00	0.05	-	-0.58	0.67	-

[a] The def2-TZVP basis set was used for Pd (with def2-ecp) and Cl, and the def2-SVP basis set was used for all remaining atoms during geometry optimization. [b] THF was used for the SMD solvation model.

Table S3. Electronic energies for Table S1 obtained at the indicated level of theory.

Method	7b
B3LYP/def2-SVP	-4689.613882
B3LYP/def2-TZVP	-4691.843501
SMD-B3LYP/def2-TZVP	-4693.157617
B3LYP-D3/def2-SVP	-4689.709901
B3LYP-D3/def2-TZVP	-4691.939683
SMD-B3LYP-D3/def2-TZVP	-4693.253158
	**7a-INT2**
B3LYP/def2-SVP	-4689.606658
B3LYP/def2-TZVP	-4691.83418
SMD-B3LYP/def2-TZVP	-4693.146164
B3LYP-D3/def2-SVP	-4689.711999
B3LYP-D3/def2-TZVP	-4691.936944
SMD-B3LYP-D3/def2-TZVP	-4693.24467
	**7a-TS2**
B3LYP/def2-SVP	-4689.588579
B3LYP/def2-TZVP	-4691.816361
SMD-B3LYP/def2-TZVP	-4693.130051
B3LYP-D3/def2-SVP	-4689.682473
B3LYP-D3/def2-TZVP	-4691.910273
SMD-B3LYP-D3/def2-TZVP	-4693.22371
	**7a-INT1**
B3LYP/def2-SVP	-4689.598433
B3LYP/def2-TZVP	-4691.829146
SMD-B3LYP/def2-TZVP	-4693.143808
B3LYP-D3/def2-SVP	-4689.704573
B3LYP-D3/def2-TZVP	-4691.931887
SMD-B3LYP-D3/def2-TZVP	-4693.244827
	**7a-TS1**
B3LYP/def2-SVP	-4689.594221
B3LYP/def2-TZVP	-4691.82336
SMD-B3LYP/def2-TZVP	-4693.138537
B3LYP-D3/def2-SVP	-4689.690051
B3LYP-D3/def2-TZVP	-4691.919116

SMD-B3LYP-D3/def2-TZVP	-4693.233442
	**7a**
B3LYP/def2-SVP	-4689.614271
B3LYP/def2-TZVP	-4691.843694
SMD-B3LYP/def2-TZVP	-4693.157325
B3LYP-D3/def2-SVP	-4689.712758
B3LYP-D3/def2-TZVP	-4691.941913
SMD-B3LYP-D3/def2-TZVP	-4693.254422
	**7b-INT2**
B3LYP/def2-SVP	-4689.603267
B3LYP/def2-TZVP	-4691.83088
SMD-B3LYP/def2-TZVP	-4693.14368
B3LYP-D3/def2-SVP	-4689.699439
B3LYP-D3/def2-TZVP	-4691.927163
SMD-B3LYP-D3/def2-TZVP	-4693.239352
	**7b-TS2**
B3LYP/def2-SVP	-4689.585892
B3LYP/def2-TZVP	-4691.813724
SMD-B3LYP/def2-TZVP	-4693.127541
B3LYP-D3/def2-SVP	-4689.683495
B3LYP-D3/def2-TZVP	-4691.910663
SMD-B3LYP-D3/def2-TZVP	-4693.223127
	**7b-INT1**
B3LYP/def2-SVP	-4689.595704
B3LYP/def2-TZVP	-4691.824384
SMD-B3LYP/def2-TZVP	-4693.140545
B3LYP-D3/def2-SVP	-4689.704011
B3LYP-D3/def2-TZVP	-4691.933861
SMD-B3LYP-D3/def2-TZVP	-4693.245207
	**7b-TS1**
B3LYP/def2-SVP	-4689.591634
B3LYP/def2-TZVP	-4691.820422
SMD-B3LYP/def2-TZVP	-4693.135888
B3LYP-D3/def2-SVP	-4689.695049
B3LYP-D3/def2-TZVP	-4691.923578
SMD-B3LYP-D3/def2-TZVP	-4693.237139

Table S4. Electronic energies for Table S2 obtained at the indicated level of theory.

**Method**	**5**
TPSS-D3/def2-SVP	-3972.837878
TPSS-D3/def2-TZVP	-3975.939608
M06L-D3/def2-TZVP	-3975.024429
B3LYP-D3/def2-TZVP	-3975.547577
M06/def2-TZVP	-3973.307143
M06-D3/def2-TZVP	-3973.351341
SMD-M06-D3/def2-TZVP	-3973.407805
	**5a**
TPSS-D3/def2-SVP	-3972.835936
TPSS-D3/def2-TZVP	-3975.939286
M06L-D3/def2-TZVP	-3975.020725
B3LYP-D3/def2-TZVP	-3975.545923
M06/def2-TZVP	-3973.304499
M06-D3/def2-TZVP	-3973.34681
SMD-M06-D3/def2-TZVP	-3973.404021
	**5b**
TPSS/def2-SVP	-3972.682871
TPSS/def2-TZVP	-3975.786014
	**5`**
TPSS-D3/def2-SVP	-3972.836452
TPSS-D3/def2-TZVP	-3975.937726
M06L-D3/def2-TZVP	-3975.022501
B3LYP-D3/def2-TZVP	-3975.5455
M06/def2-TZVP	-3973.305189
M06-D3/def2-TZVP	-3973.349347
SMD-M06-D3/def2-TZVP	-3973.40538
	**5a`**
TPSS-D3/def2-SVP	-3972.836361
TPSS-D3/def2-TZVP	-3975.93924
M06L-D3/def2-TZVP	-3975.021917
B3LYP-D3/def2-TZVP	-3975.545623
M06/def2-TZVP	-3973.306509
M06-D3/def2-TZVP	-3973.34601
SMD-M06-D3/def2-TZVP	-3973.404137
	**5b`**
TPSS-D3/def2-SVP	-3972.685375
TPSS-D3/def2-TZVP	-3975.788624

Coordinates

(**S-** indicates optimized without D3 correction)

S-7a

C	6.1008653	0.2507430	2.1246090
H	6.8044019	-0.5558047	2.3837912
C	4.7802487	0.2492584	2.8280300
H	4.5222999	0.3402987	3.8859086
C	4.0457862	0.0890892	1.7073127
H	2.9754076	0.0237211	1.4924234
C	5.2721382	0.0302745	0.7954058
H	5.4324378	-0.9387274	0.2863833
C	5.4253845	1.1773779	-0.1753622
C	4.7385721	1.9944774	-2.4486053
C	4.0151046	1.6017575	-3.5851816
H	3.5360449	0.6176762	-3.6136039
C	3.8988491	2.4558467	-4.6822242
H	3.3284528	2.1251673	-5.5549658
C	4.4980042	3.7240559	-4.6827923
C	5.2187044	4.0991815	-3.5370098
H	5.6993610	5.0815286	-3.5024064
C	5.3478184	3.2615635	-2.4285433
H	5.9134736	3.5830106	-1.5565155
C	4.3797512	4.6563363	-5.8639797
H	3.7727735	4.2159992	-6.6692317
H	5.3708088	4.8990104	-6.2838176
H	3.9127735	5.6129073	-5.5741681
C	10.4189147	1.5605439	2.9137145
H	11.0893504	0.6924199	3.0462549
H	11.0271521	2.4490189	2.6708559
H	9.7553561	1.3680320	2.0593205
C	10.4247606	2.0800727	5.3185050
H	9.7756871	2.2783619	6.1786055
H	11.0526562	2.9736723	5.1506695
H	11.0867071	1.2301845	5.5618084
C	6.4398042	3.2681767	5.6620906
C	5.6980552	4.3552018	5.1425565
H	6.1676530	4.9811729	4.3807093
C	4.4199772	4.5740929	5.5996032
H	3.8365240	5.4159109	5.2169020
C	3.8216870	3.6935339	6.5447095
C	2.4753393	3.8752924	6.9702069
H	1.9189399	4.7335334	6.5825849
C	1.8750169	2.9868482	7.8364863
H	0.8387045	3.1367586	8.1505247
C	2.6015062	1.8653785	8.3058777
H	2.1171274	1.1451640	8.9707485
C	3.9143540	1.6693481	7.9274712
H	4.4533962	0.7946461	8.2926126
C	4.5776292	2.5813607	7.0553469
C	5.9461897	2.4068151	6.6356543
C	6.8296834	1.3255258	7.1621543
C	7.4586506	0.4593270	6.2698115
C	8.2532362	-0.6300153	6.7055146
H	8.6850096	-1.2870575	5.9480385
C	8.4559846	-0.8388330	8.0486225
H	9.0589758	-1.6841113	8.3914498
C	7.9138219	0.0576431	9.0112561
C	8.1787367	-0.1049786	10.4005011
H	8.7832672	-0.9590432	10.7191408
C	7.7018511	0.7978846	11.3262509
H	7.9175950	0.6649858	12.3895784
C	6.9439364	1.9128530	10.8917902
H	6.5883804	2.6441104	11.6225534
C	6.6551456	2.0901540	9.5538639
H	6.0800027	2.9611390	9.2394935
C	7.1085698	1.1642760	8.5688430
Br	8.0736214	4.4061479	1.8092680
N	4.8085019	1.0722101	-1.3734178
H	4.3056450	0.2044423	-1.5250865
N	9.6369687	1.7861488	4.1247064
O	6.0823865	2.1843907	0.1473852
O	7.7232882	3.0814940	5.1781835
O	7.3022383	0.6148959	4.9083463
P	7.9804249	1.8957301	4.0493071
Pd	7.0404142	2.0901015	2.0551552

7a

C	6.0837065	0.3766019	2.3836745
H	6.6854565	-0.5166096	2.6125476
C	4.8769756	0.6266783	3.2348356
H	4.7704365	0.8167150	4.3051754
C	3.9950071	0.5453667	2.2161321
H	2.9111733	0.6595712	2.1267305
C	5.0736720	0.2422034	1.1735711
H	5.0145860	-0.7588855	0.7075435
C	5.2807530	1.3136237	0.1290585
C	4.4915795	2.1188160	-2.1105495
C	3.6389918	1.7682913	-3.1690180
H	3.0370530	0.8559213	-3.1061542
C	3.5507884	2.5731855	-4.3052707
H	2.8791638	2.2771959	-5.1161253
C	4.3069970	3.7484338	-4.4226621
C	5.1530862	4.0843241	-3.3532252
H	5.7559240	4.9954179	-3.4121998
C	5.2568647	3.2951855	-2.2074038
H	5.9228655	3.5834453	-1.3970254
C	4.2268427	4.6264274	-5.6474972
H	3.5001846	4.2416903	-6.3789531
H	5.2062695	4.6979176	-6.1505358

H	3.9272092	5.6543050	-5.3817889
C	10.7666763	1.6506057	2.9225408
H	11.4385741	0.7754249	2.9809017
H	11.3718404	2.5651865	2.8022678
H	10.1342473	1.5587180	2.0297861
C	10.6776223	1.8897780	5.3782566
H	9.9889095	2.0109844	6.2231228
H	11.3225412	2.7856961	5.3345637
H	11.3148188	1.0073416	5.5660622
C	6.6430144	3.1656593	5.4810504
C	5.9150630	4.1721687	4.8046192
H	6.4460806	4.7838317	4.0719353
C	4.5726294	4.3149807	5.0665547
H	3.9929806	5.0881799	4.5554876
C	3.9073912	3.4371593	5.9693896
C	2.5048344	3.5342876	6.1924704
H	1.9507877	4.3292251	5.6851462
C	1.8501350	2.6416220	7.0144107
H	0.7716550	2.7256657	7.1717095
C	2.5771074	1.6010031	7.6436925
H	2.0531497	0.8786199	8.2751638
C	3.9406187	1.4875868	7.4626259
H	4.4837835	0.6760547	7.9477270
C	4.6546418	2.4072650	6.6407145
C	6.0726714	2.3085328	6.4135289
C	6.9361876	1.2865035	7.0705890
C	7.6943823	0.4236673	6.2820501
C	8.4970535	-0.6005280	6.8411178
H	9.0428509	-1.2534771	6.1573547
C	8.5674374	-0.7503671	8.2055248
H	9.1766560	-1.5462845	8.6426048
C	7.8734816	0.1419953	9.0698008
C	7.9861402	0.0372918	10.4848557
H	8.5983753	-0.7677303	10.9014610
C	7.3522288	0.9351335	11.3169136
H	7.4508945	0.8467311	12.4018963
C	6.5836417	1.9871246	10.7599725
H	6.1005814	2.7131133	11.4192905
C	6.4440282	2.1091428	9.3926056
H	5.8568403	2.9305991	8.9815448
C	7.0636486	1.1851307	8.5015962
Br	8.4878674	4.2547675	1.7126789
N	4.5291034	1.2492192	-0.9913490
H	3.9002442	0.4551090	-1.0444057
N	9.9458384	1.7353689	4.1251621
O	6.0988698	2.2315493	0.3260191
O	7.9802639	3.0072233	5.1551262
O	7.6702457	0.5159681	4.9077017
P	8.2960185	1.8150109	4.0402608
Pd	7.2580467	2.0560485	2.1170258

S-7a-TS1
(-86.53 cm^{-1})

C	-1.1348888	-1.7248914	-1.2563566
H	-0.4585076	-2.3362299	-0.6511077
C	-2.5610124	-1.4775558	-1.0322896
H	-3.1660995	-1.3710130	-0.1296898
C	-2.8444478	-1.4029930	-2.3737716
H	-3.7342210	-1.1558039	-2.9561482
C	-1.4767750	-1.9753842	-2.7751983
H	-1.5483461	-3.0531886	-3.0102631
C	-0.6641759	-1.2268217	-3.8182986
C	0.4438371	-1.5528691	-6.0511519
C	0.7611442	-2.5376698	-7.0006254
H	0.4512729	-3.5745766	-6.8340568
C	1.4682290	-2.2083187	-8.1572186
H	1.7008592	-2.9956788	-8.8801027
C	1.8834678	-0.8920710	-8.4067147
C	1.5586614	0.0801160	-7.4462742
H	1.8651725	1.1180283	-7.6094349
C	0.8516533	-0.2270553	-6.2832931
H	0.6107988	0.5453449	-5.5562697
C	2.6431088	-0.5244060	-9.6582687
H	2.8561806	-1.4087700	-10.2775671
H	3.6056619	-0.0421501	-9.4179019
H	2.0734877	0.1890199	-10.2782847
C	2.3247629	1.4108035	-1.3802737
H	3.2898025	0.9127157	-1.5858661
H	2.4235383	2.4830211	-1.6240271
H	1.5608318	0.9835547	-2.0452692
C	2.8907127	1.7896229	0.9873645
H	2.5132945	1.6762653	2.0098214
H	3.0415788	2.8673771	0.7963548
H	3.8699720	1.2834526	0.9118560
C	-0.8812893	0.9684953	2.6042000
C	-2.1467372	1.5939732	2.5040123
H	-2.2698891	2.3944648	1.7711444
C	-3.1733787	1.1631113	3.3110091
H	-4.1544500	1.6424097	3.2544448
C	-2.9913364	0.0682939	4.2029341
C	-4.0697041	-0.4262137	4.9902518
H	-5.0366818	0.0806529	4.9244860
C	-3.9114184	-1.5260678	5.8061718
H	-4.7501317	-1.8976433	6.4006825
C	-2.6592600	-2.1860647	5.8585540
H	-2.5401869	-3.0740435	6.4851000
C	-1.5884062	-1.7222213	5.1212411
H	-0.6359201	-2.2506397	5.1672460
C	-1.7061855	-0.5724206	4.2865924
C	-0.6117517	-0.0630314	3.4974787
C	0.7693699	-0.6253904	3.5618250
C	1.3862940	-1.0620210	2.3907962
C	2.6618837	-1.6799572	2.3938054
H	3.0659260	-2.0308175	1.4419845
C	3.3502740	-1.8315811	3.5735404
H	4.3289020	-2.3193531	3.5834768
C	2.8170898	-1.3287788	4.7929599
C	3.5528807	-1.4122310	6.0087365
H	4.5291565	-1.9052811	5.9942423
C	3.0601548	-0.8719159	7.1770093

H	3.6378668	-0.9369509	8.1026267
C	1.8064187	-0.2128452	7.1702159
H	1.4277240	0.2406444	8.0900570
C	1.0598954	-0.1306840	6.0122040
H	0.1023946	0.3903175	6.0255971
C	1.5235037	-0.6999196	4.7899436
Br	-1.5657343	2.8086642	-1.2885350
N	-0.2816267	-1.9557388	-4.9066138
H	-0.5576556	-2.9309513	-4.8998667
N	1.9482862	1.2364870	0.0195333
O	-0.4013340	-0.0377351	-3.6582481
O	0.1263594	1.4282593	1.7739604
O	0.7542387	-0.9399002	1.1713132
P	0.4874167	0.5463383	0.4139059
Pd	-1.0218631	0.3441927	-1.2082739

7a-TS1
(-95.53 cm^{-1})

C	-1.2862833	-0.9897576	-0.9094039
H	-0.8390484	-1.8174507	-0.3506219
C	-2.3962893	-0.1226816	-0.5171421
H	-2.7479555	0.2449559	0.4495179
C	-2.8040446	0.0741367	-1.8177373
H	-3.5551261	0.7015414	-2.3000637
C	-1.9255300	-1.0723319	-2.3456945
H	-2.5117851	-1.9989500	-2.4868537
C	-1.0235373	-0.7817911	-3.5329755
C	-0.5473638	-1.5423667	-5.8786469
C	-0.9038418	-2.5163308	-6.8256958
H	-1.6540807	-3.2728885	-6.5736501
C	-0.3121571	-2.5293144	-8.0890273
H	-0.6095586	-3.2980904	-8.8080316
C	0.6517384	-1.5768983	-8.4500436
C	0.9991109	-0.6121280	-7.4903382
H	1.7480461	0.1454519	-7.7404857
C	0.4199193	-0.5802666	-6.2215288
H	0.7049794	0.1792780	-5.4973388
C	1.2932550	-1.5736679	-9.8162561
H	0.9595522	-2.4270789	-10.4259471
H	2.3927134	-1.6211855	-9.7417026
H	1.0492473	-0.6502027	-10.3691144
C	3.3204490	0.9983139	-1.0424053
H	4.0741025	0.2389164	-1.3184721
H	3.7885896	1.9969985	-1.0791661
H	2.5107229	0.9846741	-1.7849799
C	3.7655093	0.7544529	1.3790950
H	3.2732866	0.5926366	2.3462386
H	4.2756013	1.7335500	1.4166991
H	4.5287953	-0.0316275	1.2357080
C	-0.3214833	1.3078405	2.4693761
C	-1.2772313	2.2986556	2.1419186
H	-0.9675864	3.1136619	1.4842751
C	-2.5601506	2.1751044	2.6223498
H	-3.3109095	2.9331808	2.3835212
C	-2.9444921	1.0421679	3.3957944
C	-4.2881967	0.8691793	3.8341966
H	-5.0185908	1.6478833	3.5964405
C	-4.6707834	-0.2569325	4.5317538
H	-5.7070433	-0.3775418	4.8582347
C	-3.7187540	-1.2680597	4.8133239
H	-4.0282072	-2.1707927	5.3466207
C	-2.4047850	-1.1256575	4.4162393
H	-1.6859010	-1.9160541	4.6337974
C	-1.9678544	0.0346211	3.7134726
C	-0.6088932	0.2103278	3.2720697
C	0.4857912	-0.7494054	3.5932765
C	1.2257036	-1.3204006	2.5601367
C	2.2486993	-2.2705405	2.8014619
H	2.7687011	-2.6915775	1.9388241
C	2.5537716	-2.6412744	4.0891314
H	3.3337040	-3.3833662	4.2800326
C	1.8811751	-2.0465752	5.1931753
C	2.2259100	-2.3784456	6.5337191
H	3.0033504	-3.1292802	6.7023209
C	1.6078038	-1.7616289	7.6004060
H	1.8844830	-2.0225549	8.6251679
C	0.6221217	-0.7717142	7.3641113
H	0.1511741	-0.2635919	8.2097503
C	0.2556412	-0.4364783	6.0766643
H	-0.4970295	0.3349789	5.9145907
C	0.8517118	-1.0729335	4.9489661
Br	0.2409758	3.2873887	-1.3227723
N	-1.1913639	-1.5852119	-4.6212410
H	-1.8917186	-2.3113445	-4.5245238
N	2.7944676	0.7318405	0.2917242
O	-0.2221410	0.1480096	-3.4780014
O	0.9426717	1.4213269	1.9163890
O	0.9656768	-1.0137649	1.2436308
P	1.1743560	0.5045876	0.5400432
Pd	-0.3053159	0.8304512	-1.0730315

S-7a-INT1

C	4.6529547	2.3434367	2.1575473
H	5.4092366	2.5328823	2.9227772
C	3.2260774	2.2856102	2.2332092
H	2.4969188	2.5529517	2.9994232
C	3.1331687	1.7875402	0.9108183
H	2.3046205	1.4576417	0.2834957
C	4.6286809	1.3737323	0.9429718
H	4.7231283	0.3168049	1.2578711
C	5.4987944	1.5125717	-0.3079468
C	7.6250261	2.6059268	-1.0843371
C	8.6681485	3.4540681	-0.6651956
H	8.6510338	3.8682855	0.3479965
C	9.7190255	3.7670475	-1.5240852
H	10.5171888	4.4268344	-1.1695797
C	9.7743637	3.2490324	-2.8296873
C	8.7282517	2.4064162	-3.2311514

H	8.7382546	1.9857262	-4.2412254
C	7.6632969	2.0798588	-2.3861066
H	6.8624446	1.4239101	-2.7199959
C	10.9191149	3.5919981	-3.7526599
H	11.8883459	3.2816492	-3.3259776
H	10.9817612	4.6790121	-3.9330871
H	10.8109167	3.0974325	-4.7294149
C	5.8101972	6.2023653	-1.2759862
H	6.8751899	6.1307867	-1.5592973
H	5.2895019	6.8515557	-1.9996966
H	5.3571934	5.2047393	-1.3447733
C	6.1570439	8.1065385	0.2504250
H	5.9582440	8.4650520	1.2670074
H	5.6440689	8.7812315	-0.4574158
H	7.2433698	8.1637752	0.0565800
C	3.9122201	6.6458398	3.4876016
C	2.5355020	6.3866240	3.6884812
H	1.8613324	6.4790476	2.8346848
C	2.0904697	6.0300317	4.9396237
H	1.0274400	5.8417945	5.1128950
C	3.0053141	5.8691444	6.0183738
C	2.5632486	5.4288502	7.2979561
H	1.4943614	5.2509158	7.4467175
C	3.4570226	5.2145821	8.3251736
H	3.1043351	4.8701709	9.3007109
C	4.8403298	5.4262705	8.1071669
H	5.5520553	5.2315592	8.9137874
C	5.3001326	5.8710371	6.8841073
H	6.3697287	6.0192631	6.7335143
C	4.4042983	6.1265376	5.8051751
C	4.8507375	6.5827984	4.5116271
C	6.2704914	6.9436126	4.2231131
C	6.9394855	6.3188351	3.1723162
C	8.3080773	6.5642336	2.8978631
H	8.7782421	6.0162732	2.0789603
C	9.0116217	7.4674965	3.6582026
H	10.0713202	7.6491507	3.4594662
C	8.3671636	8.2019856	4.6921904
C	9.0666251	9.1923557	5.4382559
H	10.1267070	9.3548135	5.2233114
C	8.4252471	9.9436803	6.3995380
H	8.9721288	10.7052251	6.9612674
C	7.0456255	9.7373297	6.6447200
H	6.5292108	10.3516972	7.3868868
C	6.3430684	8.7727612	5.9510021
H	5.2794529	8.6377733	6.1477114
C	6.9771830	7.9581108	4.9677896
Br	2.2974385	4.8756675	-0.8365618
N	6.5949491	2.3299756	-0.1617927
H	6.6663076	2.8222537	0.7222402
N	5.6649073	6.7433305	0.0741697
O	5.2278279	0.8830348	-1.3135026
O	4.3178708	6.9835585	2.2077279
O	6.2926024	5.3966832	2.3754766
P	5.0499175	5.8006359	1.2934028
Pd	3.8301931	3.8807474	0.8678920

7a-INT1

C	4.5861272	2.6925309	2.6389933
H	5.2871485	3.0741542	3.3850562
C	3.1592383	2.5797670	2.6492962
H	2.3774041	2.9828899	3.2956419
C	3.1656520	1.8097041	1.4663469
H	2.3934906	1.3282462	0.8655186
C	4.6781769	1.5045860	1.6394383
H	4.8315923	0.5286795	2.1375963
C	5.5690676	1.5425590	0.3937624
C	7.5150382	2.8293025	-0.5321461
C	8.4924162	3.7815514	-0.1867344
H	8.5561877	4.1240277	0.8477999
C	9.3528402	4.2984974	-1.1510299
H	10.1004279	5.0418932	-0.8560477
C	9.2770649	3.8853792	-2.4928193
C	8.3086751	2.9247914	-2.8169595
H	8.2257898	2.5768812	-3.8509318
C	7.4351714	2.3920581	-1.8638891
H	6.6893031	1.6501132	-2.1400522
C	10.2100373	4.4601409	-3.5306832
H	11.2658871	4.2827226	-3.2636667
H	10.0818597	5.5523286	-3.6246620
H	10.0359212	4.0168974	-4.5226097
C	5.8566299	5.7877531	-1.5808145
H	6.8681374	5.4867398	-1.9000376
H	5.4359232	6.4961898	-2.3138753
H	5.2093969	4.9023835	-1.5649467
C	6.7351084	7.6108824	-0.1567545
H	6.6541037	8.0584481	0.8422582
H	6.4029577	8.3605529	-0.8952828
H	7.7947574	7.3690108	-0.3570678
C	4.1037107	6.6671818	3.1343207
C	2.7108837	6.4390052	3.2330823
H	2.0979665	6.5889941	2.3425385
C	2.1805086	6.0147515	4.4291075
H	1.1051414	5.8414260	4.5231334
C	3.0229052	5.7570281	5.5480372
C	2.4948690	5.2464391	6.7675645
H	1.4151786	5.0887194	6.8433840
C	3.3209005	4.9429829	7.8289784
H	2.9021230	4.5464213	8.7574285
C	4.7199276	5.1334871	7.7095008
H	5.3756060	4.8725680	8.5442961
C	5.2631302	5.6423502	6.5473375
H	6.3421455	5.7787298	6.4687048
C	4.4385449	5.9863066	5.4374027
C	4.9736218	6.5024978	4.2048028
C	6.4167049	6.8208018	4.0105577
C	7.1208655	6.2185969	2.9705930
C	8.4975518	6.4725679	2.7513050
H	8.9930890	5.9667596	1.9217843
C	9.1774084	7.3406953	3.5718354

H	10.2427556	7.5282038	3.4126208
C	8.5032774	8.0293408	4.6183060
C	9.1833141	8.9709101	5.4411162
H	10.2521734	9.1330779	5.2751910
C	8.5129628	9.6753146	6.4182840
H	9.0457533	10.3980853	7.0413286
C	7.1228503	9.4720603	6.6013846
H	6.5857547	10.0494175	7.3583463
C	6.4369348	8.5574711	5.8283470
H	5.3654727	8.4237487	5.9775397
C	7.1017873	7.7895753	4.8281243
Br	2.4272506	4.4742007	-0.9602968
N	6.6406445	2.4020318	0.4855583
H	6.7153823	2.9335835	1.3470255
N	5.9032078	6.4168851	-0.2597818
O	5.3126332	0.8469117	-0.5701511
O	4.6191438	7.0045122	1.8912175
O	6.5058696	5.3160219	2.1286951
P	5.2322777	5.6907926	1.0607307
Pd	3.8439947	3.8799849	1.0017673

S-7a-TS2
(-82.52 cm^{-1})

C	0.1322077	-3.9327702	0.9301304
H	0.0239940	-4.1106085	2.0022518
C	-0.7430902	-3.6734757	-0.0947040
H	-1.8323286	-3.6287513	-0.1566954
C	0.3201186	-3.4713088	-1.0783099
H	0.3160496	-3.7246317	-2.1424476
C	1.3372175	-4.0342560	-0.0090455
H	1.6160527	-5.0853849	-0.2060047
C	2.5543593	-3.1787313	0.2979332
C	5.0512803	-3.3321000	0.5497802
C	6.1271758	-4.2170089	0.3736843
H	5.9372494	-5.2506287	0.0665865
C	7.4384525	-3.7925616	0.5864020
H	8.2566217	-4.5042256	0.4422340
C	7.7234949	-2.4760356	0.9777646
C	6.6356261	-1.6055009	1.1530025
H	6.8213453	-0.5726402	1.4638845
C	5.3161438	-2.0102989	0.9473156
H	4.4911246	-1.3168210	1.0916470
C	9.1412951	-2.0026828	1.1876418
H	9.8500982	-2.8443328	1.2110553
H	9.2449647	-1.4454337	2.1330456
H	9.4600071	-1.3232135	0.3777952
C	2.2609954	1.5154262	-0.2570305
H	2.9695254	1.8458473	0.5253677
H	2.5517880	1.9909357	-1.2106567
H	2.3593923	0.4267809	-0.3694833
C	0.6468741	3.3023975	0.2611351
H	-0.4229688	3.4975182	0.3984668
H	0.9804930	3.8456249	-0.6411914
H	1.1981815	3.7062241	1.1301794
C	-2.8355334	0.9977892	0.1700992
C	-3.5545425	0.3254225	-0.8472500
H	-3.0854865	0.2240706	-1.8281189
C	-4.7989851	-0.1872665	-0.5672393
H	-5.3714071	-0.6975491	-1.3467407
C	-5.3474791	-0.0960314	0.7428103
C	-6.6064139	-0.6822264	1.0561890
H	-7.1651177	-1.1740738	0.2546295
C	-7.1089998	-0.6487945	2.3392225
H	-8.0752077	-1.1070696	2.5662630
C	-6.3586691	-0.0320902	3.3700926
H	-6.7437326	-0.0291891	4.3934446
C	-5.1421738	0.5601482	3.0968332
H	-4.5750608	1.0198842	3.9065715
C	-4.6029059	0.5709555	1.7771964
C	-3.3355149	1.1794347	1.4552439
C	-2.5264794	1.9443403	2.4495139
C	-1.2039038	1.5729668	2.6869822
C	-0.4139915	2.1987564	3.6845658
H	0.6000326	1.8262187	3.8444429
C	-0.9308954	3.2356611	4.4236562
H	-0.3297212	3.7130598	5.2025147
C	-2.2417555	3.7254320	4.1665223
C	-2.7612839	4.8483695	4.8705878
H	-2.1415541	5.3108867	5.6443989
C	-4.0087812	5.3568044	4.5786833
H	-4.3936742	6.2235115	5.1223831
C	-4.7861624	4.7610983	3.5554876
H	-5.7642292	5.1800468	3.3041047
C	-4.3190416	3.6599556	2.8669183
H	-4.9281446	3.2225408	2.0754095
C	-3.0448885	3.0898932	3.1570324
Br	-0.3743056	-0.8660618	-2.9046548
N	3.7486105	-3.8328448	0.3229204
H	3.7062488	-4.8228433	0.1088216
N	0.8917640	1.8746848	0.0938240
O	2.4251838	-1.9720582	0.5062691
O	-1.5921754	1.5071045	-0.1559054
O	-0.6182753	0.5516421	1.9739454
P	-0.2350746	0.6674538	0.3290488
Pd	0.1831835	-1.4864354	-0.5501744

7a-TS2
(-78.55 cm^{-1})

C	-0.0224155	-3.9496197	0.9323765
H	-0.1476642	-4.1292346	2.0020038
C	-0.8746294	-3.6534967	-0.0995015
H	-1.9617605	-3.5732890	-0.1722193
C	0.2048854	-3.4699686	-1.0726404
H	0.2079887	-3.7376009	-2.1338706
C	1.1964983	-4.0580955	0.0140936
H	1.4765746	-5.1076377	-0.1878738
C	2.4029940	-3.2005990	0.3567829
C	4.9003358	-3.2862962	0.5715523

C	6.0020121	-4.1301483	0.3564722
H	5.8421801	-5.1625030	0.0287120
C	7.3022518	-3.6647604	0.5511662
H	8.1415167	-4.3434646	0.3736876
C	7.5494252	-2.3472174	0.9635285
C	6.4368727	-1.5189470	1.1811977
H	6.5946780	-0.4868423	1.5092646
C	5.1280280	-1.9644348	0.9938846
H	4.2844776	-1.3018532	1.1697452
C	8.9526998	-1.8246873	1.1501568
H	9.6950080	-2.6374001	1.1401485
H	9.0561771	-1.2813556	2.1036931
H	9.2221581	-1.1178061	0.3460751
C	2.3582212	1.4339978	-0.1865577
H	3.0584391	1.7117645	0.6234677
H	2.6856592	1.9333175	-1.1157311
H	2.4246819	0.3472828	-0.3367171
C	0.7822586	3.2475620	0.3841602
H	-0.2838690	3.4586538	0.5305549
H	1.1316919	3.8281392	-0.4882675
H	1.3358489	3.5918827	1.2769299
C	-2.7428164	0.9958810	0.1538964
C	-3.4360196	0.3031830	-0.8661824
H	-2.9548919	0.2077440	-1.8415129
C	-4.6667698	-0.2445586	-0.5884484
H	-5.2197601	-0.7750590	-1.3685307
C	-5.2247279	-0.1626492	0.7186261
C	-6.4703548	-0.7776436	1.0297625
H	-7.0130443	-1.2895462	0.2297357
C	-6.9813429	-0.7448307	2.3097332
H	-7.9374732	-1.2247812	2.5345367
C	-6.2547776	-0.0992955	3.3408177
H	-6.6489115	-0.0945532	4.3606223
C	-5.0510814	0.5190798	3.0706919
H	-4.5005973	1.0035431	3.8776675
C	-4.5045684	0.5283512	1.7543983
C	-3.2519059	1.1616865	1.4367374
C	-2.4542268	1.9323258	2.4334813
C	-1.1329934	1.5633296	2.6767270
C	-0.3367904	2.2127654	3.6519935
H	0.6806615	1.8481413	3.8069813
C	-0.8526691	3.2644449	4.3715235
H	-0.2468701	3.7627745	5.1334200
C	-2.1700158	3.7391065	4.1180586
C	-2.6966643	4.8650268	4.8119763
H	-2.0754769	5.3471966	5.5725124
C	-3.9554787	5.3504087	4.5285569
H	-4.3466089	6.2182993	5.0658419
C	-4.7383795	4.7288502	3.5242473
H	-5.7270850	5.1281253	3.2829322
C	-4.2632515	3.6267571	2.8433284
H	-4.8757582	3.1662243	2.0677358
C	-2.9769498	3.0814887	3.1259832
Br	-0.4208638	-0.8485242	-2.9125784
N	3.6110000	-3.8244088	0.3515591
H	3.5941878	-4.8075193	0.1046469
N	0.9943792	1.8248357	0.1482682
O	2.2522378	-2.0039296	0.6131894
O	-1.5008384	1.5234942	-0.1525221
O	-0.5539178	0.5278671	1.9785275
P	-0.1682717	0.6493292	0.3372595
Pd	0.1336597	-1.4773531	-0.5641963

S-7a-INT2

C	-1.4269562	-0.8803547	-5.3590506
H	-1.9889970	-0.1394428	-4.7835226
C	-1.3053020	-1.1717811	-6.6727894
H	-1.7603054	-0.7638118	-7.5788281
C	-0.3190387	-2.2724199	-6.4759066
H	-0.5816454	-3.2811031	-6.8280071
C	-0.4639473	-1.9889109	-4.9270574
H	-0.9200704	-2.8006569	-4.3302438
C	0.8344883	-1.5295696	-4.3091134
C	1.9029652	-0.8480602	-2.1408061
C	1.6513375	-0.8040738	-0.7582347
H	0.6817553	-1.1339237	-0.3710317
C	2.6248895	-0.3456110	0.1251102
H	2.4022714	-0.3224389	1.1960948
C	3.8813888	0.0841180	-0.3350511
C	4.1147795	0.0312761	-1.7162173
H	5.0816647	0.3585355	-2.1095330
C	3.1519945	-0.4250443	-2.6214850
H	3.3675538	-0.4512568	-3.6875545
C	4.9264626	0.5935575	0.6274592
H	5.0928284	-0.1157534	1.4551518
H	4.6189895	1.5515038	1.0818571
H	5.8912215	0.7593015	0.1252135
C	4.5512007	-3.7312562	-8.1319945
H	5.3455047	-4.1991010	-7.5209285
H	4.4811123	-4.2761358	-9.0897712
H	3.5905550	-3.8481404	-7.6099403
C	6.0470868	-2.0437947	-9.1226376
H	6.1382840	-0.9711662	-9.3294262
H	6.0194371	-2.5771659	-10.0897873
H	6.9443930	-2.3786168	-8.5702381
C	3.4791745	1.2483805	-8.7316252
C	2.2317140	1.5985370	-9.3018836
H	1.7066856	0.8471347	-9.8953079
C	1.7185266	2.8547299	-9.0819992
H	0.7611895	3.1435391	-9.5243869
C	2.4008630	3.7849044	-8.2483916
C	1.8421091	5.0620974	-7.9599692
H	0.8894870	5.3308370	-8.4257582
C	2.4714935	5.9385701	-7.1023072
H	2.0277680	6.9138857	-6.8856107
C	3.6903602	5.5632513	-6.4863748
H	4.1764033	6.2466909	-5.7848567
C	4.2678703	4.3394851	-6.7580238
H	5.2009880	4.0644772	-6.2653334
C	3.6605425	3.4172752	-7.6593464

C	4.2319709	2.1283551	-7.9615249
C	5.5614467	1.6857800	-7.4466727
C	5.6564309	0.4941446	-6.7291397
C	6.8743451	0.0636035	-6.1440320
H	6.8654120	-0.8614569	-5.5637535
C	8.0196004	0.8049662	-6.3095595
H	8.9582893	0.4816467	-5.8508411
C	8.0106901	1.9835717	-7.1064514
C	9.2052543	2.7184046	-7.3497315
H	10.1322153	2.3757179	-6.8808037
C	9.2047414	3.8268262	-8.1689110
H	10.1303149	4.3784688	-8.3530130
C	7.9995958	4.2393174	-8.7883264
H	8.0033491	5.1022311	-9.4597001
C	6.8209265	3.5586923	-8.5593458
H	5.9067127	3.8859925	-9.0550027
C	6.7759663	2.4225158	-7.6990117
Br	0.9660004	-2.4071005	-9.3966941
N	0.8576524	-1.3206094	-2.9734725
H	-0.0104207	-1.5265883	-2.4908754
N	4.8307150	-2.3204381	-8.3682822
O	1.8345356	-1.3313602	-5.0267457
O	3.9593231	-0.0248946	-8.9750086
O	4.5527218	-0.3029645	-6.5322230
P	3.7847802	-1.1674221	-7.7698736
Pd	1.5820363	-1.8030411	-7.0888780

7a-INT2

C	-0.9361346	0.0980765	-6.1577933
H	-1.2372689	1.1092409	-5.8713404
C	-0.9771917	-0.6229545	-7.2996181
H	-1.3588684	-0.4277751	-8.3045201
C	-0.2824532	-1.8159432	-6.7356335
H	-0.7993421	-2.7874618	-6.7514465
C	-0.2537017	-1.0192108	-5.3645049
H	-0.8557510	-1.4482166	-4.5416975
C	1.1559635	-0.7462954	-4.9017789
C	2.5623967	0.4346548	-3.2121790
C	2.4780786	1.3946051	-2.1902600
H	1.4979288	1.7319999	-1.8387121
C	3.6332139	1.9465754	-1.6424304
H	3.5407034	2.7016025	-0.8559148
C	4.9083944	1.5644630	-2.0935710
C	4.9737370	0.5837806	-3.0921222
H	5.9473758	0.2631588	-3.4727350
C	3.8270545	0.0139177	-3.6504944
H	3.9162219	-0.7247669	-4.4413128
C	6.1536184	2.2128336	-1.5407647
H	6.1340682	2.2543167	-0.4393057
H	6.2514700	3.2512195	-1.9028906
H	7.0610912	1.6719457	-1.8480578
C	4.3991808	-4.1263388	-9.3675919
H	5.0199047	-4.9034973	-8.8844874
H	4.4507716	-4.2679540	-10.4613900
H	3.3505360	-4.2593261	-9.0663220
C	6.2136261	-2.4337283	-9.4178488
H	6.4341253	-1.3913776	-9.1520188
H	6.3235724	-2.5352514	-10.5122990
H	6.9602312	-3.0888000	-8.9319753
C	3.4069410	0.7083395	-8.3648722
C	2.1301110	1.0167255	-8.8935773
H	1.7824601	0.4535203	-9.7611391
C	1.3535845	1.9654749	-8.2760536
H	0.3607688	2.2032195	-8.6656555
C	1.8030463	2.6097424	-7.0900489
C	0.9664299	3.5281907	-6.3954470
H	-0.0181956	3.7545809	-6.8150525
C	1.3769337	4.1165829	-5.2175407
H	0.7231589	4.8191815	-4.6937491
C	2.6495533	3.7997042	-4.6839311
H	2.9698649	4.2393850	-3.7366928
C	3.4869412	2.9189042	-5.3371786
H	4.4471421	2.6678481	-4.8910144
C	3.1052943	2.3025013	-6.5628758
C	3.9424479	1.3520582	-7.2554453
C	5.3193592	0.9989902	-6.8052470
C	5.6723296	-0.3314099	-6.5768310
C	6.9451905	-0.6912789	-6.0641365
H	7.1344613	-1.7506849	-5.8804716
C	7.8840294	0.2731996	-5.7915670
H	8.8572626	-0.0041012	-5.3771343
C	7.6152578	1.6411197	-6.0744633
C	8.5988032	2.6462093	-5.8556581
H	9.5610393	2.3496135	-5.4279872
C	8.3575707	3.9619445	-6.1885726
H	9.1227822	4.7241382	-6.0200788
C	7.1178890	4.3191084	-6.7736809
H	6.9364275	5.3561498	-7.0684320
C	6.1385013	3.3699621	-6.9836308
H	5.1959887	3.6650862	-7.4443437
C	6.3363475	2.0059047	-6.6192370
Br	0.7779366	-2.8883685	-9.5739452
N	1.3461440	-0.0381628	-3.7685996
H	0.5019978	0.2846222	-3.3085641
N	4.8619292	-2.7904104	-9.0103676
O	2.1198115	-1.1567797	-5.5772383
O	4.1022407	-0.3378606	-8.9484234
O	4.7958274	-1.3710644	-6.7463416
P	3.8996757	-1.7841807	-8.1130717
Pd	1.6378445	-2.0243422	-7.4360039

S-7b

C	6.2225226	0.1464635	2.0886794
H	5.6485999	0.0583174	3.0233993
C	7.0426717	-1.0329567	1.6721604
H	7.8728051	-1.5530197	2.1589856
C	6.3915916	-1.2323134	0.5067500
H	6.4954618	-1.9390841	-0.3213801

C	5.4110066	-0.0847449	0.7519773
H	4.3576950	-0.3932488	0.8864974
C	5.5098817	1.0886464	-0.1940595
C	4.7128147	1.9576733	-2.4114914
C	3.8872629	1.6145105	-3.4931905
H	3.3402726	0.6661505	-3.4823630
C	3.7561315	2.4724014	-4.5855533
H	3.1061069	2.1806432	-5.4154019
C	4.4407015	3.6956466	-4.6352545
C	5.2614204	4.0223120	-3.5431502
H	5.8091843	4.9694650	-3.5472838
C	5.4078119	3.1795960	-2.4406501
H	6.0510898	3.4633875	-1.6102850
C	4.3091725	4.6303032	-5.8131916
H	3.6018215	4.2426977	-6.5616141
H	5.2802434	4.7813432	-6.3150625
H	3.9542574	5.6257521	-5.4971987
C	10.5244348	1.4999885	2.9570516
H	11.2047501	0.6445263	3.1179913
H	11.1244417	2.3942348	2.7150963
H	9.8843896	1.2847984	2.0902768
C	10.4680256	2.0432128	5.3561091
H	9.7969345	2.2429394	6.1988322
H	11.0908125	2.9416140	5.1947179
H	11.1325679	1.2024978	5.6232055
C	6.4639369	3.2158795	5.5747543
C	5.7336251	4.2894412	5.0127882
H	6.2250706	4.9065608	4.2575987
C	4.4393358	4.5068485	5.4224999
H	3.8640476	5.3385746	5.0068233
C	3.8150014	3.6372717	6.3609723
C	2.4535868	3.8162404	6.7369235
H	1.9052063	4.6640157	6.3162737
C	1.8298689	2.9377524	7.5967806
H	0.7823478	3.0850790	7.8725537
C	2.5474974	1.8295472	8.1094899
H	2.0453104	1.1167465	8.7691107
C	3.8737721	1.6368042	7.7793680
H	4.4056248	0.7716994	8.1763685
C	4.5603636	2.5389876	6.9153418
C	5.9433030	2.3668591	6.5448436
C	6.8161349	1.3014915	7.1193456
C	7.4822594	0.4249340	6.2647678
C	8.2679741	-0.6510281	6.7474915
H	8.7301982	-1.3185199	6.0176101
C	8.4249966	-0.8351699	8.1002194
H	9.0206199	-1.6704818	8.4785919
C	7.8441778	0.0742651	9.0273293
C	8.0619366	-0.0618540	10.4275683
H	8.6606581	-0.9058625	10.7820686
C	7.5476113	0.8538929	11.3199788
H	7.7274903	0.7415919	12.3923073
C	6.7978670	1.9554250	10.8395474
H	6.4123217	2.6969870	11.5442636
C	6.5543509	2.1068042	9.4895010
H	5.9845596	2.9678638	9.1400031
C	7.0475174	1.1669432	8.5374768
Br	8.2094815	4.2935144	1.7509121
N	4.7916337	1.0346365	-1.3375868
H	4.2277514	0.1994190	-1.4539378
N	9.7117448	1.7295438	4.1471500
O	6.2485992	2.0518170	0.0827877
O	7.7639010	3.0290604	5.1358653
O	7.3723546	0.5537998	4.8962614
P	8.0567163	1.8271853	4.0331992
Pd	7.1671061	1.9832921	2.0115660

7b

C	6.9555878	-0.4526088	1.5997808
H	7.8453297	-1.0819044	1.4510628
C	5.8571534	-0.9517140	2.4753297
H	5.8523710	-1.2427807	3.5285999
C	4.9050887	-0.9003834	1.5164566
H	3.8309865	-1.1028953	1.4830008
C	5.8828536	-0.4245406	0.4382679
H	6.0545662	-1.1365597	-0.3898232
C	5.6423949	0.9684336	-0.0873916
C	4.4389750	2.2802715	-1.8496489
C	3.3567769	2.1752825	-2.7364965
H	2.8584769	1.2108215	-2.8763958
C	2.9049114	3.2930462	-3.4391106
H	2.0591575	3.1839322	-4.1238265
C	3.5100770	4.5476877	-3.2768638
C	4.5947548	4.6339572	-2.3889819
H	5.0918055	5.5972814	-2.2410805
C	5.0667410	3.5276064	-1.6826293
H	5.9109264	3.6303421	-1.0048077
C	3.0266589	5.7664589	-4.0235813
H	2.1636917	5.5360394	-4.6662076
H	3.8228455	6.1819819	-4.6643983
H	2.7258575	6.5665905	-3.3264731
C	10.5491215	3.6118732	1.7983693
H	11.3476478	3.8898172	2.5105737
H	10.9375834	3.7487352	0.7729381
H	10.3191388	2.5484498	1.9535138
C	9.5790809	5.8730115	1.8750446
H	8.6371720	6.4200226	1.9974949
H	9.9842565	6.0988663	0.8722503
H	10.2970728	6.2377635	2.6312798
C	5.6424246	5.0119397	2.5875319
C	4.5056322	4.4830620	1.9316600
H	4.6556936	3.9453602	0.9971502
C	3.2589360	4.6521682	2.4858069
H	2.3741887	4.2590083	1.9774331
C	3.1016202	5.3079606	3.7382138
C	1.8219643	5.4401412	4.3477363
H	0.9479681	5.0557254	3.8138008
C	1.6812547	6.0241397	5.5884147
H	0.6931913	6.1151935	6.0471254
C	2.8274404	6.4903486	6.2786462

H	2.7199522	6.9283547	7.2745034
C	4.0805241	6.3897457	5.7097071
H	4.9512393	6.7437420	6.2619259
C	4.2617817	5.8191579	4.4160985
C	5.5539158	5.7065186	3.7897630
C	6.7941176	6.2476556	4.4162476
C	7.8602054	5.3840027	4.6504866
C	9.0360856	5.7975997	5.3223275
H	9.8129072	5.0511514	5.4988399
C	9.1682343	7.1051828	5.7261030
H	10.0686717	7.4320884	6.2532915
C	8.1515760	8.0597483	5.4398000
C	8.3040473	9.4307294	5.7911622
H	9.2136108	9.7370052	6.3159253
C	7.3370790	10.3596154	5.4701574
H	7.4691039	11.4100601	5.7423565
C	6.1741156	9.9510644	4.7710304
H	5.4184682	10.6920726	4.4968508
C	5.9884040	8.6272873	4.4285135
H	5.0910399	8.3298668	3.8850673
C	6.9554188	7.6350562	4.7637659
Br	9.0771627	0.5633168	3.7392500
N	4.8551003	1.1030174	-1.1782359
H	4.4666690	0.2387073	-1.5401458
N	9.3615477	4.4357393	2.0035894
O	6.1395819	1.9561400	0.4897871
O	6.8694172	4.8400059	1.9743266
O	7.8003915	4.0793539	4.2161445
P	7.9651788	3.7244774	2.5821660
Pd	7.5031473	1.4718510	2.0817774

S-7b-INT2

C	6.8363078	-0.3988740	1.6480572
H	7.7337267	-1.0254735	1.5428924
C	5.7168696	-0.8845544	2.5043576
H	5.6803111	-1.1542003	3.5628236
C	4.7954863	-0.8712017	1.5156788
H	3.7269710	-1.0964080	1.4555156
C	5.8003031	-0.4147647	0.4552112
H	6.0039540	-1.1523669	-0.3427809
C	5.5735670	0.9586200	-0.1274193
C	4.4792754	2.1621107	-2.0457636
C	3.5757464	1.9480265	-3.0981051
H	3.1794216	0.9438703	-3.2795209
C	3.1752836	3.0046421	-3.9159954
H	2.4700175	2.8088623	-4.7286755
C	3.6581191	4.3058914	-3.7132941
C	4.5623234	4.5018952	-2.6566692
H	4.9611413	5.5036779	-2.4706106
C	4.9771153	3.4587044	-1.8276064
H	5.6820123	3.6434997	-1.0202560
C	3.2307891	5.4560632	-4.5922642
H	2.5089205	5.1348020	-5.3578178
H	4.0941333	5.9057650	-5.1115805
H	2.7589900	6.2589860	-4.0010313
C	10.3522516	3.6278163	1.5244594
H	11.2388473	3.8792223	2.1350867
H	10.6243636	3.7424244	0.4594064
H	10.1017121	2.5750743	1.7167530
C	9.4814494	5.9211594	1.6843058
H	8.5745216	6.5036683	1.8819071
H	9.7971644	6.1174482	0.6435889
H	10.2841441	6.2725193	2.3579629
C	5.5675505	5.1587819	2.7354737
C	4.3904348	4.6760344	2.1135830
H	4.4821795	4.1843327	1.1447460
C	3.1749418	4.8304184	2.7365014
H	2.2604047	4.4720733	2.2559941
C	3.0893845	5.4225860	4.0267608
C	1.8444062	5.5251033	4.7095818
H	0.9418790	5.1712342	4.2028024
C	1.7725254	6.0403242	5.9859203
H	0.8109381	6.1073148	6.5015283
C	2.9563582	6.4650542	6.6372235
H	2.9054760	6.8458832	7.6607324
C	4.1765601	6.3949765	5.9960258
H	5.0768211	6.7146008	6.5209812
C	4.2875073	5.8949444	4.6655051
C	5.5468870	5.8078246	3.9668303
C	6.8193304	6.3293088	4.5462351
C	7.9043427	5.4664329	4.6801063
C	9.1048568	5.8561604	5.3250962
H	9.8931094	5.1089866	5.4371145
C	9.2441840	7.1400708	5.7955401
H	10.1625186	7.4449366	6.3049237
C	8.2113217	8.1003143	5.6027496
C	8.3738429	9.4525777	6.0166715
H	9.3042016	9.7360387	6.5172686
C	7.3925299	10.3920748	5.7827976
H	7.5327336	11.4283894	6.1012398
C	6.2042477	10.0123551	5.1118448
H	5.4361443	10.7621243	4.9037335
C	6.0080130	8.7055452	4.7130476
H	5.0904596	8.4343326	4.1900242
C	6.9896561	7.7009875	4.9574426
Br	8.9383410	0.6645930	3.7596507
N	4.8542991	1.0370810	-1.2709266
H	4.5167071	0.1481953	-1.6243861
N	9.2264129	4.4955132	1.8583965
O	6.0279748	1.9696390	0.4448617
O	6.7590799	4.9937312	2.0538470
O	7.8319581	4.1787101	4.2028813
P	7.8598955	3.8291719	2.5599793
Pd	7.3624157	1.5345836	2.0834031

7b-INT2

C	6.9555878	-0.4526088	1.5997808
H	7.8453297	-1.0819044	1.4510628

C	5.8571534	-0.9517140	2.4753297
H	5.8523710	-1.2427807	3.5285999
C	4.9050887	-0.9003834	1.5164566
H	3.8309865	-1.1028953	1.4830008
C	5.8828536	-0.4245406	0.4382679
H	6.0545662	-1.1365597	-0.3898232
C	5.6423949	0.9684336	-0.0873916
C	4.4389750	2.2802715	-1.8496489
C	3.3567769	2.1752825	-2.7364965
H	2.8584769	1.2108215	-2.8763958
C	2.9049114	3.2930462	-3.4391106
H	2.0591575	3.1839322	-4.1238265
C	3.5100770	4.5476877	-3.2768638
C	4.5947548	4.6339572	-2.3889819
H	5.0918055	5.5972814	-2.2410805
C	5.0667410	3.5276064	-1.6826293
H	5.9109264	3.6303421	-1.0048077
C	3.0266589	5.7664589	-4.0235813
H	2.1636917	5.5360394	-4.6662076
H	3.8228455	6.1819819	-4.6643983
H	2.7258575	6.5665905	-3.3264731
C	10.5491215	3.6118732	1.7983693
H	11.3476478	3.8898172	2.5105737
H	10.9375834	3.7487352	0.7729381
H	10.3191388	2.5484498	1.9535138
C	9.5790809	5.8730115	1.8750446
H	8.6371720	6.4200226	1.9974949
H	9.9842565	6.0988663	0.8722503
H	10.2970728	6.2377635	2.6312798
C	5.6424246	5.0119397	2.5875319
C	4.5056322	4.4830620	1.9316600
H	4.6556936	3.9453602	0.9971502
C	3.2589360	4.6521682	2.4858069
H	2.3741887	4.2590083	1.9774331
C	3.1016202	5.3079606	3.7382138
C	1.8219643	5.4401412	4.3477363
H	0.9479681	5.0557254	3.8138008
C	1.6812547	6.0241397	5.5884147
H	0.6931913	6.1151935	6.0471254
C	2.8274404	6.4903486	6.2786462
H	2.7199522	6.9283547	7.2745034
C	4.0805241	6.3897457	5.7097071
H	4.9512393	6.7437420	6.2619259
C	4.2617817	5.8191579	4.4160985
C	5.5539158	5.7065186	3.7897630
C	6.7941176	6.2476556	4.4162476
C	7.8602054	5.3840027	4.6504866
C	9.0360856	5.7975997	5.3223275
H	9.8129072	5.0511514	5.4988399
C	9.1682343	7.1051828	5.7261030
H	10.0686717	7.4320884	6.2532915
C	8.1515760	8.0597483	5.4398000
C	8.3040473	9.4307294	5.7911622
H	9.2136108	9.7370052	6.3159253
C	7.3370790	10.3596154	5.4701574
H	7.4691039	11.4100601	5.7423565
C	6.1741156	9.9510644	4.7710304
H	5.4184682	10.6920726	4.4968508
C	5.9884040	8.6272873	4.4285135
H	5.0910399	8.3298668	3.8850673
C	6.9554188	7.6350562	4.7637659
Br	9.0771627	0.5633168	3.7392500
N	4.8551003	1.1030174	-1.1782359
H	4.4666690	0.2387073	-1.5401458
N	9.3615477	4.4357393	2.0035894
O	6.1395819	1.9561400	0.4897871
O	6.8694172	4.8400059	1.9743266
O	7.8003915	4.0793539	4.2161445
P	7.9651788	3.7244774	2.5821660
Pd	7.5031473	1.4718510	2.0817774

S-7b-TS2
(-97.14 cm^{-1})

C	1.9524840	-2.0144305	-2.4501303
H	2.9646135	-2.0934831	-2.8561261
C	1.2700953	-2.8932762	-1.5078798
H	1.6206174	-3.5100895	-0.6782036
C	0.0289560	-2.6489465	-2.0502008
H	-0.9895245	-2.9392558	-1.7865087
C	0.6131200	-1.9528217	-3.2842066
H	0.6355839	-2.6237630	-4.1624717
C	0.0812753	-0.5752936	-3.6438912
C	-0.8072514	0.7437376	-5.5907759
C	-1.1250821	0.6274408	-6.9559445
H	-0.9589222	-0.3229396	-7.4738371
C	-1.6469320	1.7108251	-7.6575822
H	-1.8826458	1.5918558	-8.7194956
C	-1.8726985	2.9469376	-7.0284084
C	-1.5474991	3.0460948	-5.6684307
H	-1.7050730	3.9957646	-5.1483934
C	-1.0224469	1.9712604	-4.9451809
H	-0.7761370	2.0762184	-3.8911335
C	-2.4545008	4.1112371	-7.7928296
H	-1.9218344	4.2763158	-8.7440792
H	-2.4025647	5.0431949	-7.2103192
H	-3.5152319	3.9360106	-8.0447399
C	2.4197273	2.8368511	0.4830057
H	3.1226829	3.1489077	1.2764791
H	2.1926911	3.7190380	-0.1427229
H	2.9246009	2.0798682	-0.1324344
C	0.4812067	3.2314093	1.9399217
H	-0.4704237	2.8050441	2.2755988
H	0.2637404	4.1664454	1.3924289
H	1.0909765	3.4874601	2.8253499
C	-1.7898035	-0.0822465	0.9931846
C	-2.5142348	-0.5324326	-0.1381833
H	-2.3483270	-0.0201510	-1.0877327
C	-3.3932111	-1.5823067	-0.0129056
H	-3.9735104	-1.9233088	-0.8749942
C	-3.5394875	-2.2625012	1.2285438

C	-4.3881766	-3.3991475	1.3511027
H	-4.9587989	-3.7201932	0.4747669
C	-4.4810665	-4.0940367	2.5378338
H	-5.1317562	-4.9688525	2.6164529
C	-3.7137569	-3.6800968	3.6542040
H	-3.7648544	-4.2471386	4.5874738
C	-2.8960582	-2.5710924	3.5741075
H	-2.3049833	-2.2755275	4.4410695
C	-2.7931308	-1.8111286	2.3723217
C	-1.9473812	-0.6481340	2.2554003
C	-1.2180688	-0.0627553	3.4188504
C	0.1616863	0.1168848	3.3441586
C	0.9243197	0.5759281	4.4475914
H	2.0072193	0.6476025	4.3280019
C	0.2982109	0.9064567	5.6251271
H	0.8822293	1.2492877	6.4837215
C	-1.1187988	0.8379542	5.7375963
C	-1.7843746	1.2509295	6.9260673
H	-1.1783925	1.5939862	7.7696394
C	-3.1599978	1.2368477	7.0139096
H	-3.6584460	1.5621503	7.9307726
C	-3.9280615	0.8146626	5.9015095
H	-5.0197081	0.8286056	5.9598749
C	-3.3127587	0.3915583	4.7405667
H	-3.9235205	0.0819436	3.8921347
C	-1.8921977	0.3672997	4.6203068
Br	4.0360899	-0.3394342	-0.6239692
N	-0.2813301	-0.3987240	-4.9446776
H	-0.1551079	-1.2057612	-5.5448456
N	1.1911362	2.3027403	1.0658132
O	-0.0038231	0.2980662	-2.7798749
O	-0.9334306	0.9812205	0.8111125
O	0.8458777	-0.1819644	2.1896770
P	0.7290380	0.7263251	0.7741258
Pd	1.5706045	-0.5873981	-1.0119230

7b-TS2
(-105.50 cm^{-1})

C	2.3444131	-2.0859159	-1.9010145
H	3.2986801	-2.3224405	-2.3806510
C	1.7370719	-2.6988402	-0.7216868
H	2.1575641	-3.1351468	0.1865151
C	0.4506300	-2.5088968	-1.1718608
H	-0.5452878	-2.6641813	-0.7530168
C	0.9247476	-2.1123168	-2.5810054
H	0.8035534	-2.9370893	-3.3080575
C	0.3946969	-0.8047060	-3.1330978
C	-1.4067964	0.1058411	-4.6140282
C	-2.5728999	-0.2883192	-5.2896916
H	-2.8118948	-1.3521242	-5.3880898
C	-3.4344365	0.6645951	-5.8345449
H	-4.3352727	0.3298666	-6.3566907
C	-3.1674988	2.0366638	-5.7200335
C	-1.9937765	2.4134859	-5.0473306
H	-1.7513808	3.4759524	-4.9485442
C	-1.1155075	1.4770822	-4.5011239
H	-0.2110248	1.7962765	-3.9888460
C	-4.1026980	3.0775524	-6.2843942
H	-4.8940696	2.6241922	-6.9003190
H	-3.5605028	3.8060375	-6.9097213
H	-4.5935575	3.6489994	-5.4775730
C	3.4774169	2.8555638	1.1014804
H	4.1486955	2.8457384	1.9792272
H	3.4805141	3.8741504	0.6734392
H	3.8842852	2.1520062	0.3618738
C	1.4942464	3.3098922	2.5035224
H	0.4547107	3.0031223	2.6725938
H	1.4904917	4.3648508	2.1761594
H	2.0434844	3.2468564	3.4606983
C	-1.1304983	0.7258040	0.4550490
C	-1.6356685	0.6437188	-0.8647486
H	-1.2494685	1.3404027	-1.6072227
C	-2.5694271	-0.3121343	-1.1847487
H	-2.9652524	-0.3706936	-2.2022917
C	-2.9971599	-1.2577230	-0.2103897
C	-3.8853797	-2.3182121	-0.5469498
H	-4.2635241	-2.3753581	-1.5720194
C	-4.2560252	-3.2613850	0.3885760
H	-4.9340949	-4.0737857	0.1145431
C	-3.7454980	-3.1813621	1.7081800
H	-4.0231848	-3.9405665	2.4439817
C	-2.8956519	-2.1564088	2.0717033
H	-2.5047412	-2.1119652	3.0887127
C	-2.5011308	-1.1565155	1.1359870
C	-1.5972534	-0.0893489	1.4816114
C	-1.1385915	0.1608094	2.8778186
C	0.2235879	0.2117179	3.1598982
C	0.7147319	0.4000933	4.4760701
H	1.7966724	0.3968920	4.6216343
C	-0.1628854	0.5701017	5.5191369
H	0.2102155	0.7044190	6.5382007
C	-1.5671611	0.5992862	5.2885761
C	-2.4840723	0.8314649	6.3517698
H	-2.0878045	0.9584266	7.3633195
C	-3.8406890	0.9082704	6.1183760
H	-4.5338212	1.0902749	6.9437471
C	-4.3333586	0.7667277	4.7979581
H	-5.4065576	0.8528339	4.6076600
C	-3.4716109	0.5279325	3.7465826
H	-3.8712411	0.4327666	2.7368144
C	-2.0656470	0.4144698	3.9526677
Br	4.6735719	-0.3750850	-0.3948543
N	-0.5852318	-0.9087846	-4.0725906
H	-0.8355029	-1.8539789	-4.3398363
N	2.1208954	2.4759181	1.4850677
O	0.7938060	0.2687549	-2.6809067
O	-0.1456136	1.6572342	0.7050615
O	1.1543028	0.0341836	2.1685609
P	1.4358523	1.0893836	0.8816299
Pd	2.1883134	-0.3573481	-0.7816971

S-7b-INT1

C	5.0850517	1.4699106	1.9390891
H	5.7269130	1.6603877	2.8005097
C	3.7473628	0.9985184	1.8093571
H	2.9499249	0.7593323	2.5139295
C	3.8703329	0.9607917	0.3871344
H	3.2269556	0.5542609	-0.3946902
C	5.4206267	0.9480863	0.5089978
H	5.8025516	-0.0898118	0.5520868
C	6.2671112	1.7950112	-0.4377635
C	8.4184964	1.6531815	-1.7365068
C	9.4658350	0.7745170	-2.0634812
H	9.4582151	-0.2482777	-1.6727544
C	10.5132555	1.1896364	-2.8842000
H	11.3145067	0.4833479	-3.1208480
C	10.5573106	2.4917528	-3.4068115
C	9.5011030	3.3546704	-3.0754804
H	9.5016119	4.3756716	-3.4691884
C	8.4421276	2.9590680	-2.2557025
H	7.6355359	3.6467215	-2.0129167
C	11.7073385	2.9540831	-4.2686341
H	12.1891567	2.1112702	-4.7881222
H	12.4856467	3.4521420	-3.6629277
H	11.3776168	3.6796368	-5.0288074
C	4.1130918	5.9363851	-1.1265233
H	4.0995199	7.0179160	-0.9019638
H	4.6156998	5.7859150	-2.0983353
H	4.6988129	5.4362847	-0.3453270
C	1.8929796	6.0478024	-2.1737912
H	0.9119235	5.5619879	-2.2169992
H	2.3597704	5.9713907	-3.1722715
H	1.7507137	7.1190705	-1.9430154
C	0.1113107	2.7209482	-0.5585387
C	0.1963750	1.3202239	-0.7559156
H	1.0358551	0.9352264	-1.3376512
C	-0.7723799	0.4907100	-0.2437785
H	-0.7201075	-0.5889767	-0.4087572
C	-1.8419993	1.0204635	0.5300150
C	-2.8068464	0.1672219	1.1360219
H	-2.7294602	-0.9106063	0.9662773
C	-3.8090505	0.6792124	1.9316296
H	-4.5387479	0.0126127	2.3986113
C	-3.8784734	2.0753413	2.1597042
H	-4.6558960	2.4794393	2.8135610
C	-2.9697395	2.9312857	1.5713778
H	-3.0337579	4.0012617	1.7697001
C	-1.9337500	2.4424615	0.7228862
C	-0.9640143	3.3114675	0.1009389
C	-1.0605773	4.7986066	0.1771775
C	0.0192762	5.5255212	0.6688953
C	-0.0342953	6.9279574	0.8647082
H	0.8382978	7.4185799	1.3005505
C	-1.1686621	7.6220080	0.5187818
H	-1.2250271	8.7022869	0.6786490
C	-2.2756045	6.9544658	-0.0774411
C	-3.4258351	7.6747898	-0.5060310
H	-3.4557678	8.7552857	-0.3384837
C	-4.4749687	7.0353610	-1.1309574
H	-5.3502872	7.6016566	-1.4596061
C	-4.4073675	5.6397396	-1.3614905
H	-5.2268553	5.1361610	-1.8813553
C	-3.3140583	4.9087697	-0.9421409
H	-3.2782936	3.8366867	-1.1375488
C	-2.2209113	5.5300747	-0.2702648
Br	4.5368278	4.9649927	2.5200389
N	7.3947999	1.1658404	-0.8948622
H	7.5298993	0.2146713	-0.5724925
N	2.7506381	5.4081015	-1.1805429
O	5.9510810	2.9393511	-0.7333904
O	1.1225913	3.4918593	-1.0919729
O	1.1817520	4.8830541	1.0232522
P	2.2515045	4.2455422	-0.0963307
Pd	3.8565581	3.0242329	1.0696342

7b-INT1

C	4.7388692	-0.4175018	0.5847227
H	5.4412935	-0.6393949	1.3904262
C	3.3368069	-0.6065863	0.4555308
H	2.5474254	-0.9336329	1.1347353
C	3.3599568	-0.1607093	-0.8988345
H	2.6197043	-0.1472909	-1.7006398
C	4.8937042	-0.3849217	-0.9614080
H	5.1258078	-1.3847166	-1.3727740
C	5.7354270	0.6074338	-1.7703358
C	7.5221635	2.3402149	-1.5536680
C	8.1114145	3.2022168	-0.6100651
H	7.8608596	3.0917842	0.4480755
C	8.9799559	4.2088948	-1.0228017
H	9.4232624	4.8676091	-0.2696579
C	9.2905348	4.3987100	-2.3803424
C	8.6929361	3.5330296	-3.3063660
H	8.9145412	3.6517070	-4.3713951
C	7.8182504	2.5144603	-2.9153008
H	7.3586741	1.8624935	-3.6543760
C	10.2214629	5.5063663	-2.8109782
H	11.1629359	5.4856944	-2.2371279
H	9.7662845	6.4985419	-2.6439650
H	10.4750469	5.4329517	-3.8796596
C	4.6097927	4.1714570	-1.8991541
H	4.9558898	5.2185237	-1.9342560
H	4.9115718	3.6635279	-2.8305370
H	5.1211230	3.6870746	-1.0575147
C	2.3850813	4.9589940	-2.6398151
H	1.3090517	4.8228427	-2.4781160
H	2.6176370	4.6786478	-3.6822461
H	2.6298275	6.0269927	-2.5012339
C	0.0209073	2.3430798	-0.4363999

C	-0.2508371	0.9544457	-0.4248967
H	0.3539087	0.3041299	-1.0582790
C	-1.2666711	0.4633505	0.3613101
H	-1.4935665	-0.6062669	0.3653034
C	-2.0194647	1.3323347	1.1991664
C	-3.0310144	0.8290638	2.0648965
H	-3.2431038	-0.2438401	2.0521006
C	-3.7212348	1.6675466	2.9134863
H	-4.4918812	1.2678027	3.5776131
C	-3.4163834	3.0511074	2.9368928
H	-3.9460318	3.7105268	3.6294885
C	-2.4521214	3.5734512	2.0997735
H	-2.2234294	4.6385801	2.1391423
C	-1.7355431	2.7419643	1.1906703
C	-0.7219330	3.2545849	0.3059459
C	-0.4064794	4.7078891	0.2009124
C	0.8975377	5.1335235	0.4318207
C	1.2661151	6.4997781	0.4168229
H	2.3043557	6.7536057	0.6385224
C	0.3198073	7.4563164	0.1348318
H	0.5899954	8.5157333	0.1327544
C	-1.0171549	7.0848735	-0.1826054
C	-1.9909618	8.0616896	-0.5342561
H	-1.6985803	9.1156459	-0.5288486
C	-3.2721534	7.6944073	-0.8865916
H	-4.0091063	8.4546486	-1.1580067
C	-3.6285425	6.3233375	-0.9117279
H	-4.6375379	6.0311999	-1.2144787
C	-2.7124389	5.3518688	-0.5637119
H	-3.0011139	4.3010700	-0.5970674
C	-1.3867129	5.6952540	-0.1692861
Br	5.2296808	2.8830842	1.9106137
N	6.6559244	1.3357234	-1.0679129
H	6.5790159	1.3044679	-0.0535829
N	3.1572543	4.1289643	-1.7197243
O	5.5562520	0.6762023	-2.9761984
O	1.0623416	2.7844448	-1.2328713
O	1.8873988	4.2112664	0.6988875
P	2.5013593	3.2215113	-0.4972095
Pd	3.7659346	1.5406990	0.3786906

S-7b-TS1
(-88.99 cm^{-1})

C	-0.5258784	-1.5542519	2.2973709
H	0.2429177	-0.8259071	2.5719425
C	-0.7139930	-2.9135830	2.8047352
H	-0.0225716	-3.6880385	3.1432481
C	-2.0804884	-2.8638307	2.6711194
H	-2.8884094	-3.5884806	2.7894255
C	-2.0823337	-1.3449120	2.4530233
H	-2.3327275	-0.8069229	3.3855715
C	-2.8460069	-0.7946489	1.2598283
C	-4.5386713	0.9567166	0.6449211
C	-5.2157043	2.0760255	1.1568143
H	-5.0756630	2.3638115	2.2037457
C	-6.0579504	2.8322346	0.3419554
H	-6.5686121	3.7010883	0.7675992
C	-6.2575769	2.5006064	-1.0069955
C	-5.5748567	1.3777285	-1.5023367
H	-5.7068771	1.0899616	-2.5499129
C	-4.7271386	0.6071428	-0.7040954
H	-4.2069561	-0.2560878	-1.1132722
C	-7.1886126	3.2999280	-1.8864974
H	-7.3833443	4.2994969	-1.4683505
H	-8.1645472	2.7949509	-1.9987130
H	-6.7759341	3.4296822	-2.8999167
C	2.7300918	-4.0300632	-0.6008773
H	3.6796323	-4.2508225	-0.0785885
H	2.6749631	-4.6432898	-1.5155190
H	1.8869120	-4.3239839	0.0372686
C	3.6786939	-2.1450610	-1.8625503
H	3.5359254	-1.0867110	-2.1068394
H	3.6289702	-2.7255996	-2.8007648
H	4.6850985	-2.2764888	-1.4257437
C	0.7679810	0.7166946	-1.1671141
C	-0.5545946	0.9289224	-1.6216746
H	-1.0479540	0.1282566	-2.1748962
C	-1.1819516	2.1205886	-1.3457674
H	-2.2029431	2.2972118	-1.6935422
C	-0.5345593	3.1201325	-0.5668485
C	-1.2054464	4.3253201	-0.2136731
H	-2.2216104	4.4830278	-0.5861363
C	-0.6017578	5.2660775	0.5929264
H	-1.1301195	6.1851208	0.8601955
C	0.7035823	5.0306666	1.0899851
H	1.1731558	5.7650180	1.7500469
C	1.3872941	3.8798845	0.7538124
H	2.3870550	3.7126817	1.1556048
C	0.8061397	2.8951716	-0.0978649
C	1.4862757	1.6767054	-0.4608067
C	2.8952598	1.3870872	-0.0634245
C	3.1756617	0.2188020	0.6418110
C	4.4754681	-0.0836311	1.1178432
H	4.6132775	-1.0011901	1.6933678
C	5.5180290	0.7735547	0.8584514
H	6.5209979	0.5527922	1.2338970
C	5.3171646	1.9434557	0.0740529
C	6.4021947	2.8023491	-0.2597310
H	7.3961759	2.5614273	0.1281792
C	6.2151372	3.9050101	-1.0652796
H	7.0577974	4.5536136	-1.3185446
C	4.9261077	4.1859670	-1.5807681
H	4.7823391	5.0447743	-2.2418730
C	3.8499531	3.3823660	-1.2632056
H	2.8681867	3.6089904	-1.6795758
C	3.9983508	2.2484740	-0.4119047
Br	-0.7976475	-4.1754822	-1.4097705
N	-3.7008516	0.2371339	1.5256078
H	-3.7207097	0.5547006	2.4878872
N	2.6476042	-2.6128088	-0.9394267

O	-2.6751590	-1.2793509	0.1461737
O	1.3578870	-0.4993852	-1.4644953
O	2.1703231	-0.6800751	0.9284417
P	1.4687173	-1.6517009	-0.2674783
Pd	-0.4521506	-2.5232475	0.4636191

7b-TS1

(-79.87 cm $^{-1}$)

C	-0.4443597	-1.5692685	2.3430605
H	0.3450604	-0.8490354	2.5783296
C	-0.6747011	-2.8943479	2.9222554
H	-0.0073708	-3.6712941	3.3022830
C	-2.0394256	-2.8092037	2.7816152
H	-2.8701711	-3.5022876	2.9241817
C	-1.9919641	-1.3044931	2.4886260
H	-2.2176077	-0.7147007	3.3957451
C	-2.7486654	-0.7870087	1.2764520
C	-4.2501204	1.0839893	0.5557222
C	-4.6121322	2.3968373	0.8972281
H	-4.2858598	2.8186088	1.8526547
C	-5.3561308	3.1808330	0.0155019
H	-5.6180384	4.2030685	0.3040164
C	-5.7614235	2.6864533	-1.2338192
C	-5.3971449	1.3688576	-1.5566289
H	-5.6971517	0.9525941	-2.5230296
C	-4.6584239	0.5660764	-0.6860630
H	-4.3725860	-0.4443243	-0.9697214
C	-6.5837520	3.5230733	-2.1831854
H	-6.4720073	4.5990909	-1.9781763
H	-7.6579218	3.2811458	-2.0956716
H	-6.2965818	3.3434769	-3.2314040
C	2.7695880	-4.0839875	-0.6908939
H	3.7356977	-4.2969043	-0.1966744
H	2.7153362	-4.6550584	-1.6324245
H	1.9476406	-4.4346350	-0.0541761
C	3.6545676	-2.1008731	-1.8534740
H	3.4834259	-1.0321604	-2.0255314
H	3.6193800	-2.6190587	-2.8280030
H	4.6637388	-2.2305853	-1.4231480
C	0.5986843	0.5818417	-1.0375817
C	-0.7552936	0.7286108	-1.4158308
H	-1.2373388	-0.0906636	-1.9507636
C	-1.4255110	1.8829134	-1.0858782
H	-2.4652930	2.0182954	-1.3874329
C	-0.7939534	2.8933023	-0.3086887
C	-1.5075875	4.0532611	0.1068960
H	-2.5453476	4.1654032	-0.2167315
C	-0.9140404	5.0004264	0.9143070
H	-1.4737998	5.8848265	1.2303752
C	0.4237147	4.8197663	1.3463974
H	0.8858841	5.5607558	2.0041829
C	1.1481149	3.7150747	0.9471632
H	2.1748233	3.5887254	1.2927373
C	0.5754003	2.7258573	0.0957040
C	1.2983601	1.5594356	-0.3378465
C	2.7420570	1.3409167	-0.0369174
C	3.1323636	0.1723425	0.6115970
C	4.4789930	-0.0849003	0.9645286
H	4.7032520	-1.0148775	1.4903904
C	5.4563802	0.8277574	0.6457804
H	6.4972741	0.6422288	0.9244012
C	5.1354174	2.0123060	-0.0746611
C	6.1460751	2.9370522	-0.4619563
H	7.1797279	2.7347915	-0.1666333
C	5.8384833	4.0574431	-1.2039142
H	6.6247152	4.7575481	-1.4979519
C	4.4984745	4.2912203	-1.6006044
H	4.2586907	5.1660746	-2.2106551
C	3.4930972	3.4214387	-1.2308303
H	2.4696772	3.6117816	-1.5542467
C	3.7683450	2.2673731	-0.4408209
Br	-0.7699725	-4.3638744	-1.2264082
N	-3.4772325	0.3514355	1.4809821
H	-3.3892886	0.7733345	2.3981926
N	2.6391389	-2.6564444	-0.9626012
O	-2.6837532	-1.3790980	0.2058187
O	1.2428514	-0.5892424	-1.3968312
O	2.1946550	-0.7803256	0.9470756
P	1.4619831	-1.7511687	-0.2262570
Pd	-0.4001060	-2.6420102	0.5655664

5a

C	7.5348551	8.0404911	2.1601765
H	7.3734009	9.0219426	1.7031244
C	7.2328309	7.5001588	3.3638251
H	6.6847991	7.8689741	4.2366459
C	7.9626199	6.2093453	3.1009520
H	8.8605984	6.0154960	3.7153921
C	8.2837531	6.8199931	1.6695362
C	9.7697446	7.0266893	1.3671145
H	7.7993881	6.2639918	0.8464375
C	8.3396813	5.4006162	6.3779376
H	8.4211833	4.8425843	7.3313798
H	9.0467513	6.2499209	6.3941516
H	8.6176249	4.7194556	5.5569100
C	6.4382696	6.7386914	7.2414148
H	5.4074659	7.0346522	7.0025394
H	7.0622022	7.6477529	7.3240272
H	6.4439226	6.2100414	8.2141220
C	3.6056280	5.5836903	4.3713854
C	3.4174229	5.8230216	2.9887777
H	4.1463690	6.4465435	2.4626756
C	2.3276879	5.2581060	2.3538590
H	2.1600411	5.4331843	1.2854709
C	1.4138866	4.4302557	3.0709402

C	0.3017920	3.8193222	2.4187335
H	0.1464046	4.0195045	1.3524001
C	-0.5658360	2.9892540	3.1103455
H	-1.4172381	2.5306520	2.5965080
C	-0.3370602	2.7169725	4.4841418
H	-1.0021901	2.0333446	5.0217925
C	0.7365016	3.2901120	5.1467217
H	0.9181508	3.0485655	6.1966618
C	1.6259190	4.1820959	4.4772978
C	2.7472973	4.7996701	5.1383586
C	3.0854127	4.5562740	6.5680885
C	4.3452557	4.0380700	6.8856922
C	4.7211063	3.7107971	8.2114265
H	5.7099055	3.2765566	8.3802425
C	3.8318933	3.9244578	9.2454955
H	4.1076948	3.6525381	10.2701398
C	2.5571299	4.5143409	9.0021533
C	1.6504353	4.7948250	10.0663095
H	1.9411229	4.5204978	11.0868680
C	0.4347327	5.4118377	9.8249586
H	-0.2503707	5.6244088	10.6524478
C	0.0830861	5.7858648	8.5014379
H	-0.8674409	6.2974197	8.3162618
C	0.9348523	5.5165484	7.4425196
H	0.6571032	5.8176421	6.4284895
C	2.1831916	4.8564528	7.6503908
Cl	8.0896487	3.5780335	1.2563809
N	6.9730442	5.8877437	6.1716260
O	4.7349181	6.1392859	4.9882548
O	5.2726808	3.7922875	5.8815256
P	6.0153359	5.0653712	5.0721950
Pd	6.8434104	4.4093963	3.1100470
O	10.2946310	8.1404891	1.3852675
N	10.4198634	5.8410894	1.1097625
C	4.8692483	3.0457981	0.2575579
H	3.8025694	3.1192513	-0.0324994
H	5.4792372	2.8211362	-0.6358565
H	5.1970878	4.0190965	0.6572100
C	4.6705985	0.6481236	0.8918295
H	4.8768947	-0.0448627	1.7200541
H	5.2589511	0.3292000	0.0107910
H	3.5934319	0.5987359	0.6409652
C	6.6356224	1.0259961	4.6317124
C	7.9126925	1.4827276	5.0416507
H	8.6196802	1.8063355	4.2717841
C	8.2167518	1.5093495	6.3892150
H	9.2011353	1.8519730	6.7267087
C	7.2526887	1.1107050	7.3621587
C	7.5378341	1.1787748	8.7584190
H	8.5325101	1.5147474	9.0731174
C	6.5833179	0.8337918	9.7019686
H	6.8184433	0.8900764	10.7701619
C	5.2919975	0.4217472	9.2813238
H	4.5282275	0.1794942	10.0277182
C	4.9826689	0.3420552	7.9329812
H	3.9773126	0.0471546	7.6220331
C	5.9505877	0.6569043	6.9329268
C	5.6580188	0.5887042	5.5240944
C	4.3396097	0.1590166	4.9819700
C	3.6355228	1.0294308	4.1416454
C	2.3534532	0.7120610	3.6260438
H	1.8523694	1.4522141	2.9963337
C	1.7679118	-0.4975386	3.9402313
H	0.7689260	-0.7401168	3.5611691
C	2.4573196	-1.4561232	4.7397333
C	1.8905404	-2.7329173	5.0253414
H	0.8889690	-2.9582774	4.6412620
C	2.5889628	-3.6771509	5.7586333
H	2.1428503	-4.6553804	5.9669753
C	3.8963780	-3.3815839	6.2254258
H	4.4586834	-4.1389072	6.7821665
C	4.4721014	-2.1462052	5.9760083
H	5.4847980	-1.9354218	6.3312472
C	3.7700250	-1.1376687	5.2494487
N	5.0556783	2.0089973	1.2721128
O	6.3386393	1.0478219	3.2677800
O	4.1590245	2.2659343	3.8112792
P	5.5154628	2.4412389	2.8121559
H	9.8129756	5.0075867	1.1334755
H	13.8061453	2.9181272	0.1991459
C	13.5180437	3.9657685	0.3486645
C	12.1826077	4.2678291	0.6332283
H	11.4394684	3.4650009	0.7045144
C	11.7769032	5.6055662	0.8286774
C	12.7425591	6.6323584	0.7331238
H	12.4316681	7.6671025	0.8833905
C	14.0724171	6.3048747	0.4476833
H	14.8071470	7.1165653	0.3752682
C	14.4940557	4.9749128	0.2489996
C	15.9405045	4.6520224	-0.0577685
H	16.0870932	3.5696726	-0.2136811
H	16.6073734	4.9670916	0.7669084
H	16.2857568	5.1746135	-0.9694232

5a′

C	7.9754996	7.8670939	3.5488480
H	8.2931950	8.9023168	3.3849973
C	7.2902853	7.2377432	4.5324772
H	6.8018467	7.5792522	5.4505713
C	7.4887188	5.8673363	3.9483950
H	8.1675417	5.2172314	4.5422212
C	8.2329232	6.5998671	2.7583231
C	9.6805400	6.1307874	2.6398054
H	7.6892007	6.5139972	1.8010056
C	6.8347148	4.1298877	7.1799139
H	6.5246945	3.3431153	7.8928858
H	7.6904717	4.6872007	7.6019233
H	7.1515580	3.6399305	6.2446186
C	5.1536811	5.7542964	8.0557718
H	4.3437248	6.4189025	7.7225957

H	5.9376117	6.3645998	8.5408148
H	4.7517725	5.0360503	8.7954027
C	3.4915607	6.7367139	4.2642869
C	4.0064285	7.2913875	3.0674283
H	5.0617621	7.5771772	3.0336558
C	3.1642112	7.4405561	1.9818028
H	3.5447974	7.8657612	1.0472511
C	1.8061445	7.0094362	2.0437884
C	0.9488425	7.0958390	0.9069450
H	1.3453854	7.5394627	-0.0130920
C	-0.3495397	6.6172380	0.9527755
H	-0.9942611	6.6861236	0.0703435
C	-0.8407850	6.0194224	2.1429747
H	-1.8599236	5.6192882	2.1696228
C	-0.0398070	5.9314226	3.2705897
H	-0.4265105	5.4592776	4.1778569
C	1.2948012	6.4376202	3.2670189
C	2.1626514	6.3403356	4.4133575
C	1.7363240	5.7340495	5.7040318
C	2.4533252	4.6372701	6.1875162
C	2.0906325	3.9476250	7.3714853
H	2.6891889	3.0826374	7.6722461
C	1.0007236	4.3798844	8.1018144
H	0.7016971	3.8504490	9.0133288
C	0.2618429	5.5311656	7.6984633
C	-0.8327260	6.0193812	8.4706579
H	-1.1174313	5.4747554	9.3781475
C	-1.5177375	7.1619226	8.0945349
H	-2.3543213	7.5276699	8.6991941
C	-1.1244097	7.8709099	6.9295912
H	-1.6539410	8.7870952	6.6471091
C	-0.0721233	7.4189849	6.1502597
H	0.2281546	7.9780034	5.2591479
C	0.6405111	6.2316783	6.4943560
Cl	6.7633209	3.9873783	1.3380244
N	5.7267367	5.0526709	6.9017245
O	4.3759976	6.5475702	5.3357543
O	3.5380957	4.1565154	5.4706248
P	5.0075329	4.9962351	5.4037576
Pd	6.0946207	4.3217536	3.5993351
O	10.6102766	6.6704736	3.2377542
N	9.7864608	4.9879393	1.8717390
C	2.8010458	2.9591226	2.4107744
H	1.8744074	2.9932595	3.0142853
H	2.5359733	2.8547443	1.3430316
H	3.3396677	3.9118717	2.5449619
C	3.1378616	0.4911132	2.6380232
H	3.8970707	-0.2451508	2.9402813
H	2.8926929	0.3272629	1.5719495
H	2.2235856	0.3371743	3.2426576
C	7.2626482	0.8026133	3.7444153
C	8.4198009	1.3553090	3.1439867
H	8.3189279	1.8508790	2.1761359
C	9.6309178	1.2712496	3.8038043
H	10.5332036	1.6917927	3.3479673
C	9.7156854	0.6709857	5.0939782
C	10.9477211	0.6424476	5.8120064
H	11.8366893	1.0684071	5.3331085
C	11.0179559	0.1066089	7.0868645
H	11.9691751	0.0950066	7.6295304
C	9.8477425	-0.4144280	7.6990351
H	9.9001738	-0.8166301	8.7165772
C	8.6367810	-0.4133491	7.0246069
H	7.7415182	-0.8094092	7.5122769
C	8.5321291	0.1085261	5.7001820
C	7.2866537	0.1413717	4.9742262
C	6.0193757	-0.4274221	5.5091379
C	4.9076689	0.4111232	5.6258860
C	3.6741236	-0.0288329	6.1711804
H	2.8543267	0.6920306	6.2460752
C	3.5414179	-1.3390694	6.5888742
H	2.5973915	-1.6891466	7.0209711
C	4.6178728	-2.2635442	6.4416630
C	4.4785481	-3.6320099	6.8180943
H	3.5282099	-3.9606199	7.2541556
C	5.5121209	-4.5335471	6.6284470
H	5.3899566	-5.5823323	6.9192556
C	6.7287018	-4.0994502	6.0394455
H	7.5360072	-4.8193788	5.8678195
C	6.9019062	-2.7746661	5.6724913
H	7.8396610	-2.4531672	5.2112118
C	5.8681281	-1.8122386	5.8774781
N	3.6633683	1.8458872	2.8217672
O	6.0429679	0.9357942	3.0666196
O	5.0034569	1.7372887	5.2413937
P	5.0848805	2.2025863	3.6039583
H	8.8943667	4.6602262	1.4712176
H	13.9255732	3.2758933	3.0332525
C	13.0178806	3.1873735	2.4237303
C	12.0386379	4.1846673	2.5321272
H	12.1683548	5.0364574	3.2015479
C	10.8572195	4.0861525	1.7671895
C	10.7102489	2.9957004	0.8789504
H	9.7998855	2.9177838	0.2739390
C	11.6998844	2.0150589	0.7867405
H	11.5515862	1.1736230	0.0992121
C	12.8748853	2.0820600	1.5642283
C	13.9340899	1.0062273	1.4662532
H	14.7762801	1.2073220	2.1496725
H	14.3410331	0.9341342	0.4403755
H	13.5214208	0.0121628	1.7207484

S-5a′

C	7.9000762	7.5951465	2.9837485
H	8.0891219	8.6190766	2.6455098
C	7.3390477	7.0570786	4.0913725
H	6.8575345	7.4895581	4.9736227
C	7.6579242	5.6414741	3.7106706
H	8.3891022	5.1240080	4.3672448
C	8.2930147	6.2551994	2.3932846

C	9.8045374	6.0590766	2.2307592
H	7.7539370	5.9487192	1.4785861
C	6.8761128	4.2820809	7.1467842
H	6.5667132	3.5893912	7.9519050
H	7.7279852	4.8899533	7.5031778
H	7.2016383	3.6849216	6.2786213
C	5.2460161	6.0352524	7.8063508
H	4.4491404	6.6752469	7.4037765
H	6.0625015	6.6793477	8.1829696
H	4.8459541	5.4420711	8.6500709
C	3.4739000	6.7126753	4.0937818
C	3.8590363	7.3161947	2.8712014
H	4.9120792	7.5740568	2.7254484
C	2.9000928	7.5632654	1.9081846
H	3.1807391	8.0391327	0.9624362
C	1.5428486	7.1773064	2.1119574
C	0.5594045	7.3704882	1.0972336
H	0.8622183	7.8592442	0.1644046
C	-0.7438240	6.9384371	1.2732617
H	-1.4873705	7.0893311	0.4834938
C	-1.1109701	6.2800591	2.4756506
H	-2.1351693	5.9130750	2.6029162
C	-0.1842936	6.0907015	3.4888933
H	-0.4811168	5.5727768	4.4046400
C	1.1613831	6.5487868	3.3549516
C	2.1540221	6.3633759	4.3873262
C	1.8422164	5.7545499	5.7103355
C	2.5744045	4.6388577	6.1264455
C	2.2892664	3.9421294	7.3287267
H	2.8834100	3.0558191	7.5694294
C	1.2752107	4.3904951	8.1522467
H	1.0365217	3.8531168	9.0769040
C	0.5439578	5.5717004	7.8314474
C	-0.4554908	6.0849035	8.7095123
H	-0.6758517	5.5316487	9.6295951
C	-1.1237547	7.2619615	8.4201777
H	-1.8848973	7.6480601	9.1064154
C	-0.8066979	7.9797662	7.2379050
H	-1.3166651	8.9251337	7.0236944
C	0.1471179	7.5003233	6.3542628
H	0.3870689	8.0697653	5.4520008
C	0.8386159	6.2772150	6.6066118
Cl	7.2976715	3.2240051	1.6105113
N	5.7677553	5.1587395	6.7509313
O	4.4676005	6.5058372	5.0533482
O	3.5996886	4.1456286	5.3276976
P	5.0844139	4.9566981	5.2405232
Pd	6.3030077	4.0077339	3.6240402
O	10.6063970	6.9522754	2.5079763
N	10.1466693	4.8041061	1.7760137
C	3.2650647	2.6151767	2.0686231
H	2.2123233	2.6562258	2.4065697
H	3.2881965	2.5018668	0.9691065
H	3.7577087	3.5654473	2.3321813
C	3.4958724	0.1584793	2.3712976
H	4.1335897	-0.5975143	2.8497775
H	3.5341293	0.0085861	1.2756943
H	2.4515428	0.0227515	2.7111849
C	7.2523757	0.2100103	4.2516530
C	8.5631346	0.5669425	3.8476472
H	8.6869011	1.0702265	2.8852924
C	9.6302635	0.2794602	4.6761180
H	10.6486823	0.5430205	4.3702268
C	9.4227692	-0.3286965	5.9491921
C	10.5102082	-0.5659470	6.8409968
H	11.5212405	-0.3004715	6.5117811
C	10.2993237	-1.1032469	8.0992639
H	11.1436107	-1.2756514	8.7753093
C	8.9801136	-1.4143248	8.5195719
H	8.8108241	-1.8143486	9.5253058
C	7.9021337	-1.2108123	7.6723236
H	6.8910914	-1.4460463	8.0155617
C	8.0835578	-0.6841522	6.3575980
C	6.9825279	-0.4526321	5.4518572
C	5.5780832	-0.8388520	5.7643061
C	4.5763914	0.1354154	5.6985311
C	3.2269534	-0.1365778	6.0451923
H	2.5030598	0.6821538	5.9951434
C	2.8626616	-1.4091834	6.4387501
H	1.8260495	-1.6252933	6.7211568
C	3.8179650	-2.4675704	6.4548413
C	3.4403328	-3.8002528	6.7939972
H	2.3989614	-3.9913736	7.0777749
C	4.3583875	-4.8355266	6.7520731
H	4.0535264	-5.8554522	7.0095177
C	5.6954025	-4.5744100	6.3546862
H	6.4146252	-5.3983606	6.2940343
C	6.0995203	-3.2879175	6.0345032
H	7.1303948	-3.1052966	5.7194301
C	5.1882692	-2.1901590	6.0933121
N	3.9780962	1.5025086	2.7020130
O	6.2053065	0.5045889	3.3791754
O	4.8896847	1.4356709	5.3306962
P	5.2541923	1.8456835	3.7184783
H	9.3465936	4.1767238	1.6045802
H	12.7673587	1.2600887	0.5690565
C	12.7396797	2.2994108	0.9182576
C	11.4991663	2.9117330	1.1276939
H	10.5741053	2.3542455	0.9402026
C	11.4238813	4.2490368	1.5753312
C	12.6257185	4.9568658	1.8034121
H	12.5714945	5.9906318	2.1473506
C	13.8543574	4.3229969	1.5869621
H	14.7744468	4.8913949	1.7715002
C	13.9465713	2.9886435	1.1422225
C	15.2895254	2.3333752	0.9019516
H	15.1774606	1.2598371	0.6737933
H	15.9491973	2.4266071	1.7841402
H	15.8204454	2.8026135	0.0518701

5b

C	8.7228984	8.2921481	2.8077956
H	9.0729309	9.0617601	2.0920658
C	8.9626457	8.5898622	4.2571269
H	8.5982648	9.3943935	4.9051771
C	9.8093193	7.5485675	4.4409734
H	10.4375771	7.2027252	5.2675531
C	9.6973998	7.0614072	3.0069995
C	11.0457058	7.0176062	2.2702812
H	9.1924884	6.0842216	2.8705329
C	6.9435897	6.0495798	4.9757812
H	6.2348036	5.2731327	5.3240971
H	7.9078054	5.9329734	5.5019312
H	7.1119469	5.9071437	3.8947479
C	6.1414017	7.7432329	6.6042619
H	5.7192018	8.7561495	6.6594927
H	7.0914563	7.7203090	7.1704316
H	5.4300565	7.0320688	7.0657870
C	5.0479100	10.6858315	3.7954361
C	5.7227791	11.4963836	2.8502660
H	6.8102770	11.4123790	2.7693049
C	4.9892563	12.3574151	2.0563808
H	5.4937217	12.9926869	1.3206117
C	3.5689590	12.4211745	2.1690568
C	2.7981536	13.2809654	1.3315489
H	3.3242321	13.9175640	0.6111487
C	1.4158093	13.3143809	1.4198948
H	0.8379071	13.9817550	0.7718946
C	0.7466909	12.4661885	2.3403551
H	-0.3473650	12.4680883	2.3855855
C	1.4650875	11.6169754	3.1667951
H	0.9358932	10.9497589	3.8511951
C	2.8907589	11.5832752	3.1295713
C	3.6666115	10.7113241	3.9740763
C	3.0544170	9.7632648	4.9444671
C	3.3262777	8.3980154	4.8100903
C	2.7366186	7.4201239	5.6481293
H	2.9720361	6.3682463	5.4673709
C	1.8740837	7.8123088	6.6522775
H	1.3984421	7.0597481	7.2905417
C	1.6046398	9.1925556	6.8867427
C	0.7618360	9.6166901	7.9558603
H	0.2937665	8.8526902	8.5868926
C	0.5457047	10.9614841	8.2047620
H	-0.1011745	11.2724944	9.0318519
C	1.1791289	11.9405299	7.3949079
H	1.0271693	13.0037706	7.6093243
C	1.9937875	11.5631609	6.3396823
H	2.4834773	12.3261506	5.7278542
C	2.2172410	10.1861164	6.0372841
Cl	7.9297512	7.5929923	-0.1316520
N	6.4071773	7.3881884	5.2081501
O	5.8091247	9.7886025	4.5579829
O	4.1777969	7.9538682	3.8088576
P	5.8315127	8.2471533	3.8985572
Pd	6.8074388	8.1043316	1.9033167
O	12.0946922	6.8721882	2.9003155
N	10.9470159	7.1292848	0.9069095
C	5.9179281	10.1523378	-1.0965620
H	5.3849341	11.1180374	-1.2049515
H	6.5495364	9.9793258	-1.9858373
H	6.5778208	10.2066881	-0.2159357
C	4.0190131	8.8498871	-2.0314627
H	3.3637855	7.9966524	-1.8074591
H	4.5769100	8.6331623	-2.9616734
H	3.3951418	9.7509343	-2.1885469
C	3.7609755	6.0461382	1.1197439
C	4.6403141	5.0826630	1.6716390
H	5.6322646	4.9686961	1.2244909
C	4.2208879	4.3289138	2.7504720
H	4.8826048	3.5729244	3.1875019
C	2.9309999	4.5329670	3.3235608
C	2.5047699	3.7936281	4.4665768
H	3.1787452	3.0340978	4.8793040
C	1.2704857	4.0275413	5.0509248
H	0.9573376	3.4506504	5.9276954
C	0.4172378	5.0291809	4.5197748
H	-0.5446634	5.2354240	5.0008140
C	0.7984662	5.7625168	3.4078823
H	0.1417721	6.5471247	3.0232971
C	2.0478033	5.5270246	2.7603004
C	2.4759772	6.2772966	1.6074712
C	1.6522340	7.3437280	0.9736036
C	2.1834768	8.6338064	0.8588358
C	1.4389735	9.7145235	0.3220828
H	1.9102094	10.7005876	0.2908311
C	0.1499883	9.5046470	-0.1240306
H	-0.4372270	10.3398711	-0.5212530
C	-0.4282386	8.2014702	-0.0972081
C	-1.7365970	7.9570178	-0.6080498
H	-2.3112124	8.8039851	-1.0000967
C	-2.2701584	6.6795008	-0.6251340
H	-3.2751244	6.5059949	-1.0243265
C	-1.5025623	5.5881740	-0.1409320
H	-1.9146268	4.5741425	-0.1809417
C	-0.2324231	5.7924126	0.3737230
H	0.3531161	4.9417075	0.7331582
C	0.3377045	7.0997325	0.4353451
N	4.9661305	9.0534711	-0.9326367
O	4.2247262	6.8182467	0.0533372
O	3.4600371	8.9116504	1.3101973
P	4.8427151	8.2995407	0.5473434
H	9.9901928	7.2509019	0.5387462
H	13.6311192	6.8171932	1.2932528
C	13.3354499	6.9244145	0.2490777
C	11.9679159	7.0931860	-0.0629017
C	11.5894095	7.2327087	-1.4155574
H	10.5308823	7.3642378	-1.6658575
C	12.5536196	7.2028785	-2.4282581
H	12.2327403	7.3124738	-3.4712096
C	13.9202667	7.0344744	-2.1372372

C	14.9666492	7.0003117	-3.2299879
C	14.2808676	6.8976106	-0.7819417
H	15.3377047	6.7642689	-0.5193780
H	14.5114178	7.1184405	-4.2279729
H	15.5240623	6.0448729	-3.2239495
H	15.7105475	7.8086085	-3.1001636

5b′

C	7.5693106	7.9298535	2.6604690
H	8.1407932	8.8416792	2.4031177
C	7.5943142	7.4546260	4.0884222
H	7.2620253	7.9263855	5.0189749
C	8.1829226	6.2704995	3.8016797
H	8.5417974	5.4282881	4.4009744
C	8.2758333	6.5510156	2.3142262
C	9.7119722	6.6330825	1.7791961
H	7.6653699	5.8992310	1.6600491
C	4.0911707	6.2583215	4.1441847
H	3.0301269	5.9596530	4.0767925
H	4.6233000	5.5763245	4.8311659
H	4.5461114	6.1658487	3.1437654
C	3.7433987	7.9050812	5.9769214
H	3.9242983	8.9562409	6.2422229
H	4.2882171	7.2589958	6.6906161
H	2.6605989	7.6943780	6.0571977
C	5.8331438	11.1062631	4.3550948
C	7.1660748	11.4390547	4.0128764
H	7.9196273	10.6475335	4.0130870
C	7.4784135	12.7474324	3.6992182
H	8.5076939	13.0208931	3.4432097
C	6.4680994	13.7532883	3.6759519
C	6.7678031	15.0954694	3.2988813
H	7.8049640	15.3499202	3.0533507
C	5.7735679	16.0561220	3.2287021
H	6.0174814	17.0822650	2.9340918
C	4.4302797	15.7045037	3.5226232
H	3.6410789	16.4597165	3.4412604
C	4.1060599	14.4127835	3.9044845
H	3.0653341	14.1536914	4.1176278
C	5.1092445	13.4035975	4.0151432
C	4.8068002	12.0508458	4.4113629
C	3.4415715	11.6165843	4.8181451
C	2.8442167	10.5525435	4.1393151
C	1.5269816	10.1065881	4.4137527
H	1.1292257	9.2761712	3.8247272
C	0.7988837	10.7221712	5.4135630
H	-0.2234591	10.3957794	5.6337084
C	1.3729727	11.7673239	6.1967607
C	0.6565280	12.3655280	7.2752330
H	-0.3665752	12.0272916	7.4755215
C	1.2393427	13.3418608	8.0652084
H	0.6792618	13.7888460	8.8932602
C	2.5743832	13.7521080	7.8115132
H	3.0424891	14.5055762	8.4538170
C	3.2944213	13.2024809	6.7631437
H	4.3250254	13.5195842	6.5824399
C	2.7171333	12.2124381	5.9128872
Cl	6.7921316	7.9292081	-0.4432815
N	4.2214576	7.6404605	4.6161249
O	5.5649408	9.7791850	4.6881837
O	3.5521016	9.8962922	3.1404687
P	4.7723459	8.8078264	3.5703637
Pd	5.7223959	8.1804269	1.6684548
O	10.6667113	6.2136883	2.4348282
N	9.7949045	7.2175333	0.5382917
C	3.7188906	10.3775848	-0.6602439
H	2.9713871	11.1580296	-0.4225773
H	4.1382583	10.5673158	-1.6652123
H	4.5386406	10.4339892	0.0749053
C	2.0274069	8.7658948	-1.5527638
H	1.7056149	7.7187324	-1.4522254
H	2.3794516	8.9261347	-2.5890315
H	1.1634443	9.4308159	-1.3621792
C	3.7741486	5.3973912	0.4289992
C	5.0786953	4.8425992	0.4492585
H	5.8506035	5.3133485	-0.1660845
C	5.3336392	3.7525435	1.2593742
H	6.3369870	3.3145570	1.2915672
C	4.3122215	3.2039607	2.0904585
C	4.5858975	2.1292564	2.9872465
H	5.5961968	1.7053328	3.0008140
C	3.6070416	1.6385719	3.8347950
H	3.8332783	0.8164271	4.5218345
C	2.3096752	2.2142953	3.8225990
H	1.5425651	1.8395033	4.5086156
C	2.0055740	3.2508351	2.9541845
H	1.0053375	3.6924218	2.9622134
C	2.9832492	3.7670927	2.0517460
C	2.7112904	4.8695054	1.1631472
C	1.3790446	5.5249237	1.0615027
C	1.2821688	6.8984458	1.3058329
C	0.0429140	7.5886550	1.2724047
H	0.0420710	8.6613313	1.4876040
C	-1.1172133	6.9009327	0.9738824
H	-2.0800455	7.4234380	0.9560086
C	-1.0796365	5.5104599	0.6585676
C	-2.2609563	4.7980468	0.2976565
H	-3.2155752	5.3364093	0.2936334
C	-2.2082286	3.4596342	-0.0528095
H	-3.1228543	2.9255877	-0.3309539
C	-0.9607226	2.7825676	-0.0681804
H	-0.9176734	1.7306309	-0.3697070
C	0.2049233	3.4419625	0.2871700
H	1.1609108	2.9120252	0.2615419
C	0.1846294	4.8139757	0.6809212
N	3.1139165	9.0436854	-0.6107623
O	3.5591274	6.5394292	-0.3450366
O	2.4079864	7.6302671	1.6410739
P	3.6589074	7.9710991	0.5327693
H	8.8906492	7.5047495	0.1325734

H	12.4056080	6.6685462	1.1082229
C	12.2479028	7.1644321	0.1496938
C	10.9310796	7.4911476	-0.2449351
C	10.7311460	8.1310603	-1.4870298
H	9.7133902	8.3882870	-1.8017261
C	11.8193076	8.4377152	-2.3103248
H	11.6361821	8.9357336	-3.2701506
C	13.1380341	8.1219661	-1.9334037
C	14.3186593	8.4532868	-2.8204862
C	13.3199062	7.4821358	-0.6911404
H	14.3347072	7.2207344	-0.3661630
H	13.9959428	8.9468272	-3.7529088
H	14.8825899	7.5430267	-3.0981092
H	15.0296832	9.1288744	-2.3087390

S-5b′

C	7.8387161	8.1901624	2.9057532
H	8.1442292	9.2152952	2.6216655
C	8.0352156	7.7871228	4.3408374
H	7.6070596	8.1685414	5.2738579
C	8.9519092	6.8321140	4.0624569
H	9.5727082	6.1685575	4.6727184
C	8.9018571	7.0600645	2.5653720
C	10.2248418	7.5323857	1.9495255
H	8.4731381	6.2353020	1.9668279
C	4.6133822	6.3247958	4.7573831
H	3.5818890	5.9332728	4.8311829
H	5.2660725	5.7674642	5.4541328
H	4.9776369	6.1649471	3.7282194
C	4.2990288	8.1125500	6.4513192
H	4.4099063	9.1949574	6.6033196
H	4.9705580	7.5852581	7.1549879
H	3.2555161	7.8194191	6.6711155
C	5.8640967	11.3306139	4.3670810
C	7.1461747	11.7885196	3.9718454
H	7.9917804	11.0987538	4.0349369
C	7.3013527	13.0891732	3.5342949
H	8.2899554	13.4549989	3.2370415
C	6.1800089	13.9634462	3.4347027
C	6.3158647	15.2848658	2.9155577
H	7.3128369	15.6286200	2.6177542
C	5.2157423	16.1130435	2.7742865
H	5.3330245	17.1234675	2.3686067
C	3.9282338	15.6421242	3.1407587
H	3.0546005	16.2886864	3.0045727
C	3.7633845	14.3688030	3.6629364
H	2.7630014	14.0177208	3.9297987
C	4.8776208	13.4951691	3.8455812
C	4.7390925	12.1598772	4.3795421
C	3.4444187	11.6347047	4.8976809
C	2.9357297	10.4439387	4.3734808
C	1.6744032	9.9160225	4.7520119
H	1.3326616	8.9935935	4.2749378
C	0.9185558	10.5756251	5.7017865
H	-0.0633091	10.1871322	5.9940975
C	1.4130696	11.7512535	6.3400983
C	0.6741868	12.3986940	7.3742245
H	-0.3054029	11.9918313	7.6501148
C	1.1839407	13.5062290	8.0301140
H	0.6088273	13.9900752	8.8265890
C	2.4660158	14.0041101	7.6801644
H	2.8805908	14.8632687	8.2182303
C	3.2032635	13.4091865	6.6684440
H	4.1942375	13.7964641	6.4175575
C	2.6992370	12.2819015	5.9524754
Cl	6.9529213	7.4317990	-0.0775775
N	4.6596904	7.7566734	5.0742399
O	5.7653167	10.0136936	4.8171930
O	3.6625469	9.7489955	3.4119390
P	5.0188541	8.8459575	3.8630827
Pd	5.9358144	8.0273554	1.9906947
O	11.2642023	7.5771854	2.6096008
N	10.1113918	7.8934199	0.6262034
C	3.9872643	10.1042919	-0.2554150
H	3.2051702	10.8690752	-0.0889602
H	4.5191262	10.3277023	-1.1987150
H	4.7115977	10.1542987	0.5746835
C	2.4596100	8.5197553	-1.4106434
H	2.1267487	7.4727025	-1.3963843
H	2.9587027	8.7167235	-2.3783206
H	1.5788500	9.1838319	-1.3239314
C	3.7353304	5.0263864	0.5300719
C	4.9365870	4.2733069	0.5288669
H	5.8139372	4.7004216	0.0345959
C	4.9606389	3.0387128	1.1489134
H	5.8775078	2.4391879	1.1458087
C	3.8102173	2.5376486	1.8270744
C	3.8462866	1.2961936	2.5286532
H	4.7729557	0.7118362	2.5062703
C	2.7461185	0.8416998	3.2357698
H	2.7901448	-0.1110376	3.7739852
C	1.5627409	1.6242037	3.2765895
H	0.7016851	1.2768720	3.8578781
C	1.4877617	2.8275191	2.5923937
H	0.5722884	3.4233916	2.6411339
C	2.5929300	3.3152650	1.8308218
C	2.5512180	4.5672925	1.1126445
C	1.3241777	5.4067270	1.0115503
C	1.3839850	6.7450276	1.4150620
C	0.2446317	7.5922286	1.4062885
H	0.3592130	8.6219037	1.7573305
C	-0.9702178	7.1049663	0.9650524
H	-1.8571330	7.7482314	0.9657133
C	-1.0846619	5.7719067	0.4722550
C	-2.3171452	5.2780596	-0.0483356
H	-3.1928449	5.9369660	-0.0305259
C	-2.4083161	4.0031934	-0.5800798
H	-3.3597822	3.6385410	-0.9815281
C	-1.2572626	3.1739210	-0.6213919
H	-1.3227272	2.1762187	-1.0685550

C	-0.0480455	3.6167784	-0.1089087
H	0.8320965	2.9702764	-0.1576562
C	0.0778535	4.9148088	0.4726176
N	3.4001724	8.7622998	-0.3127714
O	3.7445758	6.2587538	-0.1204909
O	2.5751933	7.2788299	1.8858219
P	3.8653276	7.6387820	0.8276855
H	9.1622410	7.8001853	0.2341211
H	12.7536448	8.3196869	1.1383751
C	12.4481619	8.5667367	0.1207742
C	11.0967929	8.3847067	-0.2503128
C	10.7047928	8.7074283	-1.5674155
H	9.6590216	8.5715748	-1.8656558
C	11.6382331	9.1963292	-2.4872862
H	11.3060733	9.4384735	-3.5041154
C	12.9886549	9.3826057	-2.1363375
C	14.0003409	9.9084971	-3.1316995
C	13.3635365	9.0571801	-0.8171644
H	14.4079271	9.1893061	-0.5081445
H	13.5298041	10.1243166	-4.1059306
H	14.8138187	9.1791520	-3.3046497
H	14.4743847	10.8409116	-2.7721473

L

C	10.9426369	1.3909857	3.3636875
H	11.4095979	0.4286193	3.6496427
H	11.7496767	2.1405637	3.2629931
H	10.4731514	1.2602825	2.3759328
C	10.4724364	2.0695872	5.6786135
H	9.6756086	2.4400308	6.3342559
H	11.2713961	2.8330814	5.6404837
H	10.8931677	1.1496091	6.1237544
C	6.5348864	3.1662143	5.3084370
C	5.7522703	4.1885672	4.7172501
H	6.2334627	4.8458608	3.9902728
C	4.4324579	4.3371961	5.0726958
H	3.8277589	5.1331487	4.6292636
C	3.8259193	3.4435134	5.9987854
C	2.4461635	3.5491531	6.3327123
H	1.8612461	4.3570432	5.8834001
C	1.8509776	2.6497735	7.1912860
H	0.7893996	2.7397371	7.4360883
C	2.6175922	1.5940383	7.7442042
H	2.1394790	0.8657511	8.4047345
C	3.9605369	1.4717480	7.4519548
H	4.5319487	0.6472975	7.8791426
C	4.6169156	2.3981196	6.5896684
C	6.0167555	2.3017645	6.2687256
C	6.9154968	1.2895035	6.8934288
C	7.6293599	0.4216086	6.0709458
C	8.4454880	-0.6097380	6.5998295
H	8.9514868	-1.2718165	5.8943210
C	8.5807469	-0.7536184	7.9599480
H	9.2016780	-1.5539522	8.3721925
C	7.9410731	0.1523136	8.8519153
C	8.1248606	0.0574334	10.2601927
H	8.7506965	-0.7500993	10.6512818
C	7.5415740	0.9664681	11.1169864
H	7.6939426	0.8845483	12.1963194
C	6.7533268	2.0201903	10.5915703
H	6.3095247	2.7546409	11.2690161
C	6.5442304	2.1323303	9.2321781
H	5.9410826	2.9542000	8.8455827
C	7.1109107	1.1975381	8.3170714
N	9.9545322	1.8226580	4.3389137
O	7.8510487	3.0658530	4.9232855
O	7.5674119	0.5451464	4.7061025
P	8.3344763	1.8202518	3.8719156

E(SCF)_B3LYP-D3 = -1394.292451
E(SCF)_SMD(THF)-B3LYP-D3 = -1396.282819
H_{corr}(298 K) = 968.58 kJ/mol
G_{corr}(298 K) = 782.76 kJ/mol

7-INT1-diss

C	6.0481468	0.4301359	2.4090379
H	6.7155864	-0.4047588	2.6688144
C	4.8210213	0.6636090	3.2213250
H	4.6890846	0.8624210	4.2867896
C	3.9729290	0.5142699	2.1800571
H	2.8855630	0.5440034	2.0674553
C	5.0879587	0.2544866	1.1660600
H	5.0834912	-0.7468053	0.6973546
C	5.3007403	1.3343934	0.1273495
C	4.5107112	2.1248683	-2.1218531
C	3.6350713	1.7779474	-3.1624064
H	3.0259876	0.8715897	-3.0851166
C	3.5317983	2.5805025	-4.2987888
H	2.8416322	2.2882455	-5.0950644
C	4.2948514	3.7495439	-4.4348470
C	5.1649819	4.0812828	-3.3835274
H	5.7737030	4.9872516	-3.4583203
C	5.2834691	3.2932385	-2.2384175
H	5.9659115	3.5756882	-1.4407775
C	4.1971782	4.6243808	-5.6604029
H	3.4416113	4.2522694	-6.3684556
H	5.1627517	4.6724459	-6.1921015
H	3.9272416	5.6591734	-5.3904023
Br	8.3425679	1.8645232	4.2675849
N	4.5613345	1.2601786	-0.9994195
H	3.9392062	0.4604605	-1.0518410
O	6.1158050	2.2518950	0.3430995
Pd	7.1724490	2.0630261	2.1839325

E(SCF)_B3LYP-D3 = -3295.357390
E(SCF)_SMD(THF)-B3LYP-D3 = -3296.916714
H_{corr}(298 K) = 604.00 kJ/mol
G_{corr}(298 K) = 431.20 kJ/mol

7-TS1-diss

(-79.87 cm^{-1})

C	5.9221092	0.1436891	2.1561536
H	6.6382299	-0.6304272	1.8612253
C	5.1712591	0.2978000	3.4053246
H	5.3707228	0.0076135	4.4386388
C	4.1206091	0.9226272	2.7740122
H	3.2036272	1.4056418	3.1177734
C	4.6219246	0.5696249	1.3714075
H	4.0925329	-0.3044934	0.9492988
C	4.7382181	1.7081554	0.3599114
C	4.4231553	2.1906733	-2.0836812
C	3.9823912	1.6079412	-3.2829792
H	3.6262882	0.5725850	-3.2882884
C	3.9924467	2.3368788	-4.4719512
H	3.6431941	1.8590427	-5.3917956
C	4.4406105	3.6655975	-4.5064386
C	4.8749898	4.2334799	-3.2979662
H	5.2291863	5.2687154	-3.2900332
C	4.8730815	3.5226308	-2.0973302
H	5.2148143	3.9855264	-1.1747077
C	4.4783443	4.4555888	-5.7917977
H	3.9089056	3.9591887	-6.5923636
H	5.5153954	4.5802833	-6.1496543
H	4.0617423	5.4667770	-5.6542527
Br	9.0628759	1.4982364	2.4850934
N	4.3880963	1.3911900	-0.9170203
H	4.0723910	0.4391148	-1.0637396
O	5.1354738	2.8121042	0.7192196
Pd	6.6900720	1.9244419	2.6530878

E(SCF)_B3LYP-D3 = -3295.330939
E(SCF)_SMD(THF)-B3LYP-D3 = -3296.893417
H_{corr}(298 K) = 600.38 kJ/mol
G_{corr}(298 K) = 427.66 kJ/mol

7-INT2-diss

C	5.9583606	0.2345440	2.3258787
H	7.0292464	0.0199154	2.3035601
C	4.9315287	-0.0075810	3.2757886
H	4.8684644	-0.5044528	4.2447313
C	3.9785168	0.6140117	2.3844034
H	2.8904647	0.7199260	2.4123636
C	4.9399446	0.3971231	1.1786563
H	4.7284525	-0.5661267	0.6738895
C	5.1754171	1.5074545	0.1512674
C	4.4698209	2.1524854	-2.1727662
C	3.7831785	1.6754596	-3.3011830
H	3.3039364	0.6912300	-3.2729548
C	3.7067685	2.4428954	-4.4638112
H	3.1668205	2.0461666	-5.3284473
C	4.3097440	3.7065900	-4.5417145
C	4.9935728	4.1678665	-3.4050605
H	5.4756766	5.1498032	-3.4324771
C	5.0823286	3.4171591	-2.2324630
H	5.6212118	3.7962578	-1.3672660
C	4.2230007	4.5525169	-5.7886017
H	3.7729976	3.9990255	-6.6267913
H	5.2197891	4.8996375	-6.1077666
H	3.6092687	5.4536408	-5.6160381
Br	4.2974650	4.2093313	3.9445232
N	4.5039466	1.3254827	-1.0265413
H	3.9609500	0.4727714	-1.1021618
O	5.9017568	2.4553160	0.4019458
Pd	5.1811186	2.0796657	3.2212953

E(SCF)_B3LYP-D3 = -3295.338390
E(SCF)_SMD(THF)-B3LYP-D3 = -3296.902561
H_{corr}(298 K) = 600.13 kJ/mol
G_{corr}(298 K) = 433.13 kJ/mol

7-INT3-diss

C	4.4564211	2.3296267	2.7151152
H	5.1544000	2.5232968	3.5349963
C	3.0121554	2.2647720	2.6558332
H	2.2030352	2.4226366	3.3702238
C	3.0971249	1.8501916	1.3044303
H	2.3558530	1.5960151	0.5422571
C	4.5702402	1.4009218	1.4765551
H	4.6071216	0.3387123	1.7761882
C	5.5654237	1.5044794	0.3115858
C	7.3030172	2.9791686	-0.7239696
C	7.8303546	4.2829337	-0.6652093
H	7.4684835	4.9791624	0.0968460
C	8.7909412	4.6940873	-1.5845780
H	9.1839341	5.7134237	-1.5209411
C	9.2581650	3.8315601	-2.5912692
C	8.7178777	2.5395186	-2.6371184
H	9.0578350	1.8432987	-3.4098710
C	7.7516515	2.1037362	-1.7250697
H	7.3406602	1.0991222	-1.7863039
C	10.2958775	4.2975136	-3.5833975
H	11.2066285	4.6554177	-3.0734184
H	9.9186330	5.1381375	-4.1913103
H	10.5901263	3.4918234	-4.2728057
Br	4.9622909	6.0203014	1.4663392
N	6.3432376	2.6241457	0.2536983
H	6.1486591	3.3551939	0.9337794
O	5.5969576	0.5802476	-0.4840031
Pd	3.7472095	3.9369080	1.6025630

E(SCF)_B3LYP-D3 = -3295.344243
E(SCF)_SMD(THF)-B3LYP-D3 = -3296.904467
H_{corr}(298 K) = 600.81 kJ/mol
G_{corr}(298 K) = 435.51 kJ/mol

References

(1) a) Becke, A. D. *J. Chem. Phys.* **1993**, *98*, 5648-5652. b) Lee, C.; Yang, W.; Parr, R. G. *Phys. Rev. B.* **1988**, *37*, 785-789.

(2) Tao, J.; Perdew, J. P.; Starovernov, V. N.; Scuseria, G. E. *Phys. Rev. Lett.* **2003**, *91*, 146401-146404. b) Staroverov, V. N.; Scuseria, G. E.; Tao, J.; Perdew, J. P. *J. Chem. Phys.* **2003**, *119*, 12129-12137.

(3) Grimme S.; Anthony, J.; Ehrlich, S.; Krieg, H. *J. Chem. Phys.* **2010**, *132*, 154104.

(4) a) Schäfer, A.; Horn, H.; Ahlrichs, R. *J. Chem. Phys.* **1992**, *97*, 2571-2577. b) Weigend, F.; Ahlrichs, R. *Phys. Chem. Chem. Phys.* **2005**, *7*, 3297-3305. c) Weigend, F. *Phys. Chem. Chem. Phys.* **2006**, *8*, 1057-1065.

(5) Andrae, D.; Haeusserman, U.; Dolg, M.; Stoll, H.; *Theor. Chim. Acta* **1990**, *77*, 123-141.

(6) a) Eichkorn, K.; Trutler, O.; Öhm, H.; Häser, M.; Ahlrichs, R. *Chem. Phys. Lett.* **1995**, *240*, 283-290. b) Eichkorn, K. Weigend, F.; Treutler, O.; Ahlrichs, R. *Chem. Phys. Lett.* **1995**, *242*, 652-660.

(7) Sierka, M.; Hogekamp, A.; Ahlrichs, R. *J. Chem. Phys.* **2003**, *118*, 9136-9148.

(8) Zhao, Y.; Truhlar, D. G. *J. Chem. Phys.* **2006**, *125*, 194101.

(9) Zhao, Y.; Truhlar, D. G. *Theor. Chem. Acc.* **2008**, *120*, 215-241.

(10) Marenich, A. V.; Cramer, C. J.; Truhlar, D. G. *J. Phys. Chem. B* **2009**, *113*, 6378-6396.

(11) a) Ahlrichs, R.; Bär, M.; Häser, M.; Horn, H.; Kölmel, C. *Chem. Phys. Lett.* **1989**, *162*, 165-169. b) TURBOMOLE V6.4 2012, a development of University of Karlsruhe and Forschungszentrum Karlsruhe GmbH, **1989-2007**, TURBOMOLE GmbH, since 2007; available from http://www.turbomole.com

(12) Gaussian 09, Revision D.01, M. J. Frisch, G. W. Trucks, H. B. Schlegel, G. E. Scuseria, M. A. Robb, J. R. Cheeseman, G. Scalmani, V. Barone, B. Mennucci, G. A. Petersson, H. Nakatsuji, M. Caricato, X. Li, H. P. Hratchian, A. F. Izmaylov, J. Bloino, G. Zheng, J. L. Sonnenberg, M. Hada, M. Ehara, K. Toyota, R. Fukuda, J. Hasegawa, M. Ishida, T. Nakajima, Y. Honda, O. Kitao, H. Nakai, T. Vreven, J. A. Montgomery, Jr., J. E. Peralta, F. Ogliaro, M. Bearpark, J. J. Heyd, E. Brothers, K. N. Kudin, V. N. Staroverov, T. Keith, R. Kobayashi, J. Normand, K. Raghavachari, A. Rendell, J. C. Burant, S. S. Iyengar, J. Tomasi, M. Cossi, N. Rega, J. M. Millam, M. Klene, J. E. Knox, J. B. Cross, V. Bakken, C. Adamo, J. Jaramillo, R. Gomperts, R. E. Stratmann, O. Yazyev, A. J. Austin, R. Cammi, C. Pomelli, J. W. Ochterski, R. L. Martin, K. Morokuma, V. G. Zakrzewski, G. A. Voth, P. Salvador, J. J. Dannenberg, S. Dapprich, A. D. Daniels, O.

Farkas, J. B. Foresman, J. V. Ortiz, J. Cioslowski, and D. J. Fox, Gaussian, Inc., Wallingford CT, 2013.
